# Heterologous Production and Biosynthesis of Threonine-16:0dioic acids with a Hydroxamate Moiety

**DOI:** 10.1021/acs.jnatprod.3c00097

**Published:** 2023-09-20

**Authors:** Marc Stierhof, Maksym Myronovskyi, Josef Zapp, Andriy Luzhetskyy

**Affiliations:** ^†^Department of Pharmaceutical Biotechnology and ^‡^Department of Pharmaceutical Biology, Saarland University, 66123 Saarbruecken, Germany; § Helmholtz Institute for Pharmaceutical Research Saarland, Saarland University, 66123 Saarbruecken, Germany

## Abstract

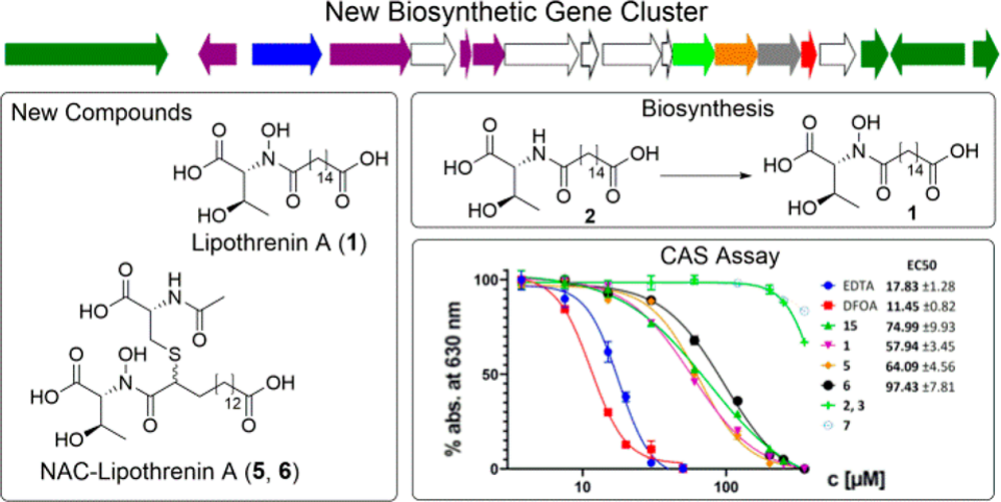

Dereplication and genome mining in *Streptomyces aureus* LU18118 combined with heterologous expression of selected biosynthetic gene clusters (BGCs) led to the discovery of various threonine-16:0dioic acids named lipothrenins. Lipothrenins consist of the core elements l-Thr, d-*allo*-Thr, or Dhb, which are linked to hexadecanedioic acid by an amide bond. The main compound lipothrenin A (**1**) carries the *N*-hydroxylated d-*allo* form of threonine and expresses a siderophore activity. The lipothrenin BGC was analyzed by a series of deletion experiments. As a result, a variety of interesting genes involved in the recruitment and selective activation of linear 16:0dioic acids, amide bond formation, and the epimerization of l-Thr were revealed. Furthermore, a diiron *N*-oxygenase was identified that may be directly involved in the monooxygenation of the amide bond. This is divergent from the usual hydroxamate formation mechanism in siderophores, which involves hydroxylation of the free amine prior to amide bond formation. Siderophore activity was observed for all *N*-hydroxylated lipothrenins by application of the CAS assay method.

Natural products are one of the best sources of complex chemical scaffolds, making them an important factor in drug discovery, especially for infectious diseases and cancer, but also for immunosuppression (tacrolimus, myriocin) and cardiovascular diseases (statins).^1−6^ The reason for the enormous structural variability of natural products lies in the constant evolution of organisms driven by the quest to achieve an evolutionary advantage.^7^ Microorganisms are especially talented in adaptation to changing external conditions. Microbial environments are highly competitive and require a special survival mechanism that involves the production of bioactive secondary metabolites with unique chemical scaffolds.^8^

One of these mechanisms is to ensure an adequate supply of iron, which is important for the growth of most bacteria. At physiological pH the iron uptake is limited due to the low solubility of Fe^3+^ ions. In these conditions, iron capture and transport through the bacterial cell membrane is facilitated by the production and secretion of iron-chelating small molecules, known as siderophores. Siderophores have found application in the development of antibiotic delivery methodologies,^9^ growth of uncultivable microorganisms,^10^ and agriculture,^11^ hence presenting an important class of small molecules. Among microorganisms, *Streptomyces* are a source of a variety of different siderophores including deferoxamine,^12^ enterobactin,^13^ and qinichelins.^14^ The potential of streptomycetes as a valuable source of siderophores is of high interest in the discovery of antibiotics and antitumor drugs and in the treatment of iron poisoning.^15^ The naturally occurring “Trojan Horse” antibiotic salmycin and the antitumor-active deferoxamine are two examples to mention.^16,17^

In the beginning of the Golden Age of antibiotic discovery, bioactivity-guided isolation of secondary metabolites was an effective method, and this approach still applies today for unexplored strains.^18,19^ However, especially for thoroughly investigated strains such as *Streptomyces*, the chance of rediscovery of already known natural products derived from this genus is rather high. The last three decades of technical evolution have significantly changed this situation and the landscape of natural product research by improving instrumental analytics and the development of genome sequencing. These developments led to the discovery of cryptic biosynthetic gene clusters (BGCs) in *Streptomyces coelicolor* two decades ago and revealed an abundance of new natural products hidden in the genomes of streptomycetes.^20^ Previous work has shown that activation of cryptic BGCs by heterologous expression in optimized host strains such as *Streptomyces lividans* Δ8 and *Streptomyces albus* Δ14 is a powerful tool to unlock this potential.^21−25^ In this work, genome mining, heterologous expression, and dereplication were used to identify new natural product scaffolds from the less studied strain *Streptomyces aureus* LU18118. This led to the discovery of new threonine-16:0dioic acids named lipothrenins and the corresponding BGC, named *lit*-cluster. The borders of the *lit*-cluster and the biosynthetic genes that are involved in the biosynthesis were determined by gene deletion experiments. Lipothenins with hydroxamate moieties showed siderophore activities determined by the CAS assay method.

## Results and Discussion

### Isolation, Heterologous Expression, and Structure Elucidation of Lipothrenins

**Figure 1:**
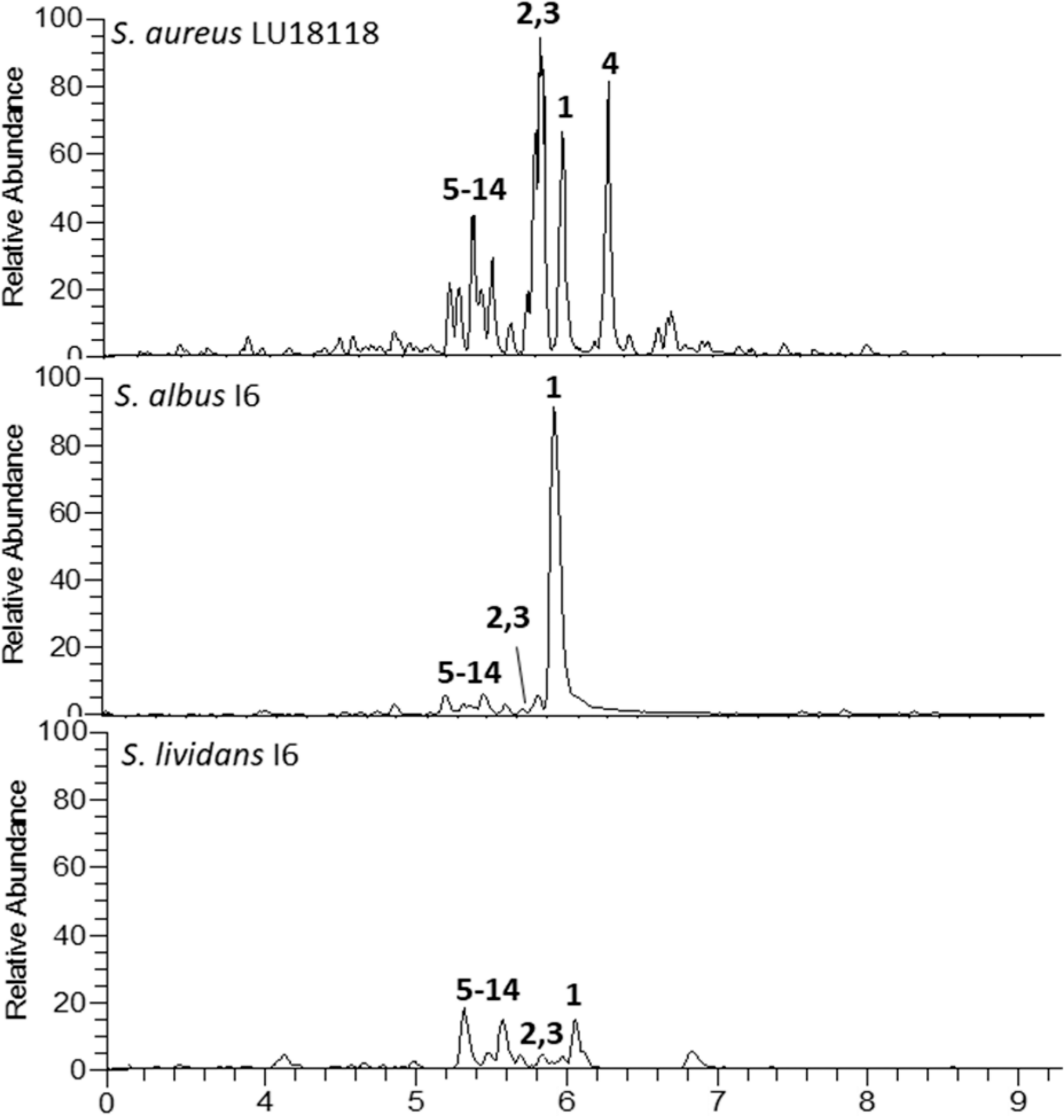
LC–MS chromatograms of *n*-butanol extracts of *S. aureus* LU18118, *S. albus* I6, and *S. lividans* I6 showing new peaks **1**–**14**.

Fermentation of *S. aureus* LU18118 in DNPM (dextrin, N-Z soy BL, primary yeast, MOPS buffer) medium and metabolic analysis of the butanol extracts through LC–MS and dereplication revealed a variety of new peaks with masses ranging from 370 to 565 Da and UV–vis absorption at 206 nm (Figure 1).

In silico genomic analysis of LU8118 by antiSMASH revealed a variety of BGCs encoding potential new natural products. From a bacterial artificial chromosome (BAC) library constructed for *S. aureus* LU18118, 13 BACs that cover 21 unknown BGCs were chosen for heterologous expression in the optimized host strains *S. lividans* Δ8 and *S. albus* Δ14. BACs were transferred into the heterologous hosts; the mutants were cultivated in DNPM production medium and extracted with *n*-butanol, and the production of new metabolites was screened by LC–MS. BAC I6 was successfully expressed in both strains, while production in *S. albus* Δ14 was several magnitudes higher than that in *S. lividans* Δ8 (Figure 1). The produced compounds matched the previously identified masses from the dereplication of *S. aureus* LU18118 Da, suggesting that BAC I6 is responsible for the production. The compounds can be divided into two groups due to the mass differences of 161 Da: *m*/*z* ([M + H]^+^) 404.266 (**1**), 388.271 (**2**, **3**), and 370.260 (**4**) Da and *m*/*z* 565.278 (**5**, **6**), 549.284 (**7**–**10**), and 531.272 (**11**–**14**) Da. Compounds **1**–**14** were obtained from a large-scale fermentation of LU18118, which was performed in parallel with the heterologous expression procedure. All structures were determined by extensive analysis of 1D and 2D NMR (Figures S9–S75), and the absolute configuration was determined by the advanced Marfey’s method (Figures S4, S5).

**Figure 2:**
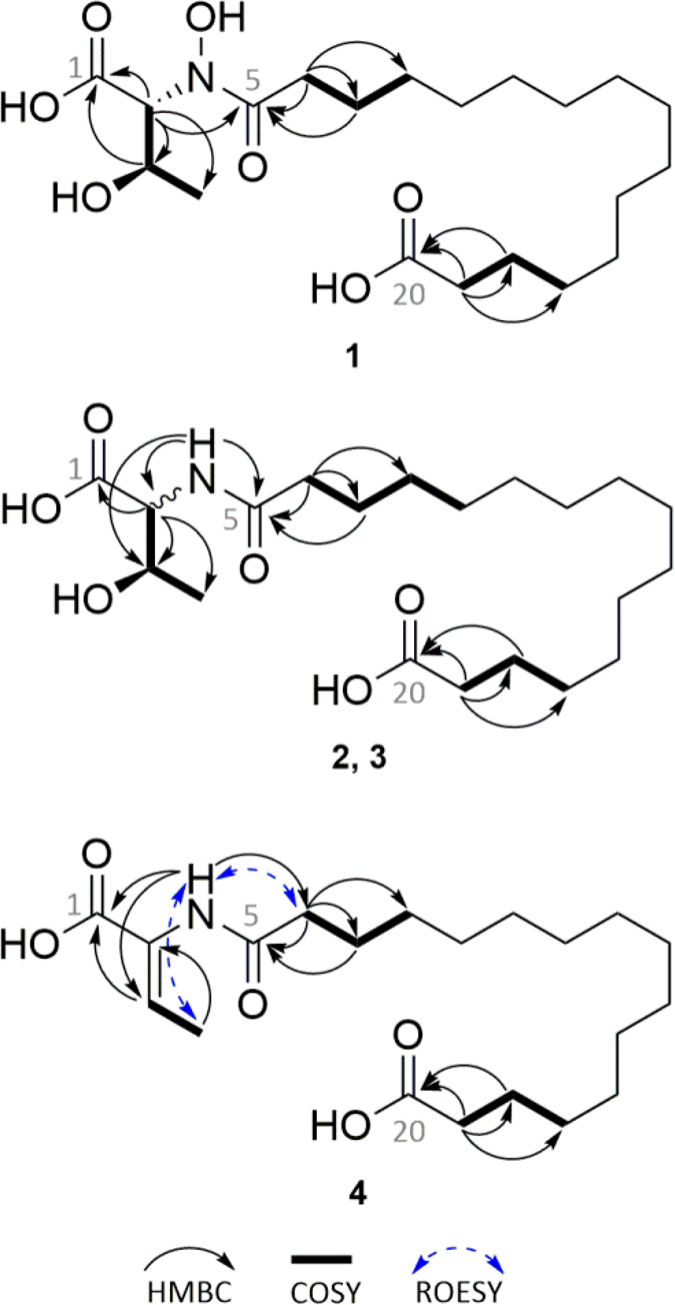
Structure of comppunds **1**–**4** with selected COSY (**―**), HMBC (↷), and ROESY (↔) correlations.

The molecular formula of **1** was calculated as C_20_H_37_NO_7_ with 3 degrees of unsaturation based on HRESIMS data. Proton NMR data revealed a very large singlet at δ_H_ 1.25, which is common for the long methylene chain of fatty acids (Table 1). The high field shifted doublet of the ω-methyl group was absent, indicating another moiety at this position. HMBC correlations from the fatty-acid-chain-associated methylenes CH_2_-18 and CH_2_-19 to δ_C_ 174.5 revealed a carboxyl group in the ω-position (Figure 2). Methylene CH_2_-6 showed peak splitting, indicating a rigid structure attached to the adjacent carboxylate δ_C_ 173.5 (C-5). The remaining proton and carbon signals were assigned to one methyl, two methines, and a carbon signal at δ_C_ 171.6. The spin system was assigned to threonine, which showed a connection to the fatty acid chain by an HMBC correlation of the α-CH to δ_C_ 173.5 (Figure 2). Based on the molecular formula and the remaining ^13^C NMR signals, the fatty acid chain was determined to possess 14 methylenes and two carboxyl groups, which led to the identification of hexadecanedioic acid. Despite using DMSO-*d*_6_ as a solvent, no NH proton signals were observed. The unusually highly shifted α-CH at δ_C_ 63.3 and the remaining unassigned oxygen from the calculated formula suggest that threonine carries an *N*-hydroxy group. Similar chemical shifts for *N*-hydroxy threonine have been previously described in the literature.^26^ Furthermore, threonine might be able to adopt a six-membered conformation stabilized through a hydrogen bridge between N-OH and the carboxyl group of threonine. The resulting rigid conformation would explain the observed peak splitting of methylene C-6 of the fatty acid chain and provide further proof for the hydroxamate moiety. The new compound has no similarity to other lipo-amino acids and was named lipothrenin A (**1**).

**Table 1:** ^1^H and ^13^C NMR Data of Compounds **1**–**4** at 500/125 MHz in DMSO

	**1**		**2**		**3**		**4**	
position	δ_C_, type	δ_H_ (*J* in Hz)	δ_C_	δ_H_ (*J* in Hz)	δ_C_	δ_H_ (*J* in Hz)	δ_C_, type	δ_H_ (*J* in Hz)
1	171.6, C		172.4		172.4		165.8, C	
2	63.3, CH	4.50, d (8.8)	58.3	4.15, dd (8.4, 6.3)	57.4	4.19, dd (3.4, 8.7)	129.0, C	
3	64.8, CH	4.03, dq (6.3, 8.8)	66.8	3.84, q (6.3)	66.3	4.09, dq (3.4, 6.3)	130.6, CH	6.46, q (7.1)
4	20.4, CH_3_	1.01, d (6.3)	19.8	1.06, d (6.4)	20.3	1.03, d (6.4)	13.5, CH_3_	1.62, d (7.1)
5	173.5, C		172.3		172.6		171.0, C	
6	31.6, CH_2_	2.25–2.42, m	35.1	2.17, 2.12, m	35.1	2.17, 2.12, m	35.1, CH_2_	2.19, t (7.3)
7	24.2, CH_2_	1.47, m	25.3	1.47, m	25.3	1.47, m	25.2, CH_2_	1.51, m
8–17	28.6–29.1, CH_2_	1.25, bs	28.6–29.1	1.23, bs	28.6–29.1,	1.23, bs	28.5–29.0, CH_2_	1.30, bs (
18	24.5, CH_2_	1.47, m	24.5	1.47, m	24.5	1.47, m	24.5, CH_2_	1.47, m
19	33.7, CH_2_	2.18, t (7.4)	33.7	2.18, t (7.5)	33.7	2.18, t (7.5)	33.7, CH_2_	2.18, t (7.3)
20	174.5, C				174.6		174.5, C	
NH (1)				7.86, d (8.2)		7.66, d (8.7)		8.92, s

Compounds **2** and **3** were purified as a mixture. The molecular formula of **2** and **3** was calculated as C_20_H_37_NO_6_ with 3 degrees of unsaturation based on HRESIMS data. Proton NMR revealed a mixture of two compounds highly similar to **1** but showed additional NH signals at δ_H_ values of 7.86 and 7.66. Compounds **2** and **3** showed differences in the shifts, coupling constants, and integrals ratio (1:0.4, Figure S17) of the threonine moiety, indicating a mixture of two stereoisomers (Table 1). This was analyzed by Marfey’s method.^27^ Compounds **1**, **2**, and **3** were hydrolyzed with 6 N hydrochloric acid, and the hydrolysis product, together with the reference amino acids l-Thr, d-Thr, l-*allo*-Thr, and d-*allo*-Thr, was derivatized with l-FDLA. Subsequent LC–MS measurements identified d-*allo*-Thr in lipothrenin A (**1**), while in the mixture of **2** and **3**, d-*allo*-Thr and l-Thr were identified (Figure S4). Compared to **1**, the compounds lack the *N*-hydroxy group and are named d-lipothrenin B (**2**) and l-lipothrenin B (**3**) (Figure 2).

The molecular formula of **4** was calculated as C_20_H_35_NO_5_ with 4 degrees of unsaturation based on HRESIMS data. In the ^13^C NMR spectra, two signals appeared at δ_C_ 129.0 and 130.6, while previously observed CH-2 and CH-3 signals in the ^1^H NMR data shifted or vanished (Table 1). The new spin system was established by HMBC and ROESY correlations of NH (Figure 2), revealing *Z*-dehydrobutyrine (Dhb). The compound was named *Z*-lipothrenin C (**4**).

**Figure 3:**
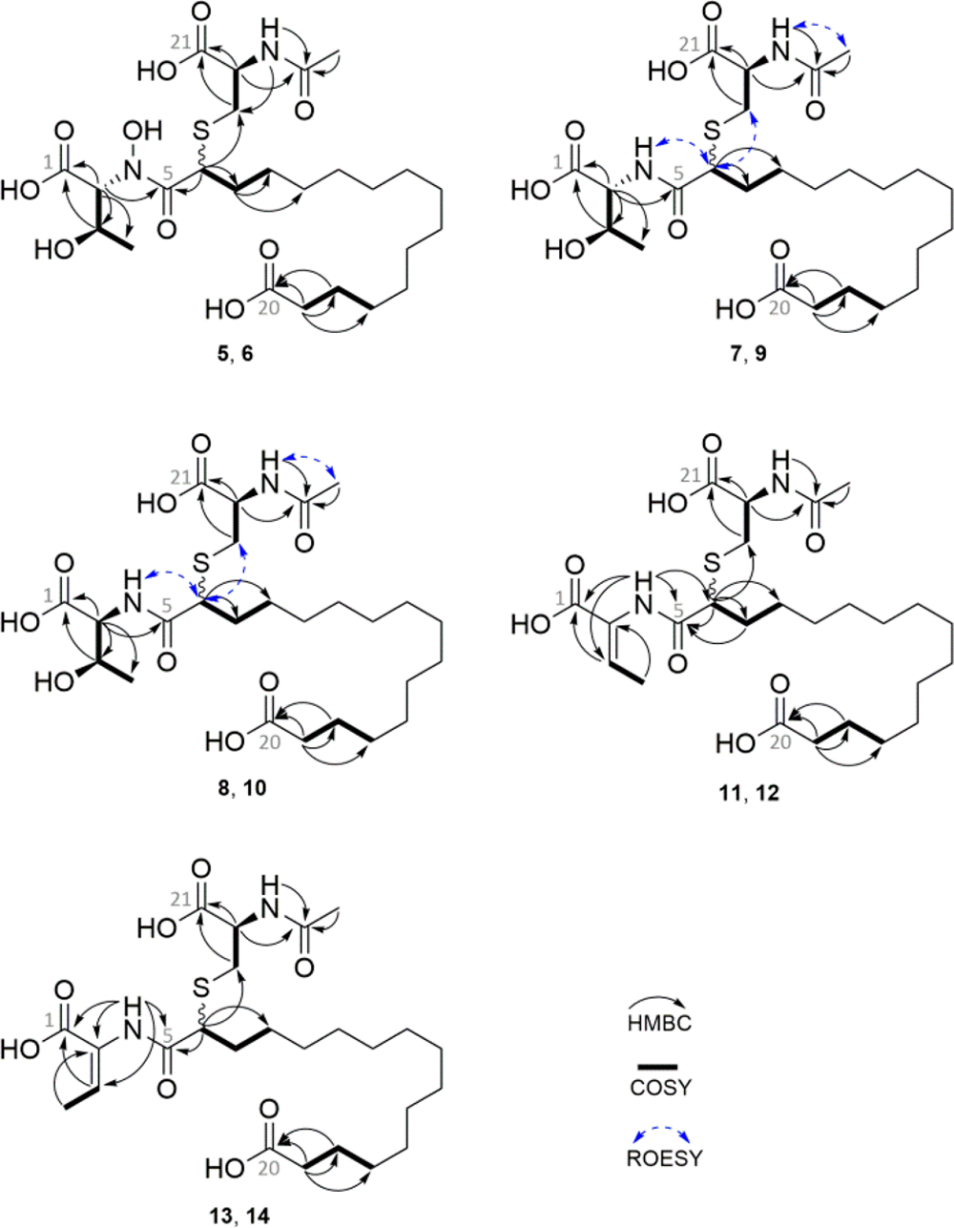
Structure of compounds **5**–**14** with selected COSY (**―**), HMBC (↷), and ROESY (↔) correlations.

The molecular formulas of the higher molecular weight compounds were calculated as C_25_H_44_N_2_O_10_S (**5**, **6**), C_25_H_44_N_2_O_9_S (**7**–**10**), and C_25_H_42_N_2_O_8_S (**11**–**14**) based on *m*/*z* ([M + H]^+^) 564.271 (**5**, **6**), 548.277 (**7**–**10**), and 530.266 (**11**–**14**) Da. The molecular formulas of compounds **5**–**14** showed a difference in C_5_H_7_NO_3_S compared to those of compounds **1**–**4**, indicating that both groups are structurally related. Analysis of the ^1^H NMR spectra of **5** and **6** revealed previously described signals for lipothrenin A (**1**) and additional signals at δ_H_ 8.21 (NH), 4.31 (CH), 2.97 (CH_2_a), 2.74 (CH_2_b), and 1.84 (CH_3_) (Tables 2, 3). The signals were assigned to *N*-acetylcysteine, which was determined at position CH-6 of the fatty acid chain through the analysis of COSY and HMBC correlations (Figure 3). Compounds **7**–**10** showed the same *N*-acetylcysteine modification and were assigned as the *N*-acetylcysteine derivatives of **2** and **3**.

**Table 2:** ^1^H NMR Data of Compounds **5**–**10** at 500 MHz in DMS

	**5**	**6**	**7**	**8**	**9**	**10**
position	δ_H_ (*J* in Hz)	δ_H_ (*J* in Hz)	δ_H_ (*J* in Hz)	δ_H_ (*J* in Hz)	δ_H_ (*J* in Hz)	δ_H_ (*J* in Hz)
1						
2	4.61, d (8.5)	4.62, d (8.5)	4.16, dd (6.0, 8.3)	4.25, dd (3.0, 9.0)	4.19, dd (6.3, 8.2)	4.20, dd (3.6, 6.3)
3	4.14, m (6.3, 8.5)	4.18, m (6.6, 8.8)	3.87, m (6.3)	4.16, dq (3.2, 6.6)	3.87, m (6.3)	4.16, dq (3.0, 6.6)
4	1.09, d (6.3)	1.07, d (6.3)	1.11, d (6.4)	1.03, d (6.4)	1.08, d (6.0)	1.07, d (6.6)
5						
6	3.92 dd (6.0, 9.0)	3.98 dd (6.3, 8.5)	3.43 dd (6.0, 9.0)	3.57, dd (5.8, 9.4)	3.42 dd (5.9, 8.9)	3.54, dd (6.0, 8.8)
7	1.58, m,	1.56, m	1.69, m	1.69, m	1.69, m	1.69, m
	1.76, m	1.76, m	1.50, m	1.49, m	1.49, m	1.49, m
8	1.31, m	1.30, m	1.25, m	1.25, m	1.22, m	1.22, m
9–17	1.23, bs	1.23, bs	1.23, bs	1.23, bs	1.23, bs	1.23, bs
18	1.47, m	1.47, m	1.47, m	1.47, m	1.47, m	1.47, m
19	2.18, t (7.4)	2.18, t (7.4)	2.18, t (7.4)	2.18 (7.4)	2.18, t (7.4)	2.18, t (7.4)
20						
21						
22	4.31, dt (5.4, 8.2)	4.40, dt (4.7, 8.5)	4.32, dt (5.1, 8.2)	4.33, dt (5.2, 8.3)	4.37, m	4.37, m
23α	2.97, dd (5.2, 13.4)	3.07, dd (4.9, 13.4)	2.94, dd (5.2, 13.4)	2.96, dd (5.1, 13.5)	2.99, dd (4.7, 13.2)	2.99, dd (4.7, 13.2)
β	2.74, dd (8.6, 13.3)	2.78, dd (9.0, 13.4)	2.78, dd (8.6, 13.3)	2.88, dd (8.7, 13.6)	2.74, m	2.73, m
24						
25	1.84, s	1.86, s	1.84, s	1.85, s	1.84, s	1.84, s
NH (1)			8.09, d (8.1)	7.98, d (9.0)	8.13, d (8.8)	7.95, d (8.8)
NH (2)	8.21, d (8.2)	8.17, d (7.9)	8.19, d (7.9)	8.19, d (7.9)	8.18, d (8.0)	8.18, d (8.0)

**Table 3:** ^13^C NMR Data of Compounds **5**–**10** at 125 MHz in DMSO

	**5**	**6**	**7**	**8**	**9**	**10**
position	δ_C_, type	δ_C_	δ_C_	δ_C_	δ_C_	δ_C_
1	170.4, C	170.5	171.2	171.4	171.9	172.1
2	63.5, CH	64.1	57.8	56.9	58.4	57.6
3	66.4, CH	64.5	66.2	65.6	66.8	66.3
4	20.1, CH_3_	20.1	19.2	19.6	19.7	20.4
5	171.9, C	172.3	170.7	171.0	171.3	171.9
6	41.4, CH	41.6	46.2	46.4	46.6	46.8
7	31.5, CH_2_	31.7	31.3	31.2	31.8	31.8
8	26.7, CH_2_	26.8	26.0	26.2	26.7	26.7
9–17	28.6–29.1, CH_2_	28.7–29.2	27.8–28.9	27.8–28.9	28.6–29.1	28.6–29.1
18	24.5, CH_2_	24.6	23.8	23.8	24.5	24.5
19	33.7, CH_2_	33.7	33.0	33.0	33.7	33.7
20	174.6, C	174.6	173.8	173.8	174.5	174.5
21	172.0, C	172.3	171.5	171.5	172.1	172.1
22	52.5, CH	52.1	51.8	52.1	51.7	51.7
23	31.1, CH_2_	31.4	31.2	31.1	31.7	31.7
24	169.4, C	169.4	168.6	168.6	169.3	169.3
25	22.4, CH_3_	22.2	21.8	21.9	22.3	22.3

The *N*-acetylcysteine modifications introduce two additional stereocenters at the CH-α of cysteine or at CH-6 of the fatty acid that would lead to four diastereomers. The appearance of two diastereomeric pairs of *N*-acetylcysteine-lipothrenins suggests that only one of the additional stereocenters is present in both orientations. The absolute configurations of threonine and cysteine were identified by Marfey’s method. *N*-Acetylcysteine-lipothrenins **7**–**10** were digested by 6 N HCl, derivatized with d- and l-FDLA and analyzed by LC–MS. Compounds **7** and **8** revealed d-*allo*-Thr and l-Thr, respectively, while the compound **9/10** revealed a mixture (Figure S4). Analysis of the coupling constant of CH-2 and CH-3 showed a difference between d-*allo*-Thr (*J* = 3–3.5 Hz) and l-Thr (*J* = 6–6.3 Hz) (Table 2). The specific coupling constants of d-*allo*-Thr and l-Thr (Table 1) were used to retrospectively annotate the absolute configurations of **2** and **3** (Figure S95).

The sulfur bond to C-6 is not cleaved by acid hydrolysis with HCl, since reductive conditions are required.^28^ Therefore, the 2,4-dinitrophenyl-l-leucinamide-cys-fatty acid (DLA-cys-fatty acid) conjugate (Figure S5) was used to determine differences in the retention times of DLA-d-cys-fatty acid and DLA-l-cys-fatty acid. Derivatization of hydrolysates **7**–**10** with l-FDLA did not lead to retention time differences when observing the extracted mass of DLA-cys-fatty acid. The same result was obtained after derivatization with d-FDLA, indicating that only one cysteine isomer is present in *N*-acetylcysteine-lipothrenin. However, there was a difference between l- and d-FDLA-derivatized cys-fatty acid, indicating that Marfey’s method also applies to the DLA-cys-fatty acid conjugate. The retention time of l-DLA-cys-fatty acid was shorter than that of the d-DLA-cys-fatty acid conjugate. Therefore, cysteine is likely in the l-configuration, and the appearance of stereoisomers originates from the stereocenter at CH-6. Compounds **11**–**14** were assigned as the *N*-acetylcysteine derivatives of lipothrenin C (Figure 3). *N*-Acetylcysteine-lipothrenins **11** and **12** eluted simultaneously and appeared as a mixture in the NMR measurements. The same result was obtained for compounds **13** and **14**. Differences between **11**/**12** and **13**/**14** were determined by the chemical shift of CH-2 of Dhb. Similar to **4**, compounds **11**/**12** showed a shift of δ_C_ 131.8, indicating a *Z*-configuration (Table 4). Compounds **13**/**14** showed a shift of δ_C_ 125.0, which could be due to the isomerization to the *E*-conformer. This was confirmed by comparisons with predicted data from ACD Laboratories (δ_CH-2_(*E*) = 124.0, δ_CH-2_(*Z*) = 130.4).

**Table 4:** ^1^H and ^13^C NMR Data of Compounds **11**–**14** at 500/125 MHz in DMSO

	**11**		**12**		**13**		**14**	
position	δ_C_, type	δ_H_ (*J* in Hz)	δ_C_	δ_H_ (*J* in Hz)	δ_C_	δ_H_ (*J* in Hz)	δ_C_	δ_H_ (*J* in Hz)
1	165.5, C		165.5		165.2		165.2	
2	128.5, C		128.4		128.2		128.2	
3	131.8, CH	6.54, q (7.0)	131.7	6.54, q (7.0)	125	6.16, q (7.6)	125	6.16, q (7.6)
4	13.5, CH_3_	1.65, d (6.9)	13.5	1.65, d (6.9)	13.1	1.90, d (7.6)	13.1	1.90, d (7.6)
5	170.1, C		170		169.8		169.8	
6	46.6, CH	3.44, dd (5.0, 9.5)	46.8	3.44, dd (5.0, 9.5)	47	3.38, dd (6.0, 8.7)	47	3.38, dd (6.0, 8.7)
7	31.6, CH_2_	1.73, m	31.6	1.73, m	31.4	1.70, m	31.4	1.70, m
		1.51, m		1.51, m		1.50, m		1.50, m
8	26.8, CH_2_	1.29, m	26.8	1.29, m	26.3	1.25, m	26.3	1.25, m
9–17	28.6–29.0, CH_2_	1.23, bs	28.6–29.0	1.23, bs	27.8–28.8	1.23, bs	27.8–28.8	1.23, bs
18	24.5, CH_2_	1.47, m	24.5	1.47, m	24.2	1.46, m	24.2	1.46, m
19	33.7, CH_2_	2.18, (7.4)	33.7	2.18, (7.4)	33.4	2.18, (7.5)	33.4	2.18, (7.5)
20	174.5, C		174.5		174.1		174.1	
21	172.2, C		172.1		171.7		171.7	
22	51.8, CH	4.38, m	52.5	4.32, m	51.7	4.36, m	52.3	4.30, m
23α	31.9, CH_2_	3.04, dd (4.7, 13.2)	31.8	2.99, dd (5.4, 13.3)	31.7	2.97, dd (4.7, 13.3)	31.5	2.95, dd (5.6, 13.7)
β		2.78, dd (9.1, 13.2)		2.89, dd (8.2, 13.3)		2.73, dd (9.3, 13.3)		2.81, dd (8.3, 13.2)
24	169.2, C		169.2		168.9		168.9	
25	22.37, CH_3_	1.841, s	22.36	1.839, s	22.3	1.84, s	22.3	1.84, s
NH (1)		9.19, s		9.19, s		9.38, s		9.37, s
NH (2)		8.15, d (7.9)		8.15, d (7.9)		8.18, d (8.6)		8.17, d (8.6)

### Gene Deletion Experiments Revealed the Biosynthetic Pathway of Lipothrenins

BAC I6 contains a 51 kb chromosomal fragment of *S. aureus* LU18118, while the annotated fatty acid cluster comprises 33 kb of the fragment. Many genes from the chromosomal fragment have no annotation for fatty acid biosynthesis and might not be involved in the biosynthesis of lipothrenins. To experimentally determine the minimal set of lipothrenin biosynthetic genes, sequential gene deletions of the left and right flanking regions of the chromosomal fragment were performed. Modified BACs were transferred into *S. albus* Δ14, leading to the creation of *S. albus* I6_Δ mutant strains, hereafter called I6_Δ for simplicity (Table S1). Mutant strains were cultivated in DNPM, and metabolite production was monitored by LC–MS (Figures S1–S3).

**Figure 4:**
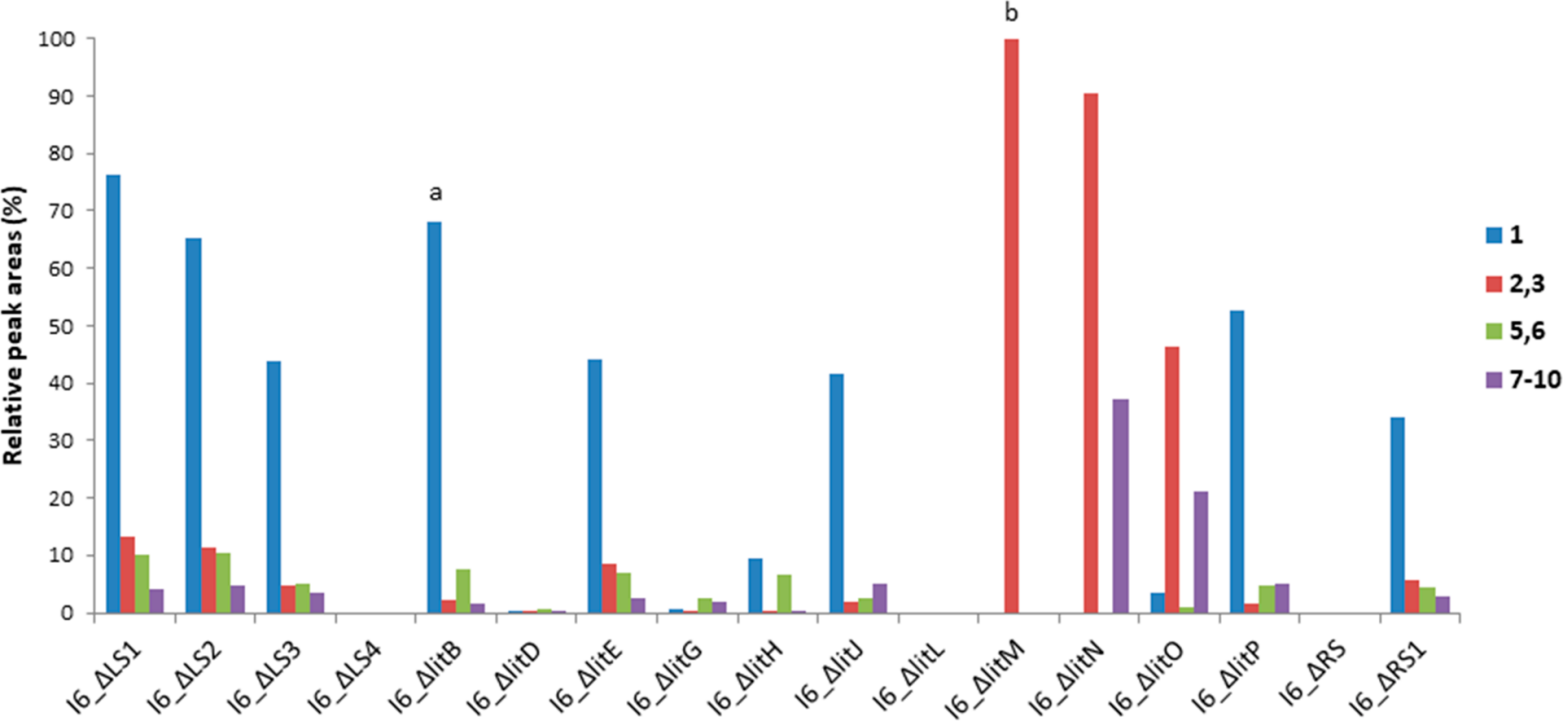
LC–MS production comparison of lipothrenins A (**1**, blue) and B (**2**, **3**, red) and *N*-acetylcysteine-lipothrenins A (**5**, **6**, green) and B (**7**–**10**, purple) in the *S. albus* I6 deletion mutants: (a) lipothrenin **15** included; (b) only l-lipothrenin B (**3**).

The fatty acid cluster location was annotated by antiSMASH between 16 and 49 kb in the BAC insert. The genes *litA* and *litR* encode an OmpR-like winged helix-turn-helix domain-containing protein, a family of *Streptomyces* antibiotic regulatory proteins (SARP).^29^ Nuclease-encoding genes *orf-02* and *orf-01* are likely not directly involved in lipothrenin biosynthesis, while *litQ* (transcriptional regulator) and *litS* (sigma 70 factor) might play a regulatory role (Table 5). To determine the left and right *lit*-BGC cluster borders, deletion mutants I6_ΔRS (*litQ*–*orf01*), I6_ΔRS1 (*orf01*–*orf04*), I6_ΔLS1 (*orf-24*–*orf-11*), I6_ΔLS2 (*orf-24*–*orf-09*), I6_ΔLS3 (*orf-24*–*orf-03*), and I6_ΔLS4 (*orf-24*–*litA*) were created (Table S1). The production of compounds **1**–**14** in deletion mutants I6_ΔRS1, I6_ΔLS1, I6_ΔLS2, and I6_ΔLS3 was not altered, while the production of compounds **1**–**14** in mutants I6_ΔRS and I6_ΔLS4 was abolished (Figure S1, Figure 4). This suggests that regulators encoded in *litA* and *litQ*–*litS* are involved in *lit* gene expression, indicating that the minimal set of genes required for lipothrenin biosynthesis is located between *litA* and *litS* (Table 5).

**Table 5:** Lipothrenin Biosynthetic Genes^a^

gene	aa length	putative function	coverage/identity	accession
*orf-02*	997	nuclease SbcCD subunit C	99/97	GHE70600
*orf-01*	338	exonuclease SbcCD subunit D	99/99	WP_136237380
*litA*	1168	regulatory protein AfsR	95/99	WP_028959863
*litB*	269	beta-ketoacyl ACP synthase III	99/98	WP_210637060
*litC*	496	MFS transporter permease	99/99	WP_210637059
*litD*	573	fatty-acyl AMP ligase	99/89	WP_069171638
*litE*	326	ribonucleotide-diphosphate reductase	99/99	WP_137209500
*litF*	88	acyl carrier protein	98/91	WP_202278898
*litG*	228	GDSL-like lipase/acylhydrolase	99/92	WP_202278897
*litH*	539	GMC family oxidoreductase	99/99	WP_210637056
*litI*	124	hypothetical protein	99/82	WP_202278895
*litJ*	421	Acyl-CoA dehydrogenase	99/96	WP_202278894
*litK*	85	hypothetical protein		
*litL*	311	hypothetical protein	99/99	WP_210637055
*litM*	297	ThiF family adenylyltransferase	99/99	WP_161108191
*litN*	311	diiron N-oxygenase	99/97	WP_069171647
*litO*	110	DUF4873 domain containing protein	99/98	WP_210637054
*litP*	256	dienelactone hydrolase	99/98	WP_028959855
*litQ*	205	TetR family transcriptional regulator	99/94	WP_069171650
*litR*	526	regulatory protein AfsR	98/98	WP_136237385
*litS*	181	sigma-70 factor	99/98	WP_164555762

a The sequence has been deposited in GenBank with accession number ON169987.

**Figure 5:**
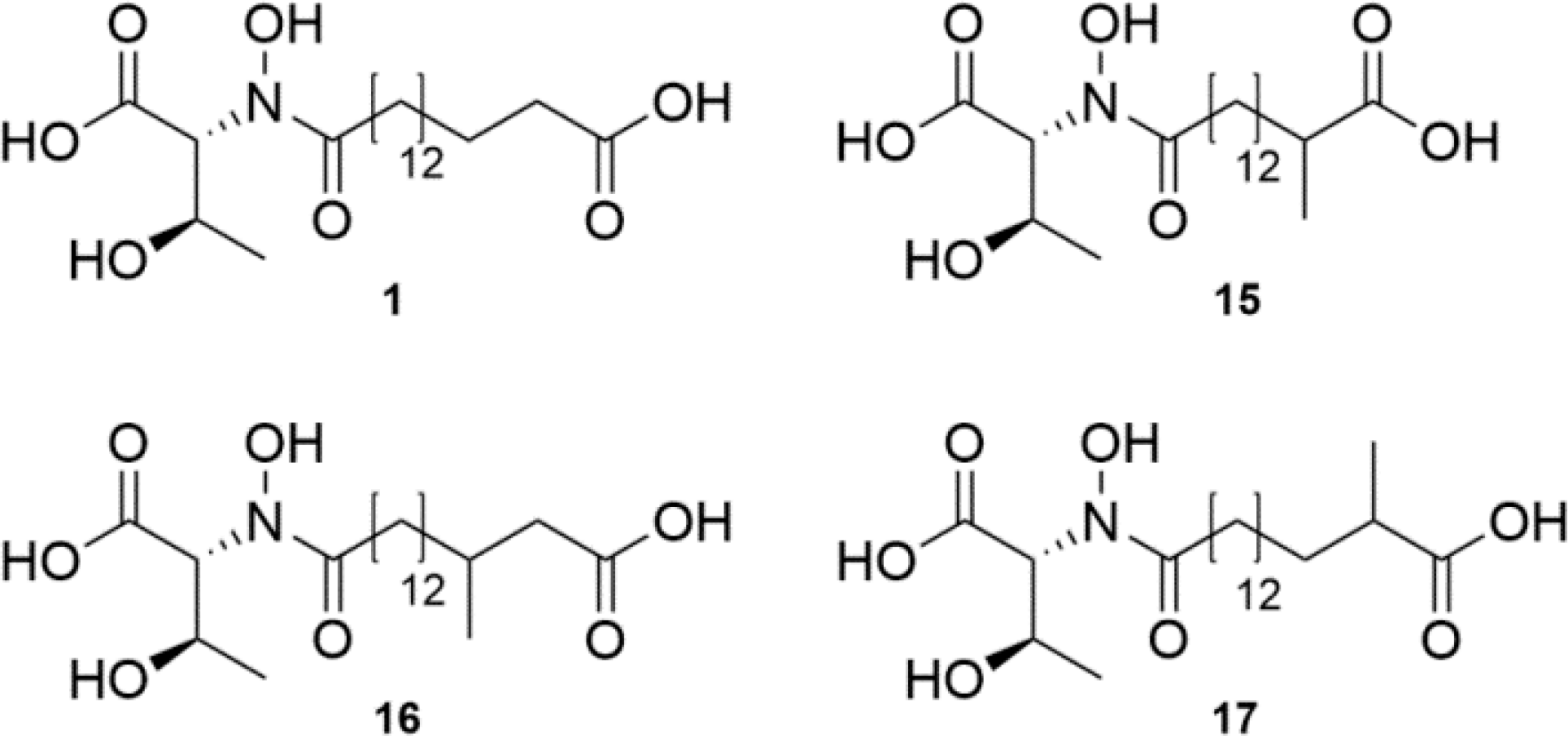
Lipothrenin A (**1**) and derivatives **15**–**17** produced by the deletion mutant I6_ΔlitB.

The genes *litB*, *litD*, *litF*, *litG*, and *litJ* are related to fatty acid synthesis, recruitment, and activation. De novo fatty acid synthesis in *Streptomyces* is catalyzed by type II fatty acid synthase. In most *Streptomyces* species, the core genes *fabD* (malonyl acyltransferase), *fabH* (keto synthase III), *acpP* (acyl carrier protein), and *fabF* (keto synthase II) are clustered, while enoylreductase, ketoreductase, and dehydratase genes are located elsewhere in the genome.^30−32^ BLAST analysis of LitB revealed similarities to ketosynthase FabH from *Streptomyces*. FabH uses acetyl-CoA, butyryl-CoA, and isobutyryl-CoA as starter units to catalyze the biosynthesis of linear and branched-chain fatty acids.^33,34^ The exclusive appearance of linear hexadecanedioic acid in lipothrenin **1**–**4** indicated that *litB* might be involved in biosynthesis of lipothrenin exclusively using acetyl-CoA. This hypothesis was confirmed by deletion mutant I6_ΔlitB, which showed an altered metabolic profile that included compounds with *m*/*z* 418.1 Da (**16**, **17**) and *m*/*z* 404.1 Da (**15**) (Figure S2). Isolation of the new compounds **15**–**17** and structure elucidation by NMR spectroscopy revealed lipothrenin A derivatives carrying branched-chain dicarboxylic fatty acid moieties (Figure 5, Figures S6–S8, S76–S92, Tables S4–S6). LitB may therefore be complemented by the native fabH gene encoded in the genome of the heterologous host *S. albus* Δ14. If present in the host genome, LitB seems to outperform the native KS genes, guiding the formation of linear hexadecanedioic acid.

Further deletions of fatty acid biosynthesis- and activation-related genes within the *lit*-cluster, including *ΔlitJ*, did not result in fatty acid chain modifications. The dicarboxylic fatty acid moieties may therefore be mainly generated by fatty acid synthase during primary metabolism. Fatty acid synthesis is very active during the bacterial log phase since fatty acids are needed for bacterial cell wall formation. However, in the stationary phase, fatty acid production is shut down almost completely; hence, another system to supply dicarboxylic fatty acids for lipothrenin production is needed. The *lit*-cluster contains several of these genes that may supply and activate dicarboxylic fatty acids. LitG shows sequence similarity to lipases and might be involved in fatty acid recruitment, while the subsequent activation is performed by LitD and LitF, representing fatty-acyl AMP ligase (FAAL) and acyl carrier protein (ACP). Similar activation mechanisms have been found in the lipidation reaction during daptomycin biosynthesis and in the biosynthesis of the recently described long-chain acyl phenols.^35,36^ Deletion of *litG* and *litD* resulted in a significant reduction but not in the abolishment of **1**–**14** produced by mutants I6_ΔlitG and I6_ΔlitD (Figure 4, Figure S2). The activation of hexadecanedioic acid may therefore also occur by native genes from the primary metabolism; however, lipothrenin production might benefit from higher expression levels of *litD* and *litF* and the efficiency of LitD and LitF that is specific for hexadecanedioic acid.

The biosynthesis of hydroxamate moieties in siderophores usually occurs via monooxygenation of the free amino group of ornithine, lysine, or *N*-hydroxydiaminoalkane, followed by *N*-acylation or intramolecular cyclization of the *N*-hydroxylated precursor.^37−40^ In contrast, *N*-hydroxylated amino acid moieties such as N-OH-threonine from lipothrenins **1**, **5**, and **6** are uncommon among hydroxamate siderophores. Previous studies have demonstrated that biosynthetic *N*-hydroxylation of amino acids is catalyzed by cytochrome P450 enzymes, FAD-dependent oxidoreductases, and nonheme iron-dependent enzymes.^41−45^ Analysis of the lipothrenin BGC revealed a diiron oxygenase LitN showing sequence similarity to 4-aminobenzoate *N*-oxygenase AurF, a nonheme dinuclear iron monooxygenase that catalyzes conversion of aryl-amine substrates to aryl-nitro products during aureothin biosynthesis.^46,47^ LitN is followed by the DUF4873-domain-containing protein LitO, which is often associated with flavin-binding monooxygenases. Analysis of the metabolic profiles of deletion mutants I6_ΔlitN and I6_ΔlitO showed the production of deoxy-isomers **2**, **3**, and **7**–**10** Simultaneously, the production of the *N*-hydroxylated compounds **1**, **5**, and **6** was reduced in I6_ΔlitO and abolished in I6_ΔlitN (Figure 4, Figure S3). High production of the deoxylipothrenin B derivatives suggests that hydroxylation occurs on the amide nitrogen rather than on the free amine, as seen in the biosynthesis of other hydroxamates. This mechanism may represent a previously undescribed biosynthetic pathway of the hydroxamate moiety. Although I6_ΔlitO showed alterations in the metabolic profile, the exact role of the DUF4873-domain-containing protein LitO in the hydroxylation process is unclear at that point.^20^

The previous stereochemical assignment of lipothrenin A (**1**) revealed that *N*-oxygenation occurs selectively at the d-*allo*-Thr-containing lipothrenin B derivatives (**2**, **7**, and **9**), indicating that *litN* acts stereospecifically. The presence of both the l-Thr and d-*allo*-Thr moieties in the lipothrenin B group suggests the presence of an enzyme encoded in the *lit*-BGC that performs l-Thr epimerization. However, subsequent analysis of the encoded proteins failed to identify any epimerases. The gene involved in the l-Thr stereoconversion was revealed during the gene deletion experiments. The metabolic profile of mutant strain I6_ΔlitM showed exclusive production of *m*/*z* 388 [M + H]^+^, while no other lipothrenin derivatives were detected. Isolation of the compound and analysis of the ^1^H NMR spectra revealed that only l-lipothrenin B (**3**) was produced (Figures S93–S95). Since genes *litK–litO* are on the same operon, replacing *litM* with a resistance cassette will prevent translation of genes *litN* and *litO.* Therefore, the cassette was removed by restriction digestion in vitro using the MssI restriction sites that are contained in the cassette. Subsequent ligation and introduction of the modified BAC into *S. albus* Δ14 via conjugation led to mutant strain I6_ΔlitM without ampicillin resistance, namely, I6_ΔlitMcut. The MssI sites are designed to allow in-frame translation of the downstream genes *litN* and *litO*, enabling *N*-hydroxylation of lipothrenins. However, comparison of the metabolic profiles of mutants I6_ΔlitM and I6_ΔlitMcut did not show any alteration, confirming the hypothesis that only d-*allo*-Thr-containing lipothrenins are hydroxylated. Thus, LitM seems to change the configuration of l-Thr to d-*allo*-Thr. The protein shows sequence similarity to that of the ThiF family adenylyltransferase and tRNA threonylcarbamoyladenosine dehydratase (TcdA). A dehydratase function would suggest a dehydration mechanism which could promote a configuration change at the CH-2, leading to d-*allo*-Thr. This hypothesis is supported by studies of the dehydratase domains from modular polyketide synthases that catalyze intrinsic *syn* dehydration, while no dehydration was observed for the diastereomeric substrates.^48^ A similar mechanism has been described for rat liver peroxisomal d-3-hydroxyacyl-CoA dehydratase acting on fatty acids.^49^ Dehydro-lipothrenins **4** and **11**–**14** might appear as a side product of the dehydration reaction, strongly supporting the epimerization activity of LitM.

The absence of *N*-acetylcysteine-lipothrenin derivatives in the production profile of I6_ΔlitM could be related to the lack of stereoconversion of l-lipothrenin B (**3**). The main mechanism in *Streptomyces* to remove toxic compounds from the cell involves mycothiol.^50^d-Lipothrenin (**2**) might trigger the detoxification mechanism, while l-lipothrenin B (**3**) does not cause any harm to the cell; hence, *N*-acetylcysteine-lipothrenins are not produced by I6_ΔlitM. The *N*-acyl-cysteine addition in the α-position is unique and has not been described for fatty acid natural products. Usually *N*-acetylcysteine modification is performed via a Michael addition of mycothiol (MSH) onto the α/β-unsaturated carbonyl. Here, it seems likely that *N*-acetylcysteine-lipothrenins are generated via a nucleophilic attack of the α-carbon of the lipothrenin enolate at the disulfide bond of the reduced mycothiol dimer (MSSM), while MSH acts as a leaving group. However, this putative mechanism leading to *N*-acetylcysteine-modified natural products is chemically challenging, and it might require catalysis by an unknown enzyme.

**Figure 6:**
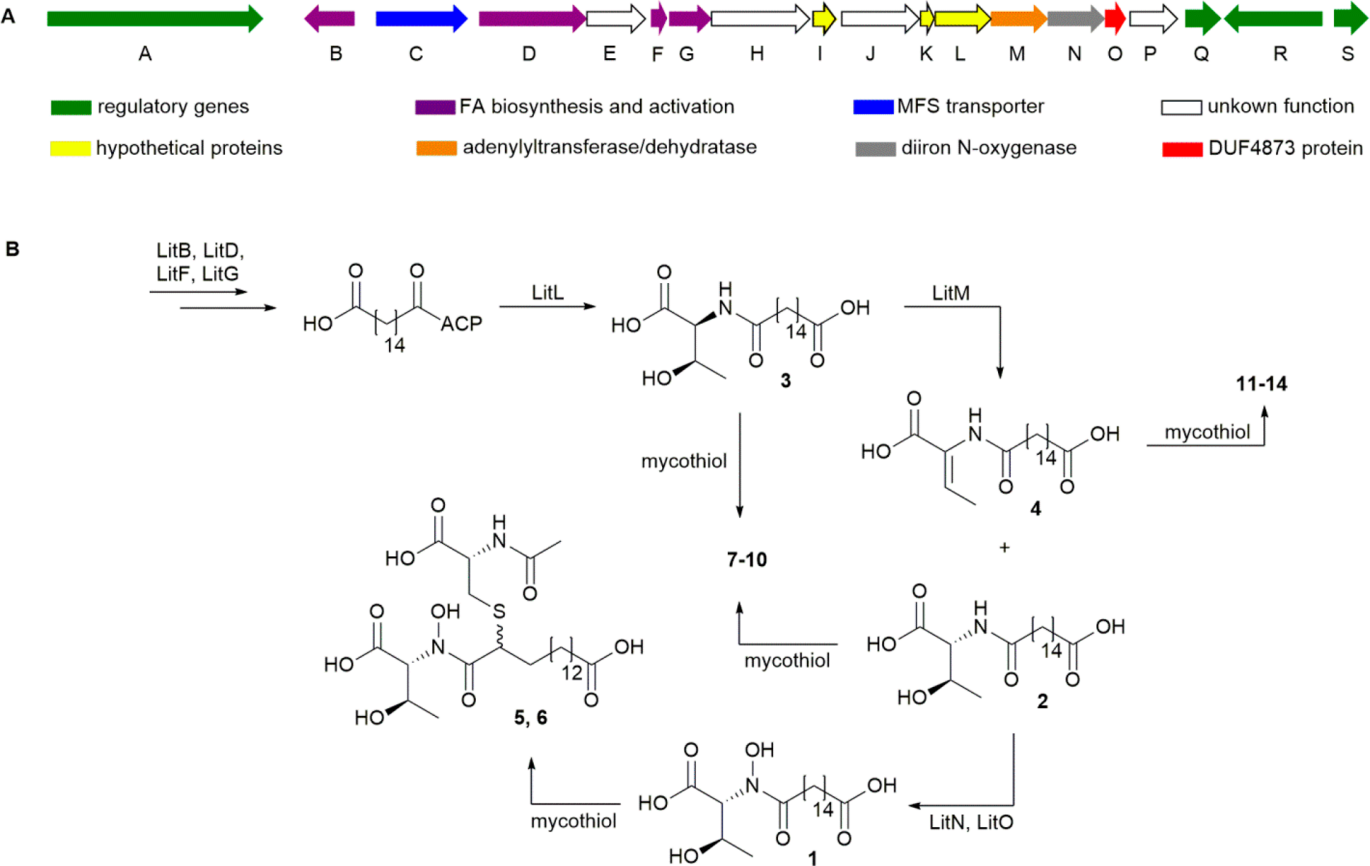
Lipothrenin biosynthetic gene cluster (A) and the proposed biosynthetic pathway of lipothrenins (B).

Genes with an effect on the peptide bond formation between l-Thr and activated hexadecanedioic acid were not unambiguously identified during the deletion experiments. Deletions of genes *litE*, *litH*, *litJ*, and *litP* showed only minor effects on lipothrenin production, not taking over vital functions in the lipothrenin biosynthesis process. Deletion of *litL* led to an abolishment of the production of lipothrenins **1**–**14** (Figure 4, Figure S3). Therefore, LitL might have a function in lipothrenin biosynthesis, e.g., the peptide bond formation leading to l-lipothrenin B (**3**). However, this vague hypothesis requires further experimentation that will not be addressed in this work. The results of most of the gene deletion experiments provided good insight into the individual steps of lipothrenin biosynthesis and, in combination, lead to a proposed biosynthetic pathway (Figure 6).

**Figure 7:**
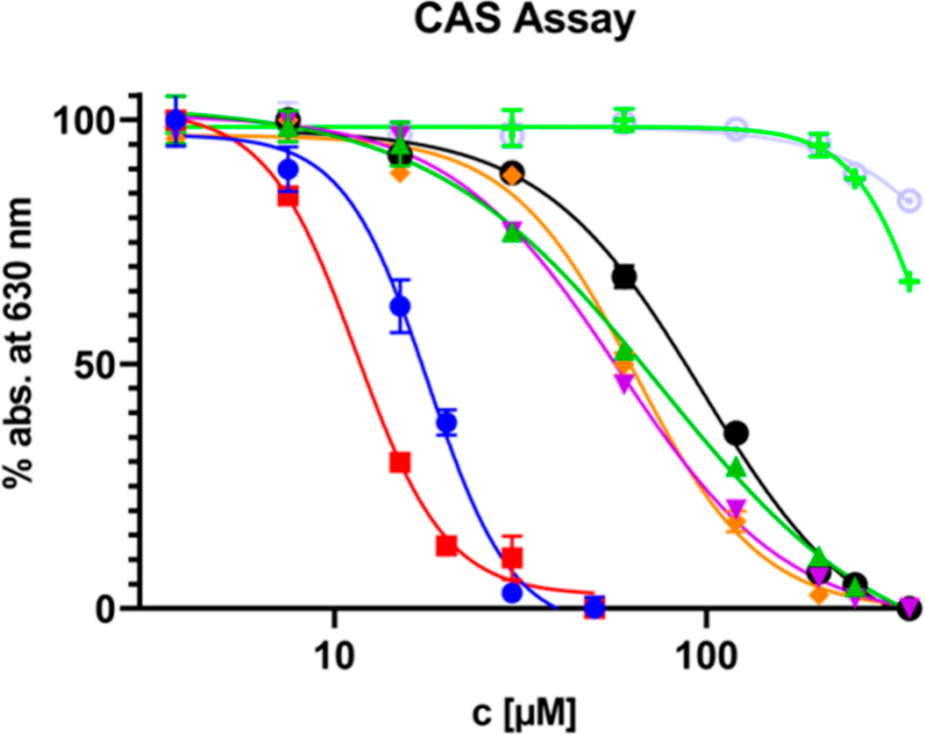
CAS assay determination of the EC_50_ values. EC_50_ is the concentration where a 50% reduction of the absorption at 630 nm occurs.

The hydroxamate moieties in lipothrenin A (**1**) and *N*-acetylcysteine-lipothrenins A and A1 (**5**, **6**) indicate iron-chelating properties. This was confirmed with the liquid chrome azurol S (CAS) assay, which revealed EC_50_ values approximately 5-fold higher than those of deferoxamine (DFOA), while no chelating properties were observed for desoxylipothrenins **2**/**3** and **7** (Table 6, Figure 7). Iso-lipothrenin A (**15**) showed a slightly reduced activity, indicating that the ω-carboxylate moiety might be involved in Fe binding. Lipothrenins were tested against several bacterial strains, including *S. aureus* Newman, *E. coli* BW25113 (wt), *Staphylococcus carnosus* DSM-20501, *B. subtilis* DSM-10, *Kocuria rhizophila* DSM-348, *Enterococcus mundtii* DSM-4840, *Pseudomonas putida*, *Micrococcus luteus* DSM-20030, and *Erwinia perscina* DSM-19328. However, in the range 0.03–64 μg·mL^–1^, no antibiotic activity was observed for any of the strains. Furthermore, no cytotoxic activity was recorded against Hep G2 (human liver cancer) cells.

**Table 6:** EC_50_ Values of Selected Lipothrenins Determined by the CAS Assay Method

compound	EC_50_^a^
EDTA	18.0 ± 1.3
DFOA	11.0 ± 0.8
15	75.0 ± 9.9
**1**	58.0 ± 3.4
**5**	64.0 ± 4.6
**6**	97.0 ± 7.8
**2**, **3**	
**7**	

a EC_50_ = concentration at 50% reduction of absorption at 630 nm.

In summary, using a combined approach of dereplication, genome mining, and heterologous expression, the structure and biosynthesis of a new hydroxamate moiety containing lipo-amino acids named lipothrenins were discovered. Gene deletion experiments revealed an interesting biosynthetic pathway with many new genes that are involved in the *N*-oxygenation, isomerization, and amide bond formation during lipothrenin biosynthesis. Studying their mechanism and function in detail could provide new enzyme catalysts that can be used in late-stage modifications during natural product total synthesis or as a source of genes for combinatorial biosynthesis. The results from this work demonstrated that *Streptomyces* are still a valuable source for new chemical scaffolds and should be further investigated.

## Experimental Section

### General Experimental Procedures

Optical rotations were measured on a PerkinElmer model 241 (Überlingen, Germany). 1D and 2D NMR spectra were recorded on a Bruker Avance I 500 MHz (Bruker, BioSpin GmbH, Rheinstetten, Germany) equipped with a 5 mm BBO probe at 298 K. Edited-HSQC, HSQC-TOCSY, HMBC, ^1^H–^1^H COSY, ROESY, NOESY, and N-HSQC spectra were recorded using the standard pulse programs from TOPSPIN v.2.1 software. The chemical shifts (δ) were reported in parts per million (ppm) relative to TMS. Deuterated DMSO-*d*_6_ (δH 2.50 ppm, δC 39.51 ppm) from Deutero (Kastellaun, Germany) was used as the solvent. HRESIMS was obtained from an LTQ Orbitrap XL mass spectrometer (ThermoFisher Scientific, Waltham, MA, USA) and a maXis high-resolution LC-QTOF system (Bruker, Billerica, MA, USA). LRESIMS was obtained by amaZon speed (Bruker). Compound separation prior to MS was performed by a Dionex Ultimate 3000 UPLC system (Thermo Fisher Scientific) equipped with an ACQUITY UPLC BEH C_18_ 1.7 μm column (30, 50, or 100 mm, Waters Corporation, Milford, MA, USA) coupled to a diode array (PDA) detector. Compound isolation was done by an Isolera One flash purification system equipped with a Chromabond RS330 C_18_ ec column (Macherey-Nagel, Düren, Germany), followed by Sephadex-LH20 column chromatography (CC). Preparative purification steps were carried out on a Waters Autopurification system (Waters Corporation) with an SQD2-MS-detector and equipped with a preparative VP 250/21 Nucleodur C_18_ HTec 5 μm column (Machery Nagel, Düren, Germany) using a flow rate of 20 mL/min at room temperature and semipreparative purification on an Agilent Infinity 1100 and 1260 series reversed phase (RP) HPLC system equipped with a SynergiTM 4 μm Fusion-RP C_18_ 80 Å 250 mm × 10 mm column (Phenomenex, Torrance, CA, USA) using a flow rate of 4 mL/min at 45 °C.

### BACs, Strains, and Media

All strains and bacterial artificial chromosomes used in this work are given in Table S1. *Escherichia coli* strains were cultured in LB medium.^51^*Streptomyces* strains were grown on soya flour mannitol agar (MS agar)^52^ or in liquid tryptic soy broth (TSB; Sigma–Aldrich, St. Louis, MO, USA) for cultivation. Liquid DNPM medium (40 g/L dextrin, 7.5 g/L soytone, 5 g/L baking yeast, and 21 g/L MOPS, pH 6.8) was used for secondary metabolite expression. The antibiotics kanamycin, apramycin, ampicillin, and nalidixic acid were supplemented as needed (Carl Roth GmbH, Karlsruhe, Germany).

### General Bacterial Metabolite Extraction and Dereplication Procedure

To identify metabolites and dereplication, strains were grown in TSB medium for 24 h. Subsequently, 1 mL of the precultured strains was inoculated in DNPM production medium and grown for 6 days at 28 °C and 180 rpm. Metabolites were extracted from the supernatant with *n*-butanol. Bacterial extracts were analyzed by RP-C_18_ HPLC-HRESIMS using a linear gradient of 5–95 vol % aqueous acetonitrile (ACN) with 0.1% formic acid at a flow rate of 0.6 mL/min and a column oven temperature of 45 °C. Data analysis was performed using Compass Data Analysis v. 4.1 (Bruker) and Xcalibur v. 3.0 (ThermoFisher Scientific) software. High-resolution masses were compared to those of Dictionary of Natural Products entities to identify unknown metabolites.

### Large-Scale Cultivation, Extraction, and Isolation

*S. aureus* LU18118 and *S. albus* I6_ΔliB were each grown in 10 L and *S. albus* I6_ΔliM was grown in 5 L of DNPM and extracted with *n*-butanol. The dry crude extracts were dissolved in 100 mL of methanol. During isolation and purification fractions were monitored by HPLC-LRESIMS. Extracts were purified using flash chromatography and a gradient of 5–35 vol % aqueous methanol for 1 column volume (CV) followed by 35–80 vol % aqueous methanol for 7 CV at a flow rate of 100 mL/min and UV detection at 210 and 280 nm. *S. aureus* LU18118 yielded four fractions (F1–F4) containing 390–1231 mg of extract. *S. albus* I6_ΔliB and *S. albus* I6_ΔlitM both yielded one fraction with compounds **15**–**17** (I6_ΔliB) and compound **3** (I6_ΔlitM), respectively.

F1 was purified by Sephadex LH-20 CC, leading to two fractions (S1.1 and S1.2). Fraction S1.1 was further purified by semipreparative RP-C_18_ HPLC using gradient elution (ACN–H_2_O + 0.1% formic acid, 43:57–45:55), yielding compounds **7** (5.3 mg), **8** (5.8 mg), and **9**/**10 (**13 mg**)**. Fraction S1.2 was purified by RP-C_18_ HPLC using isocratic conditions (ACN–H_2_O + 0.1% formic acid, 50/:50), yielding compounds **5** (16.6 mg), **6** (13.9 mg), **11**/**12** (6.5 mg), and **13**/**14** (4.0 mg).

F2 was purified by Sephadex LH-20 CC, and fractions containing compound **2**/**3** were submitted to preparative RP-C_18_ HPLC using gradient elution (MeOH–H_2_O + 0.1% formic acid, 87/:13–90:10), yielding pure compound **2**/**3** (11.7 mg).

F3 was purified by Sephadex LH-20 CC, and fractions containing compound **1** were submitted to semipreparative RP-C_18_ HPLC using gradient elution (ACN–H_2_O + 0.1% formic acid, 57/:43–65:35), yielding pure compound **1** (3.0 mg).

F4 was purified by Sephadex LH-20 CC, and fractions containing compound **4** were submitted to semipreparative RP-C_18_ HPLC using isocratic conditions (ACN–H_2_O + 0.1% formic acid, 57/:43), yielding pure compound **4** (2.0 mg).

The *S. albus* I6_ΔliB flash fraction was further purified with Sephadex LH-20 CC and preparative RP-C_18_ HPLC using gradient elution (ACN–H_2_O+ 0.1% formic acid, 50:50–75:25), yielding compounds **15** (5.0 mg), **16** (19.1 mg), and **17** (31.2 mg).

The *S. albus* I6_ΔliM flash fraction was further purified with Sephadex LH-20 CC and preparative RP-C_18_ HPLC using gradient elution (ACN–H_2_O + 0.1% formic acid, 40:60–85:15), yielding compounds **3** (39 mg).

#### Lipothrenin A (**1**)

White powder; 3.0 mg; [α]_D_^20^ +7.5 (*c* 0.24, MeOH); UV (ACN/H_2_O) λ = 208 nm; ^1^H and ^13^C NMR data, see Table 1; ESI-TOF-MS *m*/*z* 404.2644 [M + H]^+^ (calcd for C_20_H_38_NO_7_ 404.2648), see Figure S9.

#### Lipothrenin B (**2**, **3**)

White powder; 11.7 mg; UV (ACN–H_2_O) λ = 208 nm; ^1^H and ^13^C NMR data, see Table 1; ESI-TOF-MS *m*/*z* 388.2696 [M + H]^+^ (calcd for C_20_H_38_NO_6_ 388.2699), see Figure S15..

#### Z-Lipothrenin C (**4**)

White powder; 2.0 mg; UV (ACN–H_2_O) λ = 208 nm; ^1^H and ^13^C NMR data, see Table 1; ESI-TOF-MS *m*/*z* 370.2589 [M + H]^+^ (calcd for C_20_H_36_NO_5_ 370.2593), see Figure S22.

#### 2-*N*-Acetylcysteine-d-lipothrenin A (**5**)

White powder; 16.6 mg; [α]_D_^20^ +18.1 (*c* 1.92, MeOH); UV (ACN–H_2_O) λ = 208 nm; ^1^H and ^13^C NMR data, see Tables 2 and 3; ESI-TOF-MS *m*/*z* 565.2798 [M + H]^+^ (calcd for C_25_H_45_N_2_O_10_S 565.2795), see Figure S29.

#### 2-*N*-Acetylcysteine-d-lipothrenin A_1_ (**6**)

White powder; 13.9 mg; [α]_D_^20^ −0.84 (*c* 1.55, MeOH); UV (ACN–H_2_O) λ = 208 nm; ^1^H and ^13^C NMR data, see Tables 2 and 3; ESI-TOF-MS *m*/*z* 565.2786 [M + H]^+^ (calcd for C_25_H_45_N_2_O_10_S 565.2795), see Figure S35.

#### 2-*N*-Acetylcysteine-d-lipothrenin B (**7**)

White powder; 5.3 mg; [α]_D_^20^ +14.34 (*c* 0.45, MeOH); UV (ACN–H_2_O) λ = 208 nm; ^1^H and ^13^C NMR data, see Tables 2 and 3; ESI-TOF-MS *m*/*z* 549.2846 [M + H]^+^ (calcd for C_25_H_45_N_2_O_9_S 549.2846), see Figure S42.

#### 2-*N*-Acetylcysteine-l-lipothrenin B (**8**)

White powder; 5.8 mg; [α]_D_^20^ +22.71 (*c* 0.57, MeOH); UV (ACN–H_2_O) λ = 208 nm; ^1^H and ^13^C NMR data, see Tables 2 and 3; ESI-TOF-MS *m*/*z* 549.2853 [M + H]^+^ (calcd for C_25_H_45_N_2_O_9_S 549.2846), see Figure S49.

#### 2-*N*-Acetylcysteine-d/l-lipothrenin B1 Mixture (**9**, **10**)

White powder; 13.0 mg; UV (ACN–H_2_O) λ = 208 nm; ^1^H and ^13^C NMR data, see Tables 2 and 3; ESI-TOF-MS *m*/*z* 549.2851 [M + H]^+^ (calcd for C_25_H_45_N_2_O_9_S 549.2846), see Figure S56.

#### 2-*N*-Acetylcysteine-Z-lipothrenin C and C_1_ Mixture (**11**, **12**)

White powder; 6.5 mg; UV (ACN–H_2_O) λ = 208 nm; ^1^H and ^13^C NMR data, see Table 4; ESI-TOF-MS *m*/*z* 531.2742 [M + H]^+^ (calcd for C_25_H_43_N_2_O_8_S 531.2740), see Figure S63.

#### 2-*N*-Acetylcysteine-*E*-lipothrenin C and C_1_ Mixture (**13**, **14**)

White powder; 4.0 mg; UV (ACN–H_2_O) λ = 208 nm; ^1^H and ^13^C NMR data, see Table 4; ESI-TOF-MS *m*/*z* 531.2737 [M + H]^+^ (calcd C_25_H_43_N_2_O_8_S 531.2740), see Figure S70.

#### iso-Lipothrenin A (**15**)

White powder; 5.0 mg; [α]_D_^20^ +7.5 (*c* 0.22, MeOH); UV (ACN–H_2_O) λ = 208 nm; ^1^H and ^13^C NMR data, see Table S4; ESI-TOF-MS *m*/*z* 404.2648 [M + H]^+^ (calcd for C_20_H_38_NO_7_ 404.2648), see Figure S76.

#### 14-Methyl-lipothrenin A (**16**)

White powder; 19.1 mg; [α]_D_^20^ +7.3 (*c* 1.71, MeOH); UV (ACN–H_2_O) λ = 208 nm; ^1^H and ^13^C NMR data, see Table S5; ESI-TOF-MS *m*/*z* 418.2813 [M + H]^+^ (calcd for C_21_H_40_NO_7_ 418.2805), see Figure S81.

#### 15-Methyl-lipothrenin A (**17**)

White powder; 31.2 mg; [α]_D_^20^ +2.0 (*c* 2.92, MeOH); UV λ = 208 nm; ^1^H and ^13^C NMR data, see Table S6; ESI-TOF-MS *m*/*z* 418.280 [M + H]^+^ (calcd for C_21_H_40_NO_7_ 418.285), see Figure S87.

### Marfey’s Method

Lipothrenins were hydrolyzed in 100 μL of 6 N HCl at 110 °C for 1 h. While cooling down, the sample was dried for 15 min under nitrogen and dissolved in 110 mL of water, and 50 μL each was transferred into 1.5 mL Eppendorf tubes. To the hydrolysate were added 20 μL of 1 N NaHCO_3_ and 20 μL of 1% l-FDLA (*N*^α^-(5-fluoro-2,4-dinitrophenyl)-l-leucinamide) or d-FDLA in acetone, respectively. The amino acid standards were prepared the same way only using l-FDLA. The reaction mixtures were incubated at 40 °C for 90 min at 700 rpm and subsequently quenched with 2 N HCl to stop the reaction. The samples were diluted with 300 μL of ACN, and 1 μL was analyzed by a MaXis high-resolution LC-QTOF system using aqueous ACN with 0.1 vol % formic acid and an adjusted gradient of 5–10 vol % in 2 min, 10–25 vol % in 13 min, 25–50 vol % in 7 min, and 50–95 vol % in 2 min. Sample detection was carried out at 340 nm.

### Isolation and Manipulation of DNA

BAC extraction from an *S. aureus* LU18118 constructed genomic library (Intact Genomics, USA), DNA manipulation, *E. coli* transformation, and *E. coli*/*Streptomyces* intergeneric conjugation were performed according to standard protocols.^51−53^ Plasmid DNA was purified with a BACMAX DNA purification kit (Lucigen, Middleton, WI, USA). Restriction endonucleases were used according to the manufacturer’s recommendations (New England Biolabs, Ipswich, MA, USA). BAC I6 derivatives with gene deletions were constructed using the Red/ET approach. For this, the ampicillin marker from pUC19 was amplified by PCR with primers harboring overhang regions complementary to the boundaries of the DNA to be deleted. Recombineering of the BAC was performed with amplified fragments. The recombinant BACs were analyzed by restriction analysis or PCR. The primers used for recombineering purposes are listed in Table S2. Primers for PCR to determine the correct mutants are listed in Table S3.

### CAS Assay

Iron binding affinity of lipothrenins was determined by the chrome-azurol S assay method as described in the literature.^54^ A 96-well plate was prepared containing lipothrenins dissolved in 100 μL of DMSO to obtain a 0, 3.75, 7.5, 15, 30, 60, 120, 200, 250, and 350 μM concentration in each well. As a reference, DFOA and EDTA were prepared in the same way with concentrations of 0, 3.75, 7.5, 15, 20, and 30 μM in each well. After adding 100 μL of freshly prepared CAS assay solution, UV–vis absorbance was measured at the POLARstar OMEGA microplate reader (BMG Labtech) at 630 nm every 5 min for 60 min in total. A reduction of the absorbance of the CAS solution by 50% at 630 nm was used to determine EC_50_ values. Calculation of EC_50_ values was performed by GraphPad Prism 9.3.1 software.

## Supporting Information Available

Strains, plasmids, BACs, and primers used in this study, LC-MS spectra of *S. albus* I6_Δ strains, LC–MS spectra of Marfey’s method, HRESIMS, NMR data of compounds **15**–**17**, 1D and 2D NMR spectra of all compounds (PDF)
