# Supplementary material for: Heterologous Production and Biosynthesis of Threonine-16:0dioic acids with a Hydroxamate Moiety
Source: J Nat Prod. 2023 Sep 20;86(10):2258–69. doi: 10.1021/acs.jnatprod.3c00097 (PMC10616846; doi:10.1021/acs.jnatprod.3c00097)
Supplement: Supplementary file 1 — np3c00097_si_001.pdf [file np3c00097_si_001.pdf]

Supplementary Information

Heterologous Production and Biosynthesis of Threonine-16:0dioic acids with a Hydroxamate Moiety

Marc Stierhof,[†] *Maksym Myronovskyi,* [†] *Josef Zapp,* [‡] *and Andriy Luzhetskyy,* [†§]

† Department of Pharmaceutical Biotechnology, and

‡ Department of Pharmaceutical Biology, Saarland University, 66123 Saarbruecken, Germany

§ Helmholtz Institute for Pharmaceutical Research Saarland

.

Table of Contents

Heterologous Expression and Genetic Manipulation 8

Chromatograms from Gene Deletion Experiments 13

Stereochemistry Analysis by Marfey's Method 16

NMR Spectroscopy Data 18

NMR Spectra 20

Supplementary Tables

Table S1: Strains, Plasmids and BACS used in this study 8

Table S2: Used primers for RedET. 9

Table S3: Used primers for PCR. 11

Table S4: NMR data (500 MHz, DMSO-*d6*) for *Iso*-lipothrenin A (15) 18

Table S5: NMR data (500 MHz, DMSO-*d6*) for 14-methyl-lipothrenin A (16) 18

Table S6: NMR data (500 MHz, DMSO-*d6*) for 15-methyl-lipothrenin A (17) 19

Supplementary Figures

Figure S1. Chromatogram showing the extracted masses from lipothrenin A (1, 4 and 5, red), lipothrenins B (2, 3 and 7-10, green) and lipothrenin C (4 and 11-14, blue) from butanol extract from deletion mutants covering the flanking regions of the *lit* BGC. 13

Figure S2. Chromatogram showing the extracted masses from lipothrenins A (1, 4 and 5, red) and lipothrenin A derivatives 15 – 17 (black), lipothrenins B (2, 3 and 7-10, green) and lipothrenins C (4 and 11-14, blue) from butanol extracts from deletion mutants covering I6_ΔlitB – litJ. 14

Figure S3. Chromatogram showing the extracted masses from lipothrenins A (1, 4 and 5, red), lipothrenins B (2, 3 and 7-10, green) and lipothrenins C (4 and 11-14, blue) from butanol extracts from deletion mutants covering I6_ΔlitL – litP. 15

Figure S4: LC-MS chromatograms showing the extracted mass of threonine of hydrolysed 1 (a), 2 and 3 (b) 7-10 (c-e) and the references L-, D-, L-*allo*- and D-a*llo*-threonine (f) derivatized with L-FDLA. 16

Figure S5: LC-MS chromatograms of the extracted masse of the DLA-cys-FA conjugate derived from hydrolysis of 2-NAC-DL-lipothrenin B/B_1 and derivatisation with L- and D-FDLA. 17

Figure S6. Structure of compound 15 with selected COSY (—) and HMBC (↷) correlations. 18

Figure S7. Structure of compound 16 with selected COSY (—) and HMBC (↷) correlations. 19

Figure S8. Structure of compound 17 with selected COSY (—) and HMBC (↷) correlations. 19

Figure S9. The HRESIMS of compound 1. 20

Figure S10. ^{1}H NMR spectrum (500 MHz, DMSO-d_6) of lipothrenin A (1). 20

Figure S11: ^{13}C NMR spectrum (500 MHz, DMSO-d_6) of lipothrenin A (1). 21

Figure S12: ^{1}H-^{1}H-COSY spectrum (500 MHz, DMSO-d_6) of lipothrenin A (1). 21

Figure S13: HSQC spectrum (500 MHz, DMSO-d_6) of lipothrenin A (1). 22

Figure S14: HMBC spectrum (500 MHz, DMSO-d_6) of lipothrenin A (1). 22

Figure S15. The HRESIMS of compound 2, 3. 23

Figure S16: ^{1}H NMR spectrum (500 MHz, DMSO-d_6) of D- and L-lipothrenin B (2, 3). 23

Figure S17. Zoomed in ^{1}H NMR spectrum (500 MHz, DMSO-d_6) of D- and L-lipothrenin B (2, 3). 24

Figure S18: ^{13}C NMR spectrum (500 MHz, DMSO-d_6) of D- and L-lipothrenin B (2, 3). 24

Figure S19: ^{1}H-^{1}H-COSY spectrum (500 MHz, DMSO-d_6) of D- and L-lipothrenin B (2, 3). 25

Figure S20: HSQC spectrum (500 MHz, DMSO-d_6) of D- and L-lipothrenin B (2, 3). 25

Figure S21: HMBC spectrum (500 MHz, DMSO-d_6) of D- and L-lipothrenin B (2, 3). 26

Figure S22. The HRESIMS of compound 4. 26

Figure S23: ^{1}H NMR spectrum (500 MHz, DMSO-d_6) of lipothrenin C (4). 27

Figure S24: ^{13}C NMR spectrum (500 MHz, DMSO-d_6) of lipothrenin C (4). 27

Figure S25: ^{1}H-^{1}H-COSY spectrum (500 MHz, DMSO-d_6) of lipothrenin C (4). 28

Figure S26: HSQC spectrum (500 MHz, DMSO-d_6) of lipothrenin C (4). 28

Figure S27: HMBC spectrum (500 MHz, DMSO-d_6) of lipothrenin C (4). 29

Figure S28: ROESY spectrum (500 MHz, DMSO-d_6) of lipothrenin C (4). 29

Figure S29. The HRESIMS of compound 5. 30

Figure S30: ^{1}H NMR spectrum (500 MHz, DMSO-d_6) of 2-NAC-lipothrenin A (5). 30

Figure S31. Zoomed in ^{1}H NMR spectrum (500 MHz, DMSO-d_6) of 2-NAC-lipothrenin A (5). 31

Figure S32: ^{13}C NMR spectrum (500 MHz, DMSO-d_6) of 2-NAC-lipothrenin A (5). 31

Figure S33: ^{1}H-^{1}H-COSY spectrum (500 MHz, DMSO-d_6) of 2-NAC-lipothrenin A (5). 32

Figure S34: HMBC spectrum (500 MHz, DMSO-d_6) of 2-NAC-lipothrenin A (5). 32

Figure S35. The HRESIMS of compound 6. 33

Figure S36: ^{1}H NMR spectrum (500 MHz, DMSO-d_6) of 2-NAC-lipothrenin A_1 (6). 33

Figure S37. Zoomed in ^{1}H NMR spectrum (500 MHz, DMSO-d_6) of 2-NAC-lipothrenin A_1 (6). 34

Figure S38: ^{13}C NMR spectrum (500 MHz, DMSO-d_6) of 2-NAC-lipothrenin A_1 (6). 34
Figure S39: ^{1}H-^{1}H-COSY spectrum (500 MHz, DMSO-d_6) of 2-NAC-lipothrenin A_1 (6). 35
Figure S40: HSQC spectrum (500 MHz, DMSO-d_6) of 2-NAC-lipothrenin A_1 (6). 35
Figure S41: HMBC spectrum (500 MHz, DMSO-d_6) of 2-NAC-lipothrenin A_1 (6). 36
Figure S42. The HRESIMS of compound 7. 36
Figure S43: ^{1}H NMR spectrum (500 MHz, DMSO-d_6) of 2-NAC-D-lipothrenin B (7). 37
Figure S44. Zoomed in ^{1}H NMR spectrum (500 MHz, DMSO-d_6) of 2-NAC-D-lipothrenin B (7). 37
Figure S45: ^{1}H-^{1}H-COSY spectrum (500 MHz, DMSO-d_6) of 2-NAC-D-lipothrenin B (7). 38
Figure S46: HSQC spectrum (500 MHz, DMSO-d_6) of 2-NAC-D-lipothrenin B (7). 38
Figure S47: HMBC spectrum (500 MHz, DMSO-d_6) of 2-NAC-D-lipothrenin B (7). 39
Figure S48: ROESY spectrum (500 MHz, DMSO-d_6) of 2-NAC-D-lipothrenin B (7). 39
Figure S49. The HRESIMS of compound 8. 40
Figure S50: ^{1}H NMR spectrum (500 MHz, DMSO-d_6) of 2-NAC-L-lipothrenin B (8). 40
Figure S51. Zoomed in ^{1}H NMR spectrum (500 MHz, DMSO-d_6) of 2-NAC-L-lipothrenin B (8). 41
Figure S52: ^{1}H-^{1}H-COSY spectrum (500 MHz, DMSO-d_6) of 2-NAC-L-lipothrenin B (8). 41
Figure S53: HSQC spectrum (500 MHz, DMSO-d_6) of 2-NAC-L-lipothrenin B (8). 42
Figure S54: HMBC spectrum (500 MHz, DMSO-d_6) of 2-NAC-L-lipothrenin B (8). 42
Figure S55: ROESY spectrum (500 MHz, DMSO-d_6) of 2-NAC-L-lipothrenin B (8). 43
Figure S56. The HRESIMS of compound 9, 10. 43
Figure S57: ^{1}H NMR spectrum (500 MHz, DMSO-d_6) of 2-NAC-D-/L-lipothrenin B_1 mixture (9, 10). 44
Figure S58. Zoomed in ^{1}H NMR spectrum (500 MHz, DMSO-d_6) of 2-NAC-D-/L-lipothrenin B_1 mixture (9, 10). 44
Figure S59: ^{13}C NMR spectrum (500 MHz, DMSO-d_6) of 2-NAC-D-/L-lipothrenin B_1 mixture (9, 10). 45
Figure S60: ^{1}H-^{1}H-COSY spectrum (500 MHz, DMSO-d_6) of 2-NAC-D-/L-lipothrenin B_1 mixture (9, 10). 45
Figure S61: HSQC spectrum (500 MHz, DMSO-d_6) of 2-NAC-D-/L-lipothrenin B_1 mixture (9, 10). 46
Figure S62: HMBC spectrum (500 MHz, DMSO-d_6) of 2-NAC-D-/L-lipothrenin B_1 mixture (9, 10). 46
Figure S63. The HRESIMS of compound 11/12. 47
Figure S64: ^{1}H NMR spectrum (500 MHz, DMSO-d_6) of 2-NAC-*Z*-lipothrenin C and C_1 mixture (11, 12). 47

Figure S65. Zoomed in ^{1}H NMR spectrum (500 MHz, DMSO-d_6) of 2-NAC-*Z*-lipothrenin C and C_1 mixture (11, 12). 48
Figure S66: ^{13}C NMR spectrum (500 MHz, DMSO-d_6) of 2-NAC-*Z*-lipothrenin C and C_1 mixture (11, 12). 48
Figure S67: ^{1}H-^{1}H-COSY spectrum (500 MHz, DMSO-d_6) of 2-NAC-*Z*-lipothrenin C and C_1 mixture (11, 12). 49
Figure S68: HSQC spectrum (500 MHz, DMSO-d_6) of 2-NAC-*Z*-lipothrenin C and C_1 mixture (11, 12). 49
Figure S69: HMBC spectrum (500 MHz, DMSO-d_6) of 2-NAC-*Z*-lipothrenin C and C_1 mixture (11, 12). 50
Figure S70. The HRESIMS of compound 13/14. 50
Figure S71: ^{1}H NMR spectrum (500 MHz, DMSO-d_6) of 2-NAC-*E*-lipothrenin C and C_1 mixture (13, 14). 51
Figure S72. Zoomed in ^{1}H NMR spectrum (500 MHz, DMSO-d_6) of 2-NAC-*E*-lipothrenin C and C_1 mixture (13, 14). 51
Figure S73: ^{1}H-^{1}H-COSY spectrum (500 MHz, DMSO-d_6) of 2-NAC-*E*-lipothrenin C and C_1 mixture (13, 14). 52
Figure S74: HSQC spectrum (500 MHz, DMSO-d_6) of 2-NAC-*E*-lipothrenin C and C_1 mixture (13, 14). 52
Figure S75: HMBC spectrum (500 MHz, DMSO-d_6) of 2-NAC-*E*-lipothrenin C and C_1 mixture (13, 14). 53
Figure S76. The HRESIMS of compound 15. 53
Figure S77: ^{1}H NMR spectrum (500 MHz, DMSO-d_6) of *iso*-lipothrenin A (15). 54
Figure S78: ^{1}H-^{1}H-COSY spectrum (500 MHz, DMSO-d_6) of *iso*-lipothrenin A (15). 54
Figure S79: HSQC spectrum (500 MHz, DMSO-d_6) of *iso*-lipothrenin A (15). 55
Figure S80: HMBC spectrum (500 MHz, DMSO-d_6) of *iso*-lipothrenin A (15). 55
Figure S81. The HRESIMS of compound 16. 56
Figure S82: ^{1}H NMR spectrum (500 MHz, DMSO-d_6) of 14-methyl-lipothrenin A (16). 56
Figure S83: ^{13}C NMR spectrum (500 MHz, DMSO-d_6) of 14-methyl-lipothrenin A (16). 57
Figure S84: ^{1}H-^{1}H-COSY spectrum (500 MHz, DMSO-d_6) of 14-methyl-lipothrenin A (16). 57
Figure S85: HSQC spectrum (500 MHz, DMSO-d_6) of 14-methyl-lipothrenin A (16). 58
Figure S86: HMBC spectrum (500 MHz, DMSO-d_6) of 14-methyl-lipothrenin A (16). 58
Figure S87. The HRESIMS of compound 17. 59
Figure S88: ^{1}H NMR spectrum (500 MHz, DMSO-d_6) of 15-methyl-lipothrenin A (17). 59

Figure S89: ^{13}C NMR spectrum (500 MHz, DMSO-d_6) of 15-methyl-lipothrenin A (17). 60

Figure S90: ^{1}H-^{1}H-COSY spectrum (500 MHz, DMSO-d_6) of 15-methyl-lipothrenin A (17). 60

Figure S91: HSQC spectrum (500 MHz, DMSO-d_6) of 15-methyl-lipothrenin A (17). 61

Figure S92: HMBC spectrum (500 MHz, DMSO-d_6) of 15-methyl-lipothrenin A (17). 61

Figure S93. ^{1}H NMR spectrum (500 MHz, DMSO-d_6) of L-Lipothrenin B (3) isolated from deletion mutant I6_ΔlitM. 62

Figure S94. ^{13}C NMR spectrum (500 MHz, DMSO-d_6) of L-Lipothrenin B (3) isolated from deletion mutant I6_ΔlitM. 62

Figure S95. ^{1}H NMR spectra of compound 7, compound mixture 2, 3 and compound 3 isolated from deletion mutant I6_ΔlitM. (500 MHz, DMSO-d_6); compound 7 identifies shift and peak splitting of protons belonging to D-threonine previously determined by Marfey's method; L-threonine proton shifts and peak splitting are determined *vis versa* and identifying the lipothrenin B derivative from I6_ΔlitM as L-lipothrenin B (3). 63

Heterologous Expression and Genetic Manipulation

Table S1: Strains, Plasmids and BACS used in this study

Strain/Plasmids/BACS	Description	Source
**Strains**		
Streptomyces aureus LU18118	Wild type strain with lipothrenin cluster	BASF
Streptomyces albus Δ14	Optimized heterologous host strain	Myronovskyi et al.[1]
Streptomyces lividans Δ8	Optimized heterologous host strain	Ahmad et al.[2]
Escherichia coli GB05 pUB307	General cloning host	(HIPS Saarland)
Escherichia coli ET12567 pUB307	Donor strain for intergeneric conjugation	Flett et al.[3]
**Plasmids and BACS**		
pUC19	Plasmid; General cloning vector; Used as a source of ampicillin resistance marker for RedET experiments	Thermo Scientific
I6	BAC containing lipothrenin cluster	This study
I6_ΔLS1	Derivative of I6; contains substitution of the genes orf(-24) – orf (-12) with ampicillin resistance gene	This study
I6_ΔLS2	Derivative of I6; contains substitution of the genes orf(-24) – orf (-08) with ampicillin resistance gene	This study
I6_ΔLS3	Derivative of I6; contains substitution of the genes orf(-24) – orf (-04) with ampicillin resistance gene	This study
I6_ΔLS4	Derivative of I6; contains substitution of the genes orf(-24) – *litA* with ampicillin resistance gene	This study
I6_ΔlitB	Derivative of I6; contains substitution of the gene *litB* with ampicillin resistance gene	This study
I6_ΔlitD	Derivative of I6; contains substitution of the gene *litD* with ampicillin resistance gene	This study
I6_ΔlitE	Derivative of I6; contains substitution of the gene *litE* with ampicillin resistance gene	This study
I6_ΔlitG	Derivative of I6; contains substitution of the gene *litG* with ampicillin resistance gene	This study
I6_ΔlitH	Derivative of I6; contains substitution of the gene *litH* with ampicillin resistance gene	This study
I6_ΔlitJ	Derivative of I6; contains substitution of the gene *litJ* with ampicillin resistance gene	This study
I6_ΔlitL	Derivative of I6; contains substitution of the gene *litL* with ampicillin resistance gene	This study
I6_ΔlitM	Derivative of I6; contains substitution of the gene *litM* with ampicillin resistance gene	This study
I6_ΔlitN	Derivative of I6; contains substitution of the gene *litN* with ampicillin resistance gene	This study

I6_ΔlitO	Derivative of I6; contains substitution of the gene *litO* with ampicillin resistance gene	This study
I6_ΔlitP	Derivative of I6; contains substitution of the gene *litP* with ampicillin resistance gene	This study
I6_ΔRS	Derivative of I6; contains substitution of the genes *litQ* – orf(01) with ampicillin resistance gene	This study
I6_ΔRS1	Derivative of I6; contains substitution of the genes orf(01) – orf(04) with ampicillin resistance gene	This study

Table S2: Used primers for RedET.

Gene #	Oligo Name	Sequence
orf24-orf11	ΔLS1_f	ATGAGTGAACTTGCGAGCGCAGTTCGGATCCGGGCCGCCGGGCCCGACGGTTTAAACCGTCAGGTGGCACTTTTCG
orf24-orf11	ΔLS1_r	TTACTGACCGACGAGGCCCGGCTGCAGCGTCTTCCTGAACAGCACCGTGTTTAAACTTACCAATGCTTAATCAGTG
orf24-orf09	ΔLS2_f	ATGAGTGAACTTGCGAGCGCAGTTCGGATCCGGGCCGCCGGGCCCGACGGTTTAAACCGTCAGGTGGCACTTTTCG
orf24-orf09	ΔLS2_r	TCAGAGAGCCGCGAGTTCGTCCACCAGGTCGTCCAGACCCAGCGACCCGTTTAAACTTACCAATGCTTAATCAGTG
orf24-orf03	ΔLS3_f	ATGAGTGAACTTGCGAGCGCAGTTCGGATCCGGGCCGCCGGGCCCGACGGTTTAAACCGTCAGGTGGCACTTTTCG
orf24-orf03	ΔLS3_r	TCACCGCCCCACGGGACGGCGCGGCAGCGGCGAGGAGTACACCACGCCGTTTAAACTTACCAATGCTTAATCAGTG
orf24-litA	ΔLS4_f	ATGAGTGAACTTGCGAGCGCAGTTCGGATCCGGGCCGCCGGGCCCGACGGTTTAAACCGTCAGGTGGCACTTTTCG
orf24-litA	ΔLS4_r	TTACGTGGTGAGGAGGGTTCGCAGGGTGGCTGCCTCCGGTACGCCGAGGTTTAAACTTACCAATGCTTAATCAGTG
litB	ΔlitB_f	TCAGGGGATGCGGACCACCGTGCCCGCGTACGTCAGGCCCGCGCCGAAGGTTTAAACCGTCAGGTGGCACTTTTCG
litB	ΔlitB_r	ATGGCCGCCACGGCCGCGGGGAAGGCGCTCGCCGACGCGGGCGCGGCGGTTTAAACTTACCAATGCTTAATCAGTG
litD	ΔlitD_f	ATGAACACAGTCCTCGACTGGCTGGACTCCCCCGCGCCCGAGCGTGGAAGTTTAAACCGTCAGGTGGCACTTTTCG
litD	ΔlitD_r	TCATATGGCAGCCCCATCCAGTCGCGGTGCGGTCGGTTCGGTCGGGTCGTTTAAACTTACCAATGCTTAATCAGTG

litE	ΔlitE_f	ATGAGCGTCACGACCCCCGGGCTCGTACTCGACGTCACCGAGACGGCCCGTTTAAACCGTCAGGTGGCACTTTTCG
litE	ΔlitE_r	TCAGGCCGCGGCGGCCCGGATCGGGTGCGGCGCGTTCCAGCGCTCGGCGTTTAAACTTACCAATGCTTAATCAGTG
litG	ΔlitG_f	ATGACGACTTACGATCTGCGCAGCGAGACGGACGACCCGCTGGCGCTCTGTTTAAACCGTCAGGTGGCACTTTTCG
litG	ΔlitG_r	TCACGTCTCCGCGCGCTGCGCGCTGCGCGCGCCGAGGGCCTTGACGATGTTTAAACTTACCAATGCTTAATCAGTG
litH	ΔlitH_f	ATGGGCGCCGGCCTCACCGCGCGGTACGTCGTGGTCGGCGCGGGCTCGGGTTTAAACCGTCAGGTGGCACTTTTCG
litH	ΔlitH_r	TCAGCGGCCGGCGAGGGCCCCGCGGCGGTGCTGCGCCGCCTCGGCGAGGTTTAAACTTACCAATGCTTAATCAGTG
litJ	ΔlitJ_f	GTGATCGCGACCACCACCACCGCCGTACCGAACGCCCCGACCCTCTCCGGTTTAAACCGTCAGGTGGCACTTTTCG
litJ	ΔlitJ_r	TCACAGCCGGCGCCCGTACAGCGCACCGGTAATGGCCAGCGCAAGCACGTTTAAACTTACCAATGCTTAATCAGTG
litL	ΔlitL_f	GTGACCTCGCAGACCGTCACCGGTGTGACACCGCCGGCGGACGACGCGCGTTTAAACCGTCAGGTGGCACTTTTCG
litL	ΔlitL_r	TCATATCCTCACCCTCCGGATCTCTCCATCGACCCGGCCGACCAACTGGTTTAAACTTACCAATGCTTAATCAGTG
litM	ΔlitM_f	ATGAGCACCACCACCGCGGCCAGGACGGTTCCGGCCGGTGTCCCCGAGGGTTTAAACCGTCAGGTGGCACTTTTCG
litM	ΔlitM_r	TCACGACTCCTTGTCCTGTGTGCGCTGAGTGCTGTCCGAAAAGGCATCGTTTAAACTTACCAATGCTTAATCAGTG
litN	ΔlitN_f	ATGACCACCGCCACCGACCGCCCGGGCCGTCGCTCGGAGGACCCGCTCGGTTTAAACCGTCAGGTGGCACTTTTCG
litN	ΔlitN_r	CTAGGCATCCTTGAGCCAGCCGGCCGAGCGCCACACCTTCTCCGAGCGGTTTAAACTTACCAATGCTTAATCAGTG
litO	ΔlitO_f	GTGGACGATGACGGGTACGAAGGTCCGGCGGTGCTGGCGGTGGAAGGCCGTTTAAACCGTCAGGTGGCACTTTTCG
litO	ΔlitO_r	TCAGTTGCCGGAGGTACCGAATTCCGAAACTTCTTCGATGGTGAACGGGTTTAAACTTACCAATGCTTAATCAGTG
litP	ΔlitP_f	ATGTGCTTCGACGAGGACGCCATTCCACCGGTACCGCAGGTTCCGGATGGTTTAAACCGTCAGGTGGCACTTTTCG

litP	ΔlitP_r	TCAGCCCGCCCCGACCCGGTCCACGAACCGCAGCACCCGGTCCCAGGCGTTTAAACTTACCAATGCTTAATCAGTG
orf01-orf04	ΔRS1_f	TCAGGCGTCCCCGTACGCCTCCCCGCCCAGCTCGAATCCGGCCGTCCCCGTTTAAACCGTCAGGTGGCACTTTTCG
orf01-orf04	ΔRS1_r	TCAGGCGAGGCGCGGGATCTCGATCGCGGGGCAGCGGTCCATCACCATGTTTAAACTTACCAATGCTTAATCAGTG
litQ-orf01	ΔRS_f	ATGCCCACGACTCGTACGAGAACGCGTCCGACGCAGCGTGAGCGGTCCGGTTTAAACCGTCAGGTGGCACTTTTCG
litQ-orf01	ΔRS_r	ATGCAGGACGAGTACCGCACCGTGGCCCGCGCGGGTGTGCACGAGACCGTTTAAACTTACCAATGCTTAATCAGTG

Table S3: Used primers for PCR.

Gene #	Oligo Name	Sequence
orf24-orf11	ΔLS1_seqf	TTGCACAACATGGGGGATCA
orf24-orf11	ΔLS1_seqr	GAGCAGGTGGTTCAGGTAGC
orf24-orf09	ΔLS2_seqf	TCCTTGAGAGTTTTCGCCCC
orf24-orf09	ΔLS2_seqr	GTCTGTCTGCACCACCATGT
orf24-orf03	ΔLS3_seqf	GTGACACCACGATGCCTGTA
orf24-orf03	ΔLS3_seqr	CGAACGCGAACACGAACTG
orf24-litA	ΔLS4_seqf	GTGACACCACGATGCCTGTA
orf24-litA	ΔLS4_seqr	CATGTCCGACATCCTCGACC
litB	ΔlitB_seqf	CGTCTGTACGTGCGTCAGG
litB	ΔlitB_seqr	ACAGGAGTCCGTGTGAACAC
litD	ΔlitD_seqf	TCACCAAGAAGTCGATGGCG
litD	ΔlitD_seqr	GTACAGAGCAGCGGAGTCG
litE	ΔlitE_seqf	GAACGATTCCGCGGACCTC
litE	ΔlitE_seqr	GTCCTCGATGTCCTTGACCG
litG	ΔlitG_seqf	GCCAGGAAAGGGACGAAAGA
litG	ΔlitG_seqr	CCTCGATGAGCACCACCTG
litH	ΔlitH_seqf	TGACCAACGTCTTCGAGTCG
litH	ΔlitH_seqr	GAACTGGAGGGAGAAGTCGC
litJ	ΔlitJ_seqf	CCACATGACACGAAAGGCAG
litJ	ΔlitJ_seqr	GTACGACACCGCGGAGAAG
litL	ΔlitL_seqf	GTACGTCGAGGCCATGGTG
litL	ΔlitL_seqr	GCCGGTTGAGGTTGTTCAGT
litM	ΔlitM_seqf	CGTCTGTCGCTGGAACTGAT
litM	ΔlitM_seqr	CGTACAGGGAGATCCGCTTC
litN	ΔlitN_seqf	GTACGTGGTCGAGATCCTGG
litN	ΔlitN_seqr	CCATGCCTTCGATCCGGTAG
litO	ΔlitO_seqf	ACAACCCGCTGATGTACGAG

litO	ΔlitO_seqr	GTAGAACCCGTACAGGCCG
litP	ΔlitP_seqf	CTACCGGATCGAAGGCATGG
litP	ΔlitP_seqr	GCTCTGAAGAGGTGCTGCTT
orf01-orf04	ΔRS1_seqf	GTGAACACTGCGACAACCAC
orf01-orf04	ΔRS1_seqr	CAGTGCTGCAATGATACCGC
litQ-orf01	ΔRS_seqf	CACAGCTTCTTCGACGCCAA
litQ-orf01	ΔRS_seqr	CTGAAGGTGATCCGCGAGAC

Chromatograms from Gene Deletion Experiments

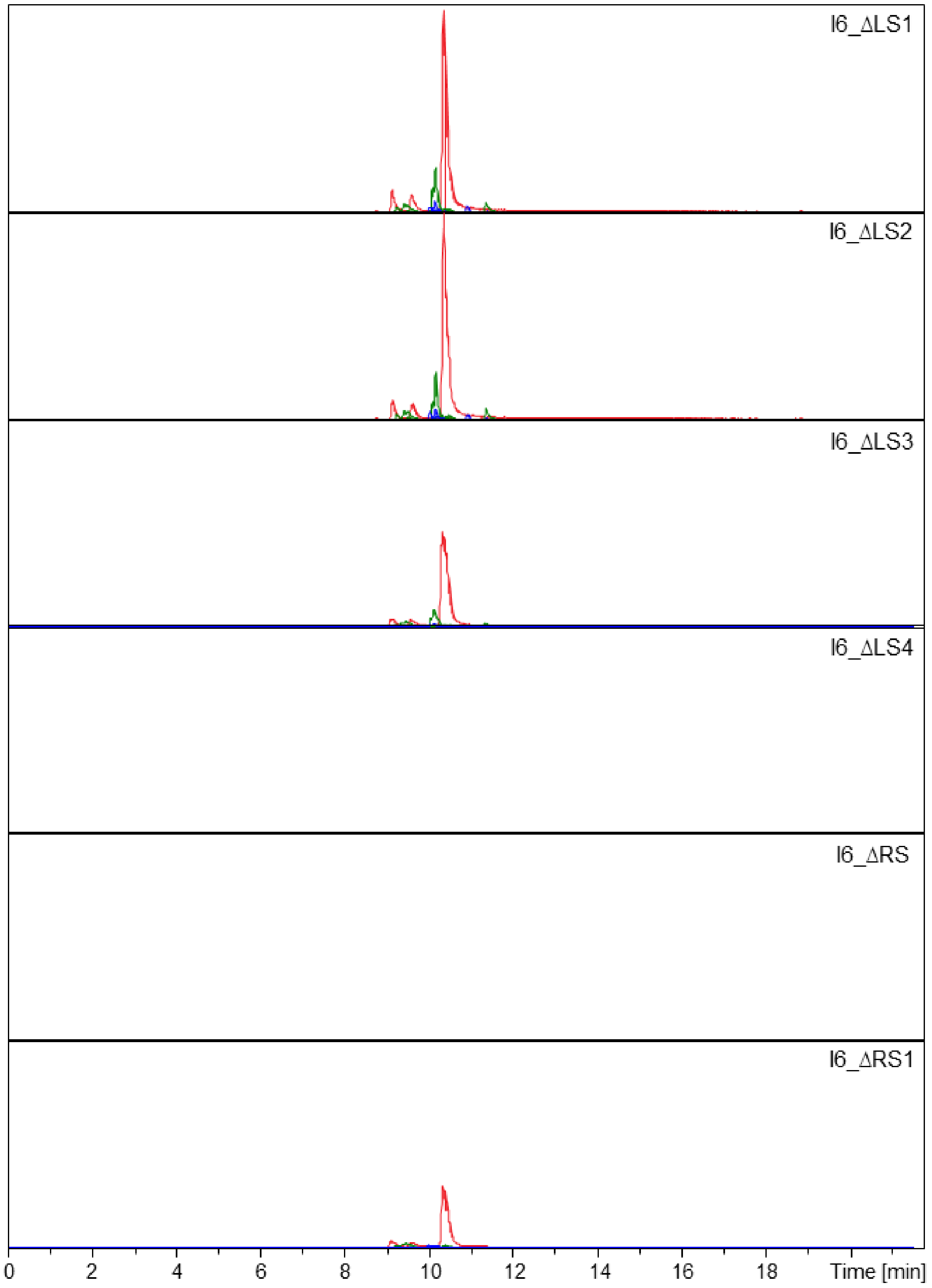

Figure S1. Chromatogram showing the extracted masses from lipothrenin A (**1**, **4** and **5**, red), lipothrenins B (**2**, **3** and **7-10**, green) and lipothrenin C (**4** and **11-14**, blue) from butanol extract from deletion mutants covering the flanking regions of the *lit* BGC.

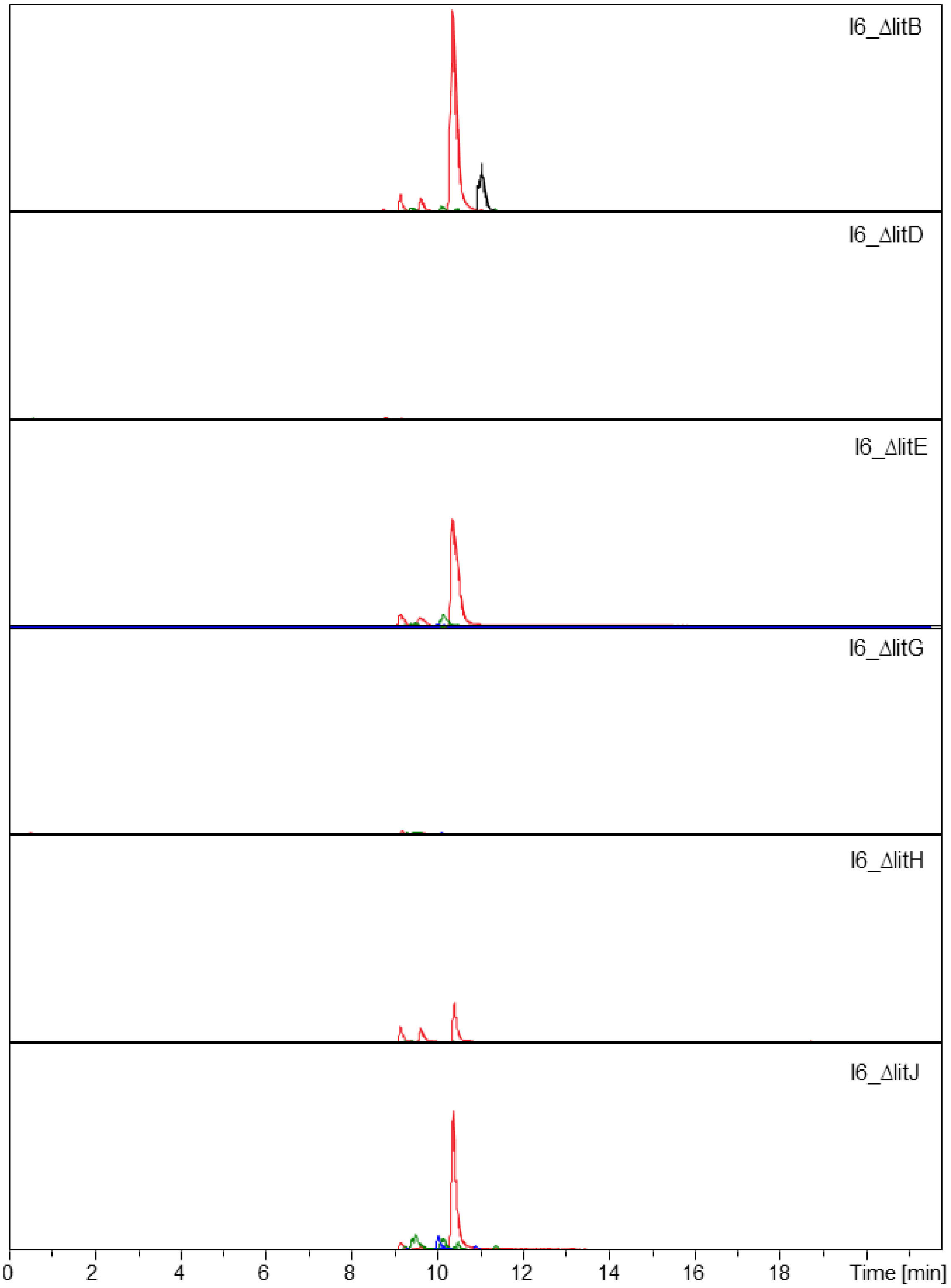

Figure S2. Chromatogram showing the extracted masses from lipothrenins A (**1**, **4** and **5**, red) and lipothrenin A derivatives **15** – **17** (black), lipothrenins B (**2**, **3** and **7-10**, green) and lipothrenins C (**4** and **11-14**, blue) from butanol extracts from deletion mutants covering I6_ΔlitB – litJ.

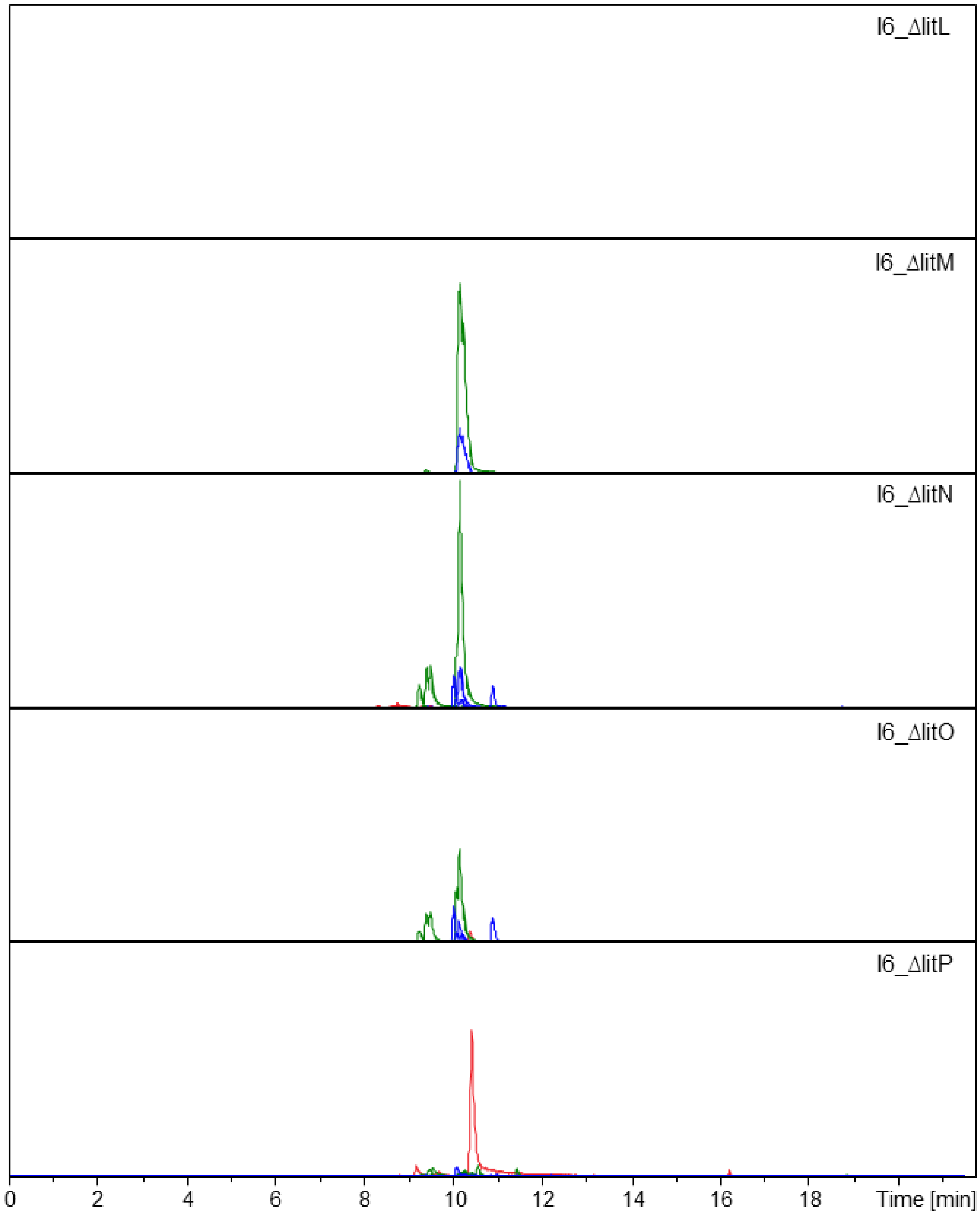

Figure S3. Chromatogram showing the extracted masses from lipothrenins A (**1**, **4** and **5**, red), lipothrenins B (**2**, **3** and **7-10**, green) and lipothrenins C (**4** and **11-14**, blue) from butanol extracts from deletion mutants covering I6_ΔlitL – litP.

Stereochemistry Analysis by Marfey's Method

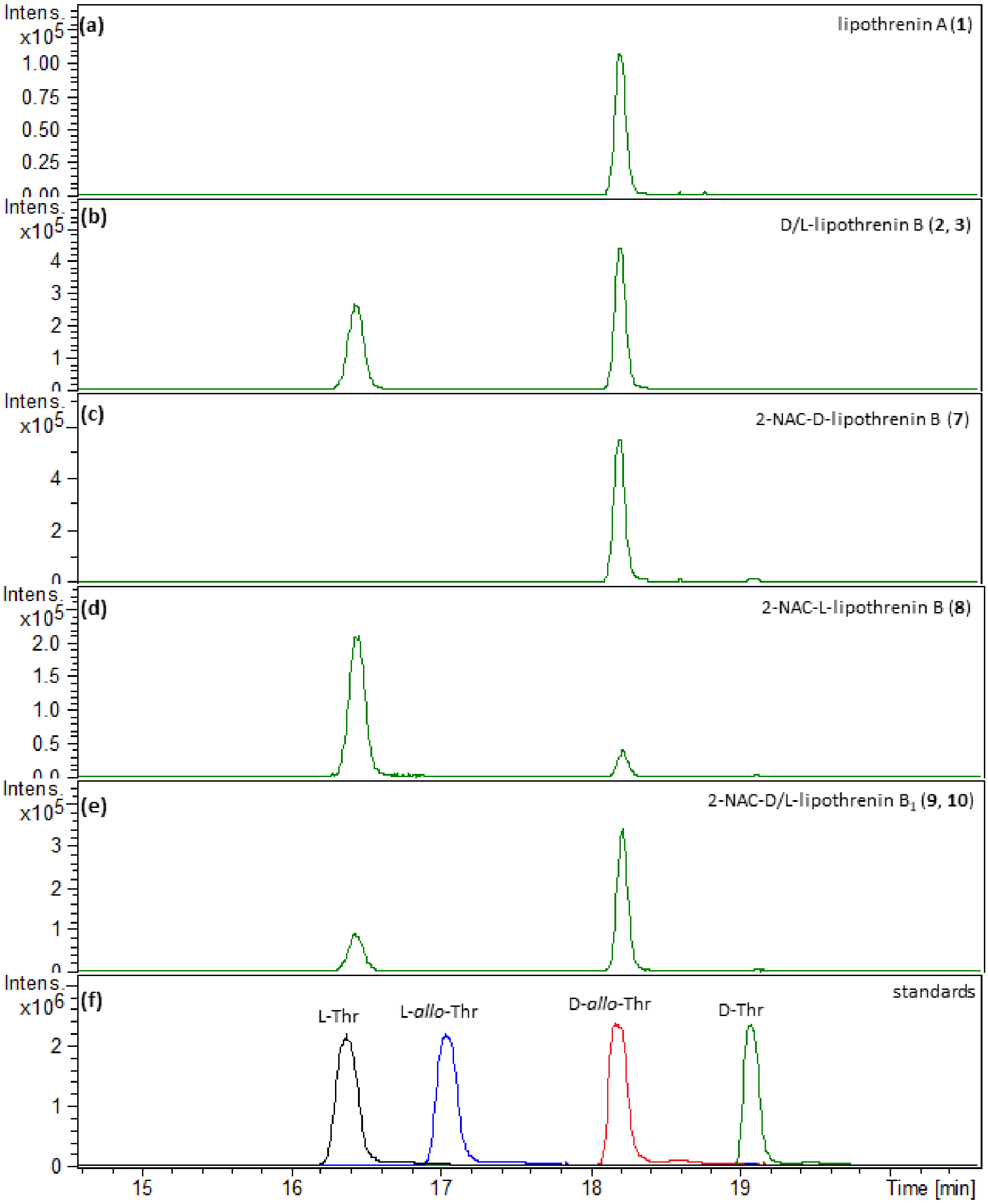

Figure S4: LC-MS chromatograms showing the extracted mass of threonine of hydrolysed **1** (**a**), **2** and **3** (**b**) **7-10** (**c-e**) and the references L-, D-, L-*allo*- and D-*allo*-threonine (**f**) derivatized with L-FDLA.

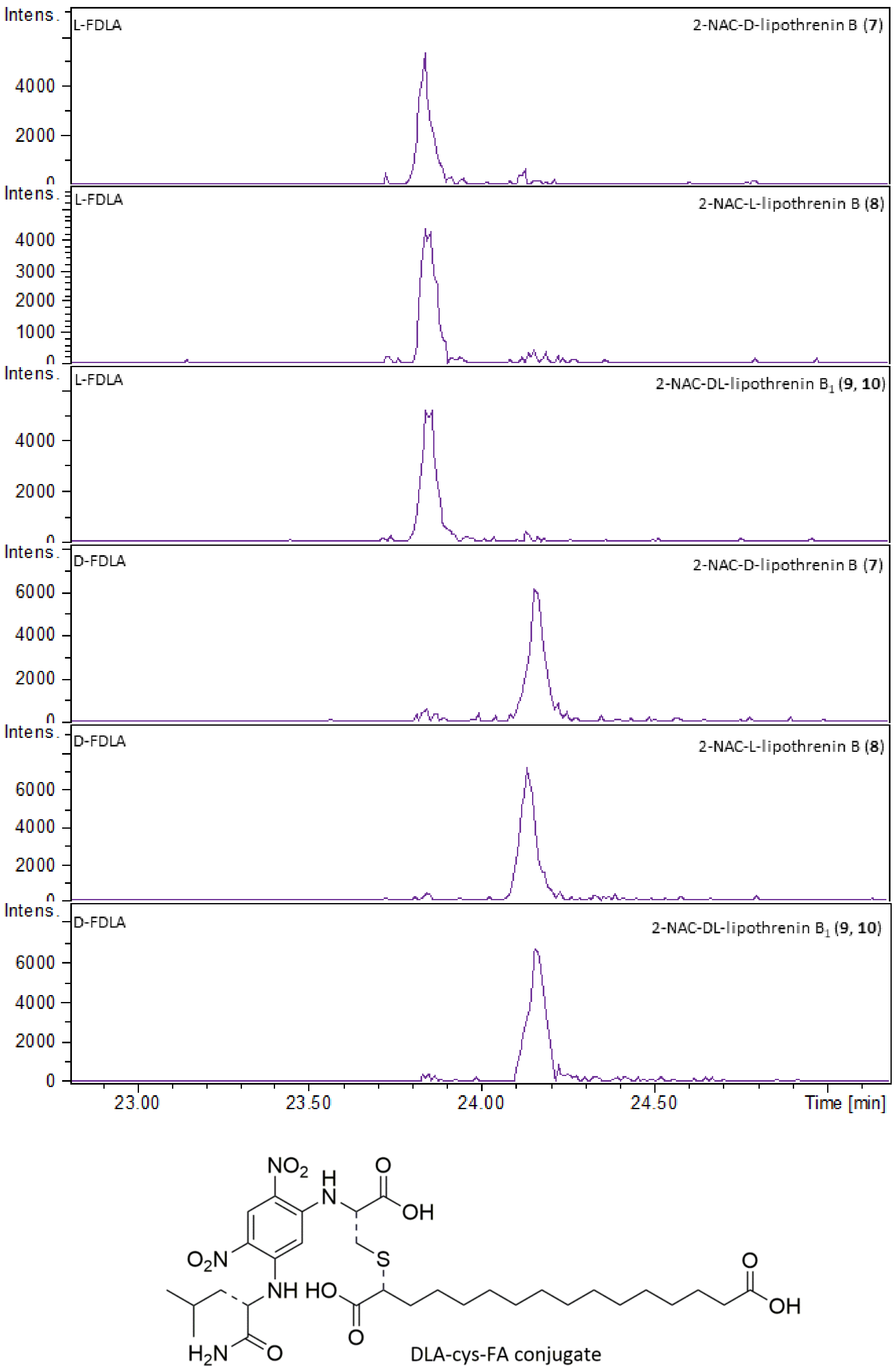

Figure S5: LC-MS chromatograms of the extracted masse of the DLA-cys-FA conjugate derived from hydrolysis of 2-NAC-DL-lipothrenin B/B_1 and derivatisation with L- and D-FDLA.

NMR Spectroscopy Data

Table S4: NMR data (500 MHz, DMSO-*d6*) for *Iso*-lipothrenin A (**15**)

No	δC (type)	δH, mult. (*J* in Hz)
1	170.7, C	
2	63.2, CH	4.57, d (8.6)
3	64.3, CH	4.09, dq (6.2, 8.8)
4	20.1, CH_3	1.03, d (ovl.)
5	173.5, C	
6	31.4, CH_2	2.32, m
7	24.0, CH_2	1.47, m
8-16	28.4-29.0, CH_2	1.23, bs (18H)
17	33.2, CH_2	1.51, m
18	38.5, CH	2.28, m
19	177.2, C	
20	16.8, CH_3	1.03, d (ovl.)

ovl. = overlap

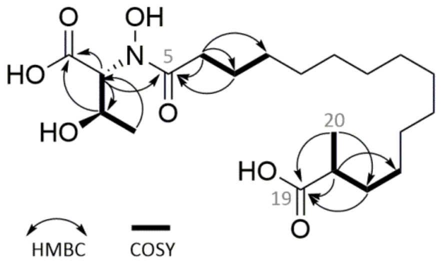

Figure S6. Structure of compound **15** with selected COSY (—) and HMBC (⌒) correlations.

Table S5: NMR data (500 MHz, DMSO-*d6*) for 14-methyl-lipothrenin A (**16**)

No	δC (type)	δH, mult. (*J* in Hz)
1	170.6, C	
2	63.5, CH	4.61, d (8.2)
3	64.3, CH	4.12, dq (6.4, 8.4)
4	20.2, CH_3	1.04, d (6.4)
5	173.9, C	
6	31.6, CH_2	2.35, m
7	24.0, CH_2	1.48, m
8-16	28.5-29.0, CH_2	1.23, bs (18H)
17	36.1, CH_2	1.11, m
		1.26, m
18	29.6, CH	1.79, m
19	41.4, CH_2	1.98, dd (8.5, 15.0)
		2.17, dd (6.1, 15.0)
20	174.0, C	
21	19.5, CH_3	0.86, d (6.7)

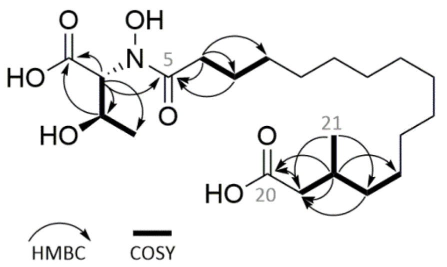

Figure S7. Structure of compound **16** with selected COSY (—) and HMBC (⌒) correlations.

Table S6: NMR data (500 MHz, DMSO-*d6*) for 15-methyl-lipothrenin A (**17**)

No	δ_C (type)	δ_H, mult. (*J* in Hz)
1	170.6, C	
2	63.5, CH	4.62, d (8.6)
3	64.4, CH	4.13, dq (6.4, 8.6)
4	20.2, CH_3	1.04, d (6.4)
5	173.9, C	
6	33.7, CH_2	2.17, t (7.3)
7	24.5, CH_2	1.48, m
8-16	28.5-29.0, CH_2	1.23, bs (18H)
17	26.7, CH_2	1.23, m
18	33.3, CH_2	1.52, m
		1.30, m
19	38.7, CH	2.27, m
20	177.5, C	
21	17.0, CH_3	1.03, d (7.1)

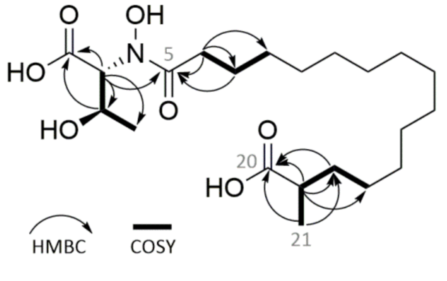

Figure S8. Structure of compound **17** with selected COSY (—) and HMBC (⌒) correlations.

NMR Spectra

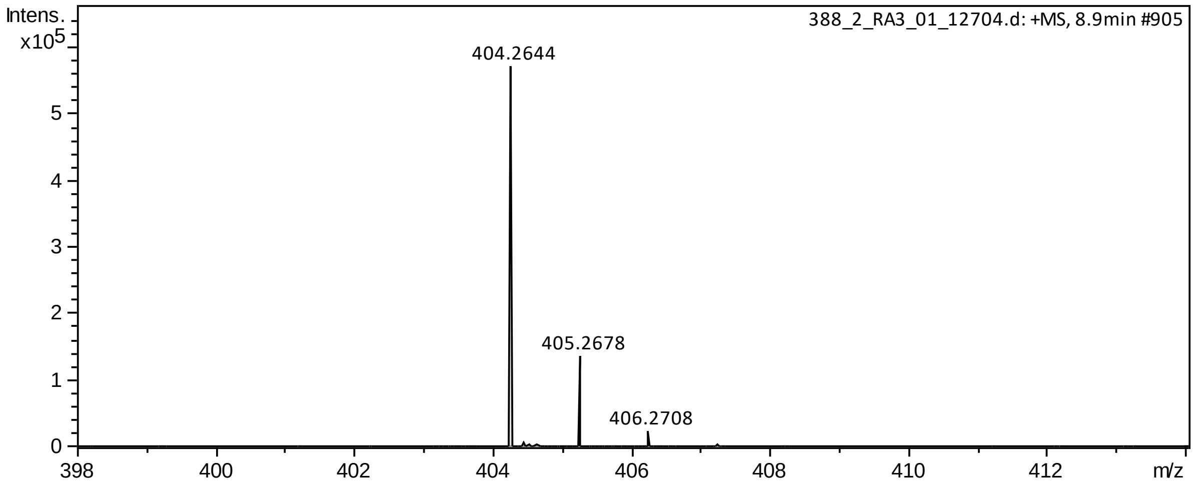

Figure S9. The HRESIMS of compound **1**.

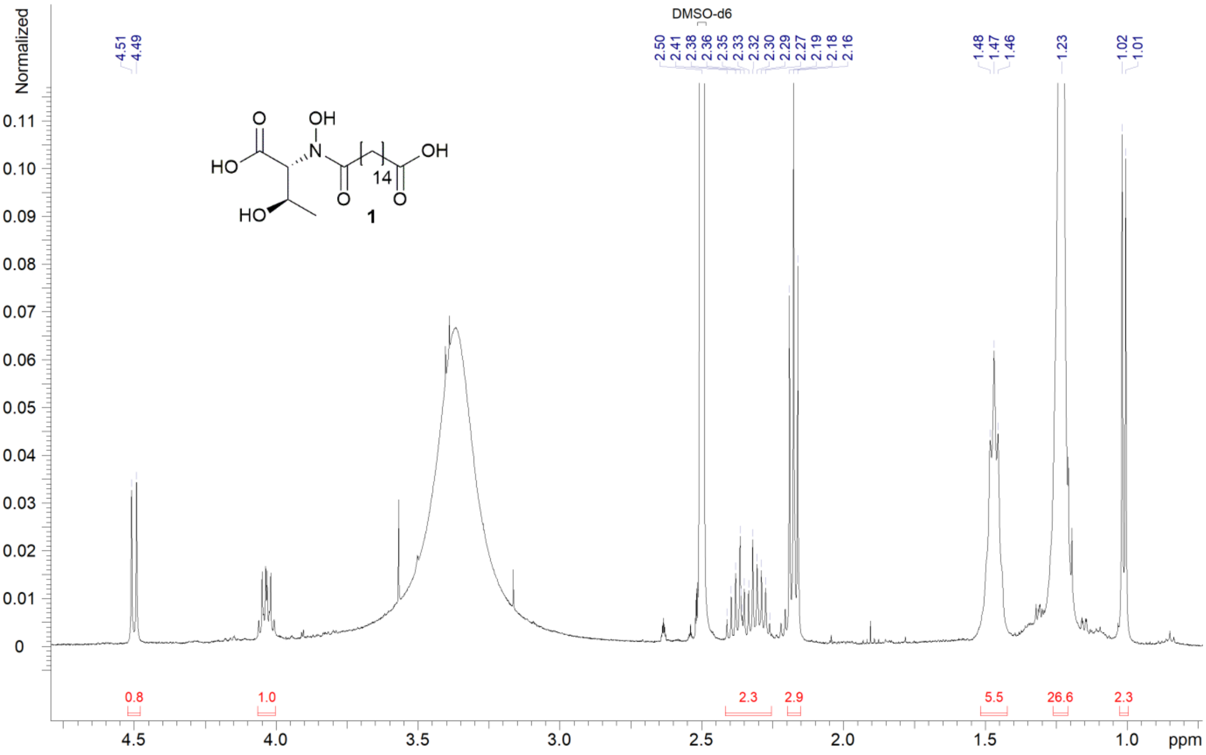

Figure S10. ^{1}H NMR spectrum (500 MHz, DMSO-d_6) of lipothrenin A (**1**).

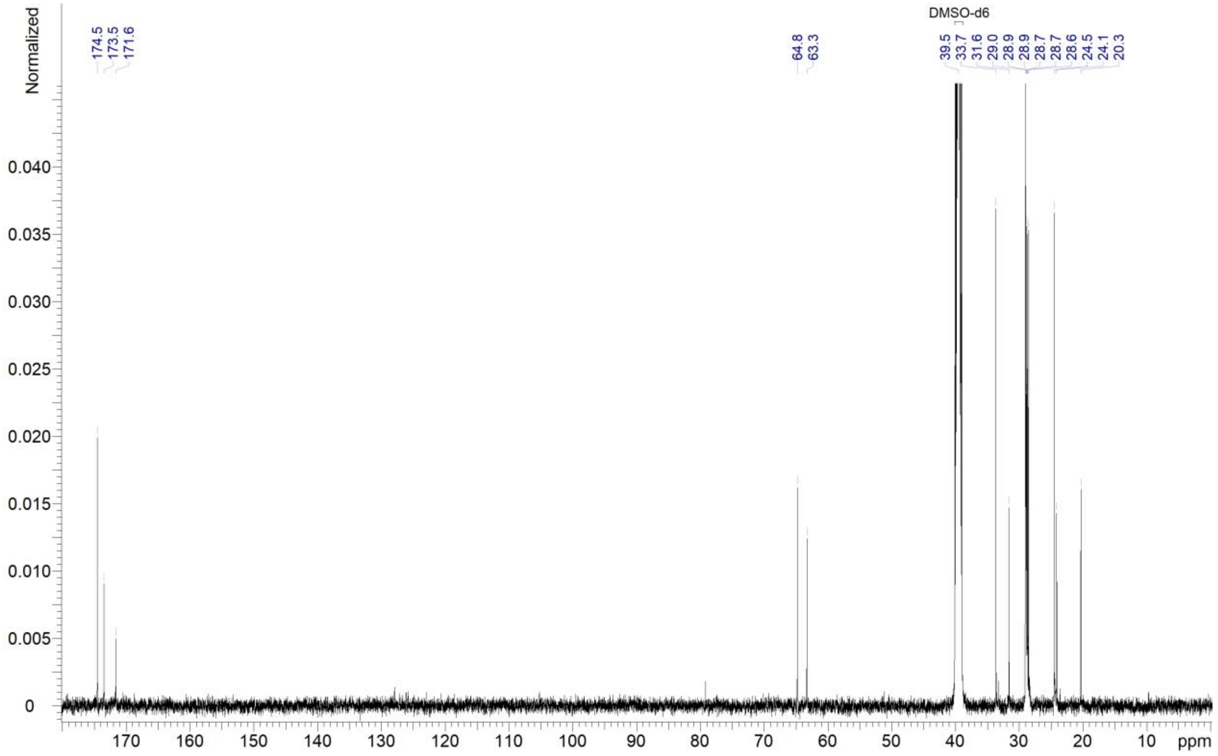

Figure S11: ^{13}C NMR spectrum (500 MHz, DMSO-d_6) of lipothrenin A (**1**).

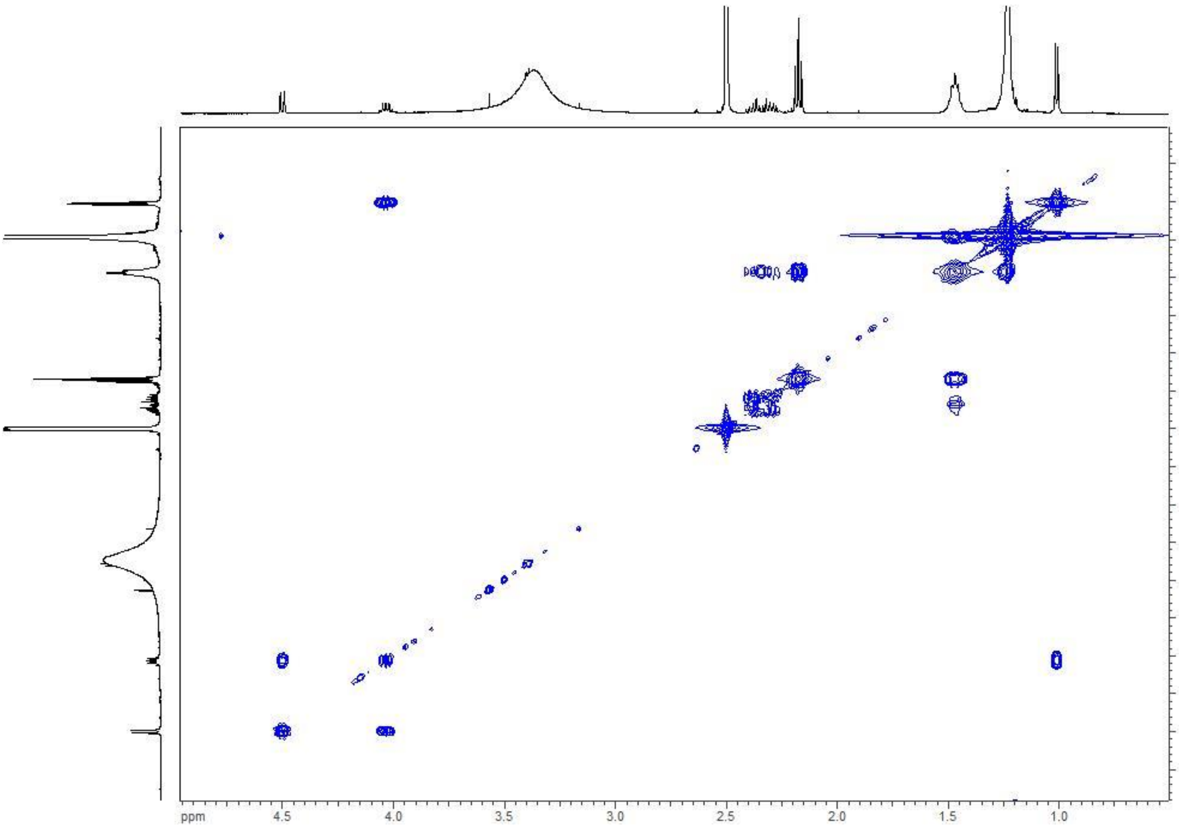

Figure S12: ^{1}H-^{1}H-COSY spectrum (500 MHz, DMSO-d_6) of lipothrenin A (**1**).

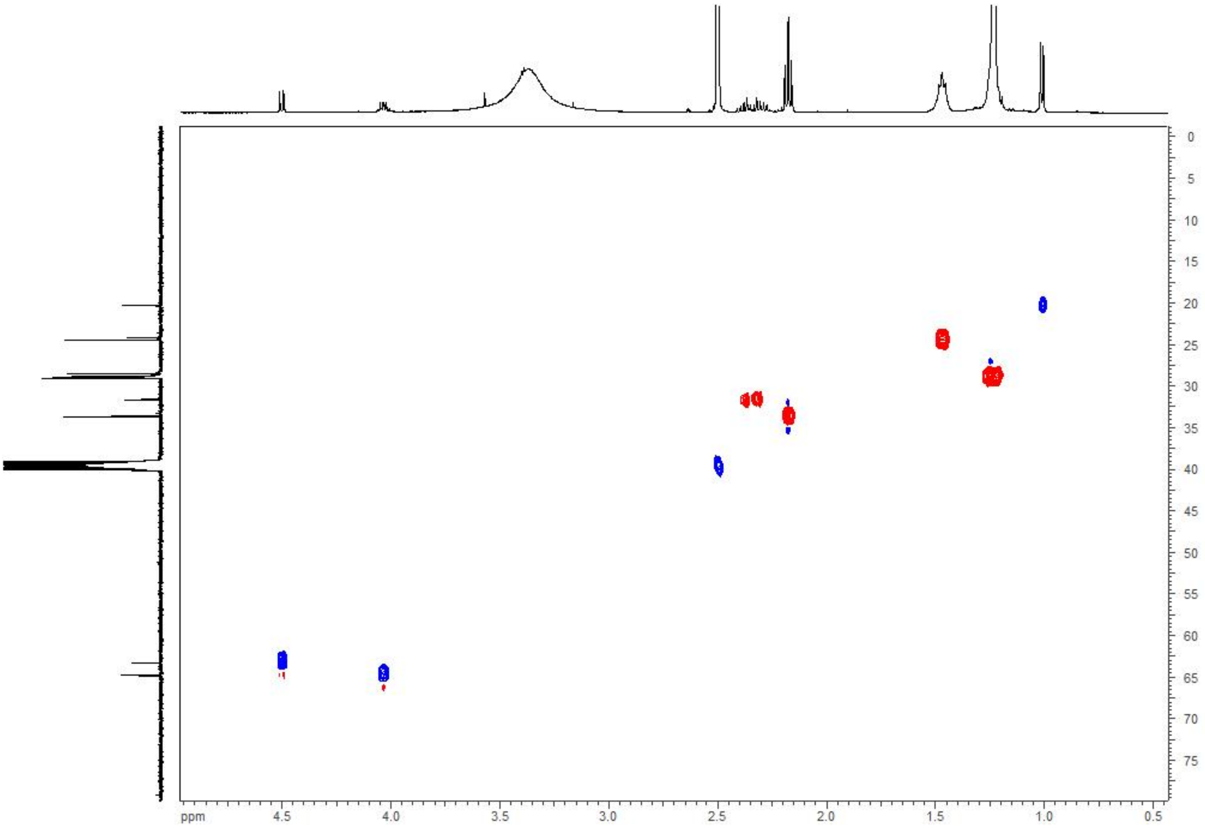

Figure S13: HSQC spectrum (500 MHz, DMSO-d$_6$) of lipothrenin A (**1**).

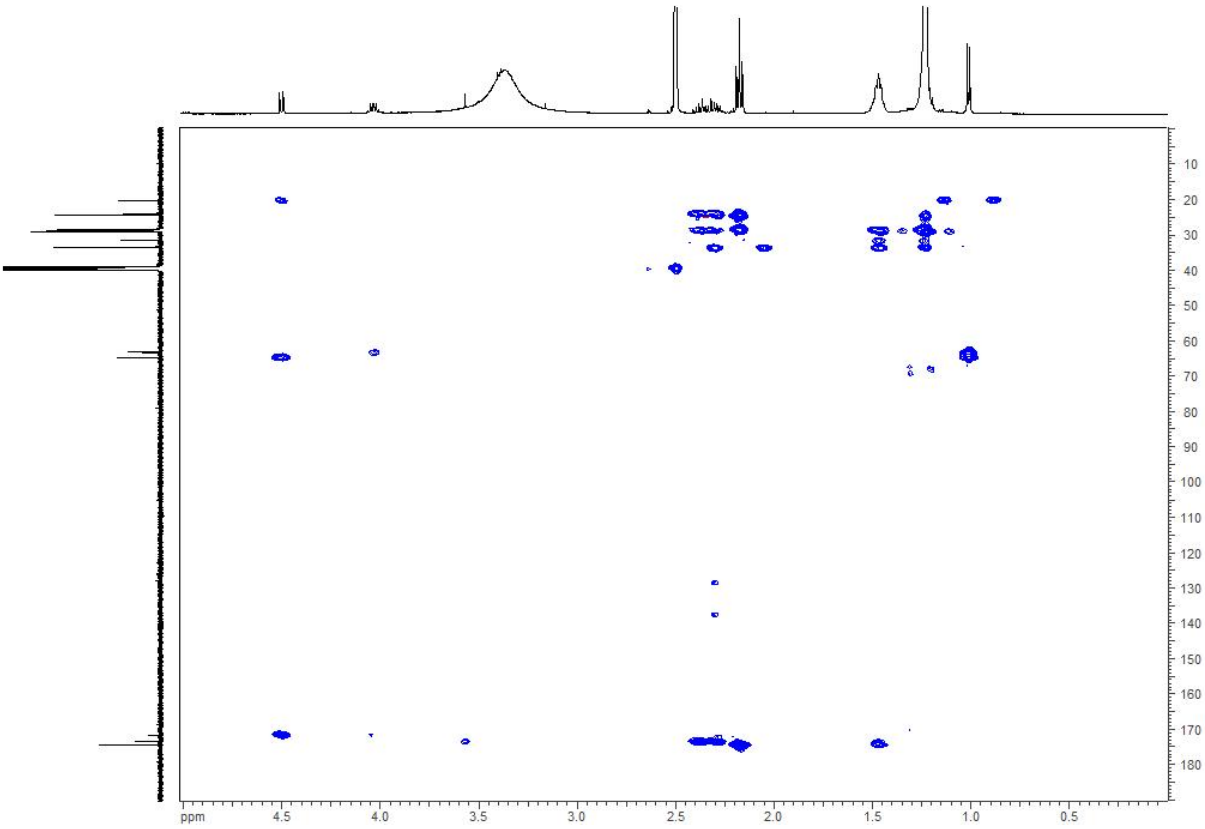

Figure S14: HMBC spectrum (500 MHz, DMSO-d$_6$) of lipothrenin A (**1**).

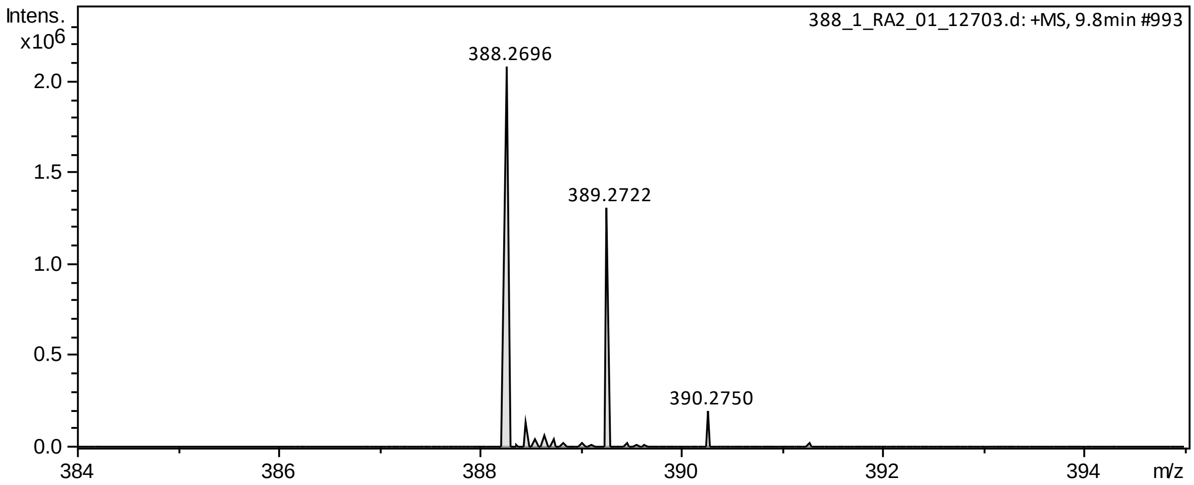

Figure S15. The HRESIMS of compound **2**, **3**.

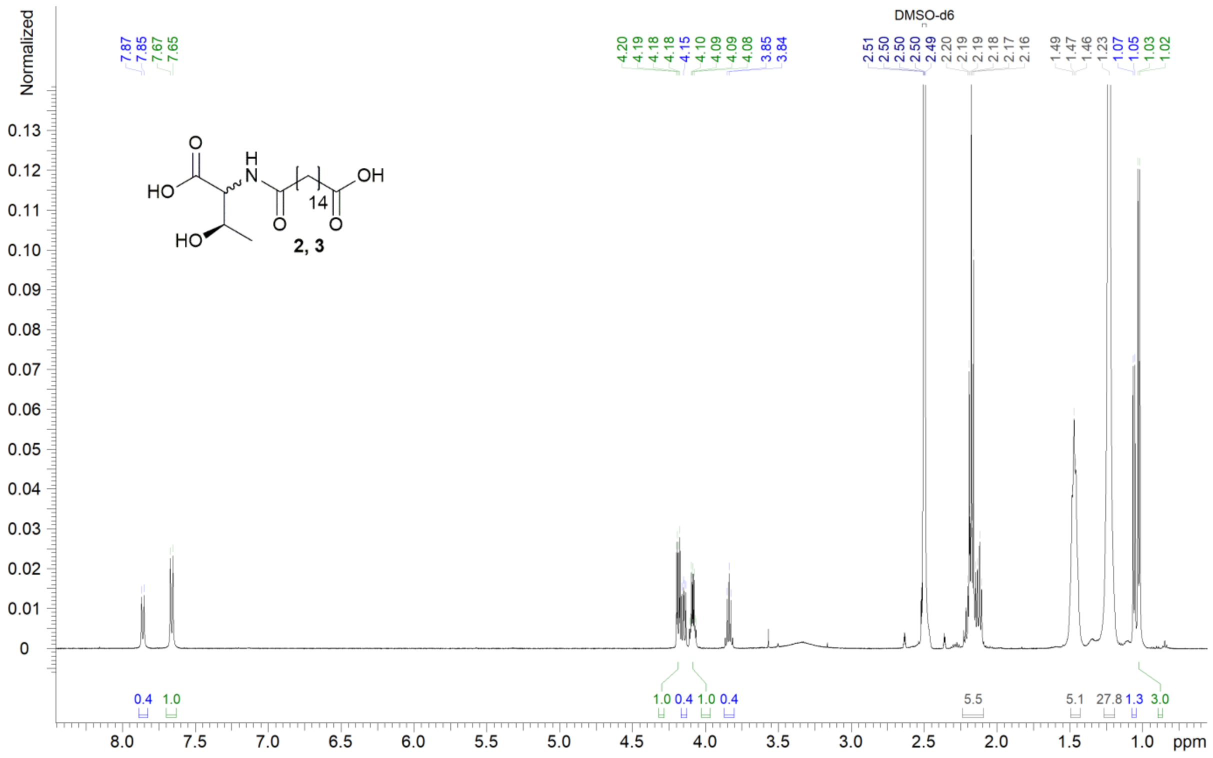

Figure S16: ^{1}H NMR spectrum (500 MHz, DMSO-d$_6$) of D- and L-lipothrenin B (**2, 3**).

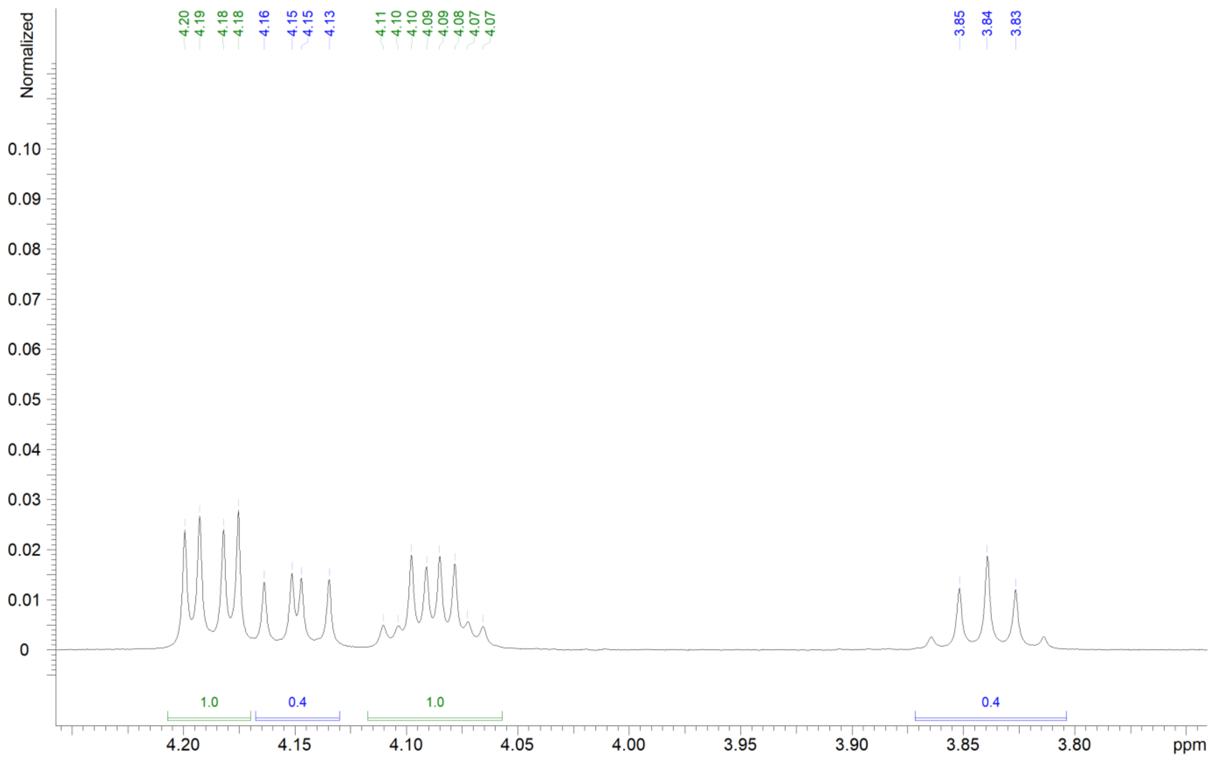

Figure S17. Zoomed in ^{1}H NMR spectrum (500 MHz, DMSO-d$_6$) of D- and L-lipothrenin B (**2, 3**).

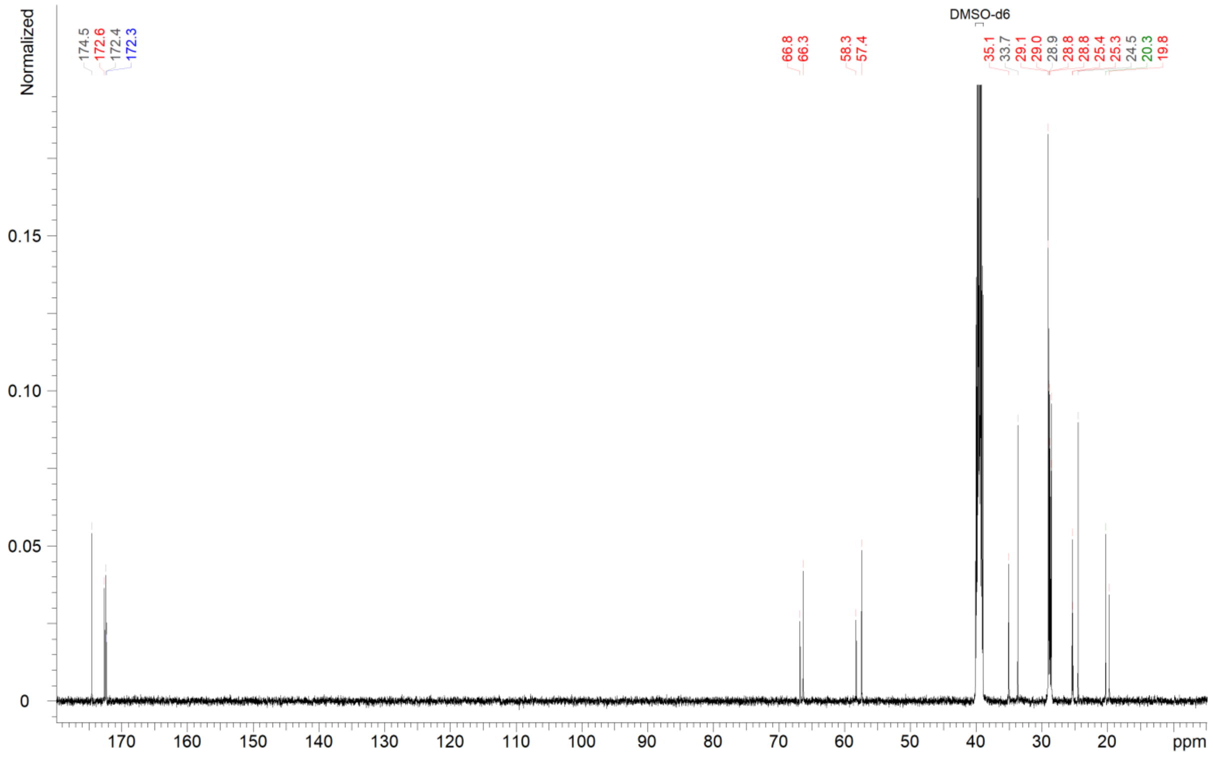

Figure S18: ^{13}C NMR spectrum (500 MHz, DMSO-d$_6$) of D- and L-lipothrenin B (**2, 3**).

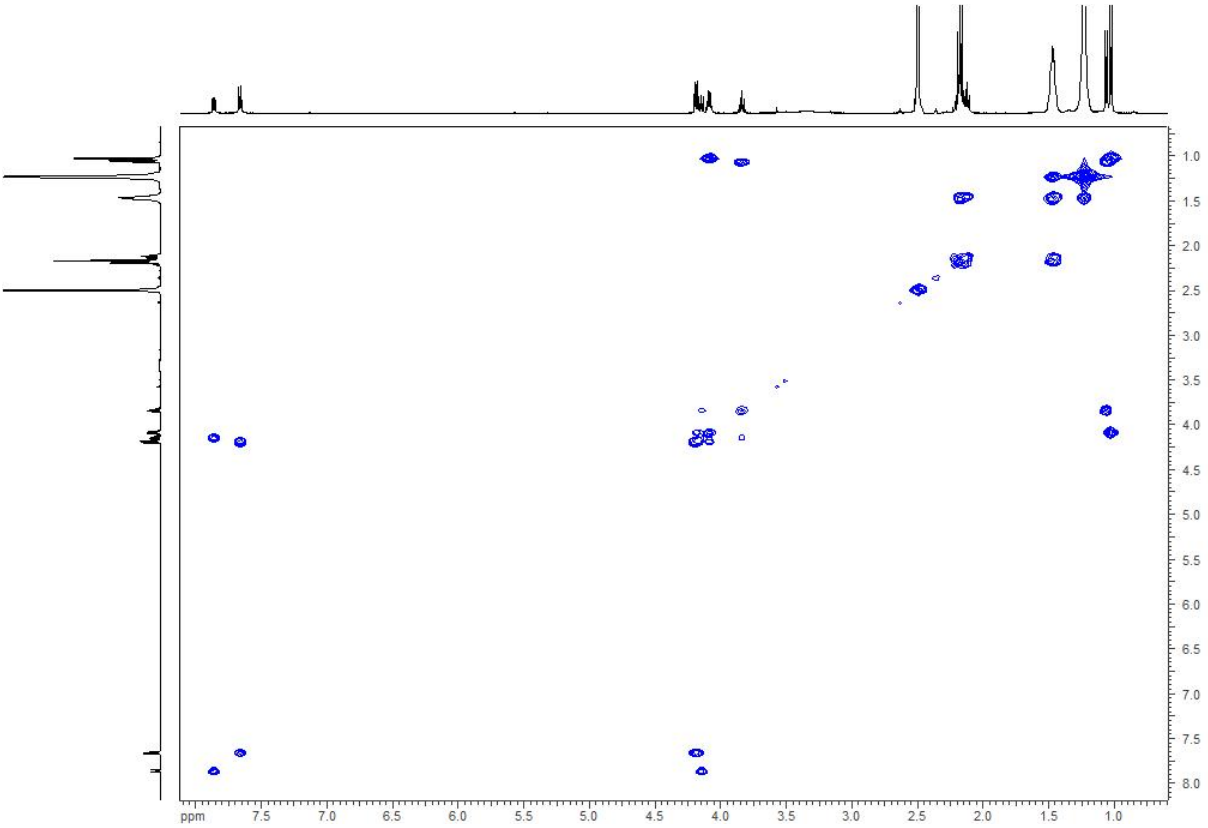

Figure S19: ^{1}H-^{1}H-COSY spectrum (500 MHz, DMSO-d$_6$) of D- and L-lipothrenin B (**2, 3**).

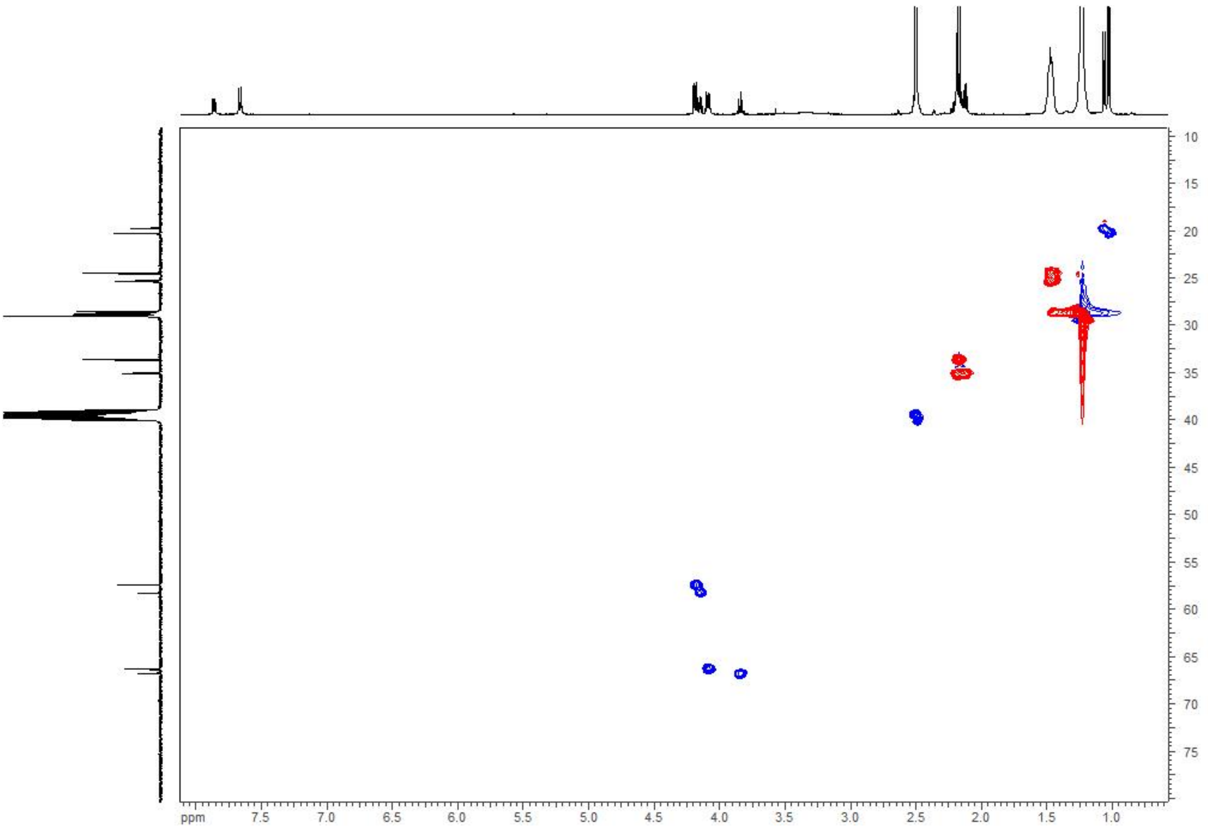

Figure S20: HSQC spectrum (500 MHz, DMSO-d$_6$) of D- and L-lipothrenin B (**2, 3**).

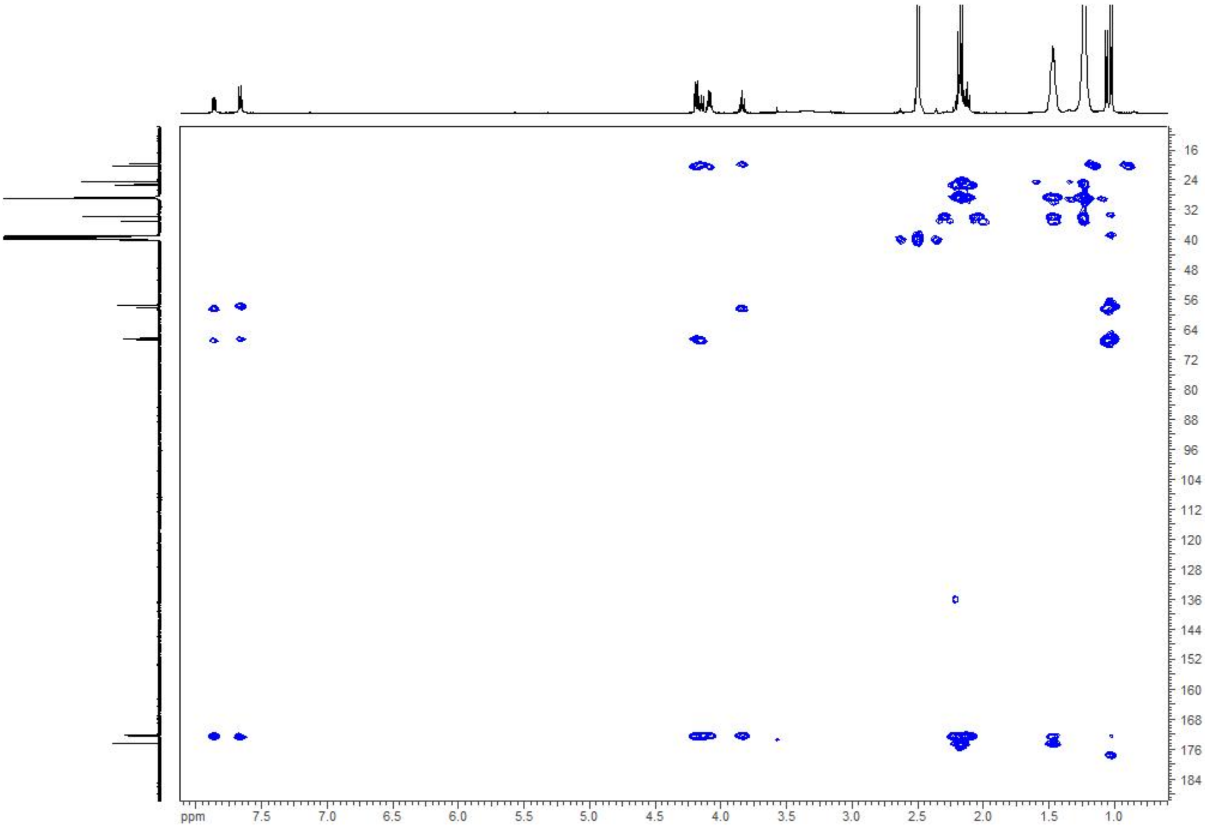

Figure S21: HMBC spectrum (500 MHz, DMSO-d$_6$) of D- and L-lipothrenin B (**2, 3**).

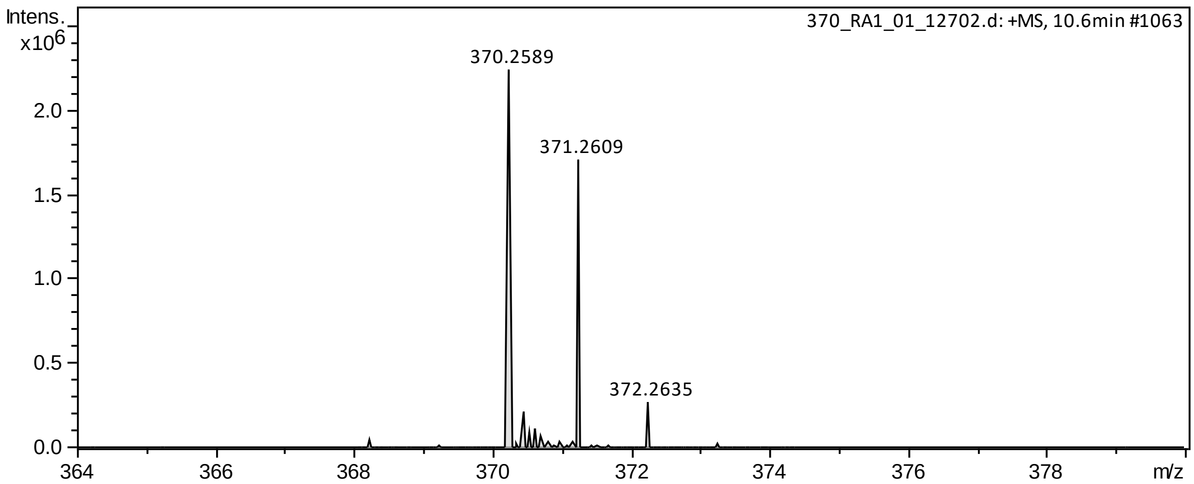

Figure S22. The HRESIMS of compound **4**.

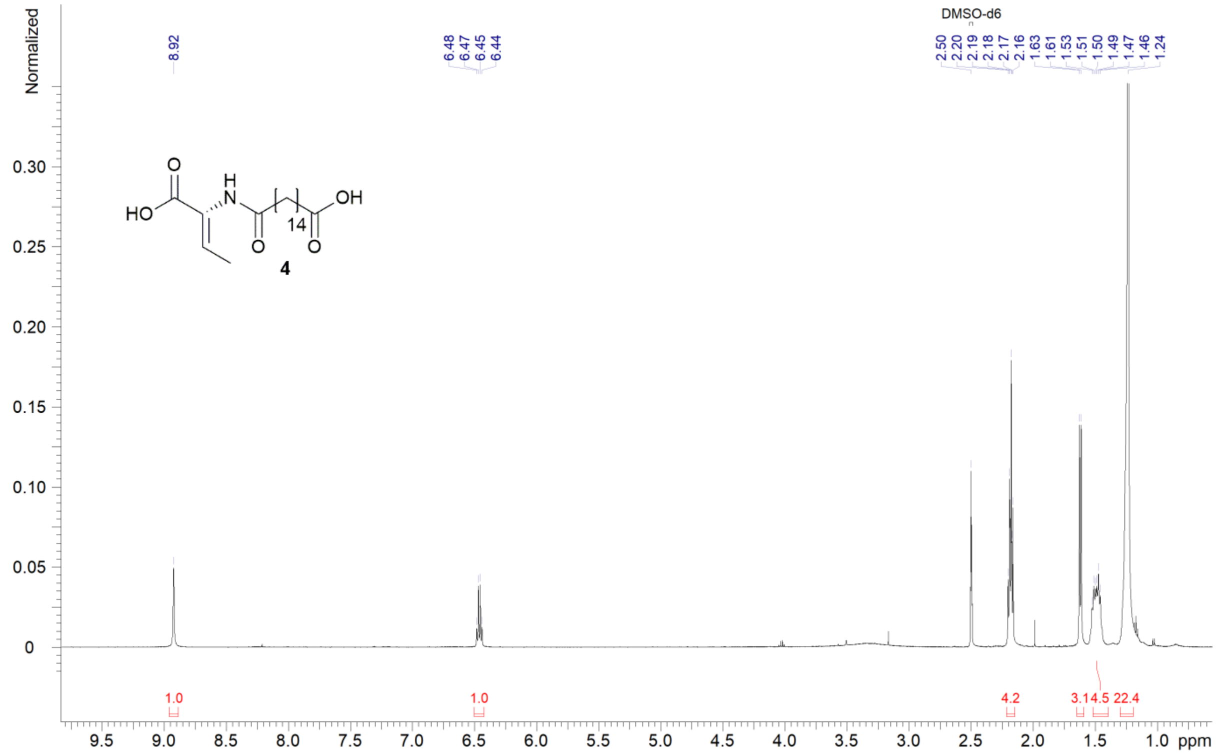

Figure S23: ^{1}H NMR spectrum (500 MHz, DMSO-d_6) of lipothrenin C (**4**).

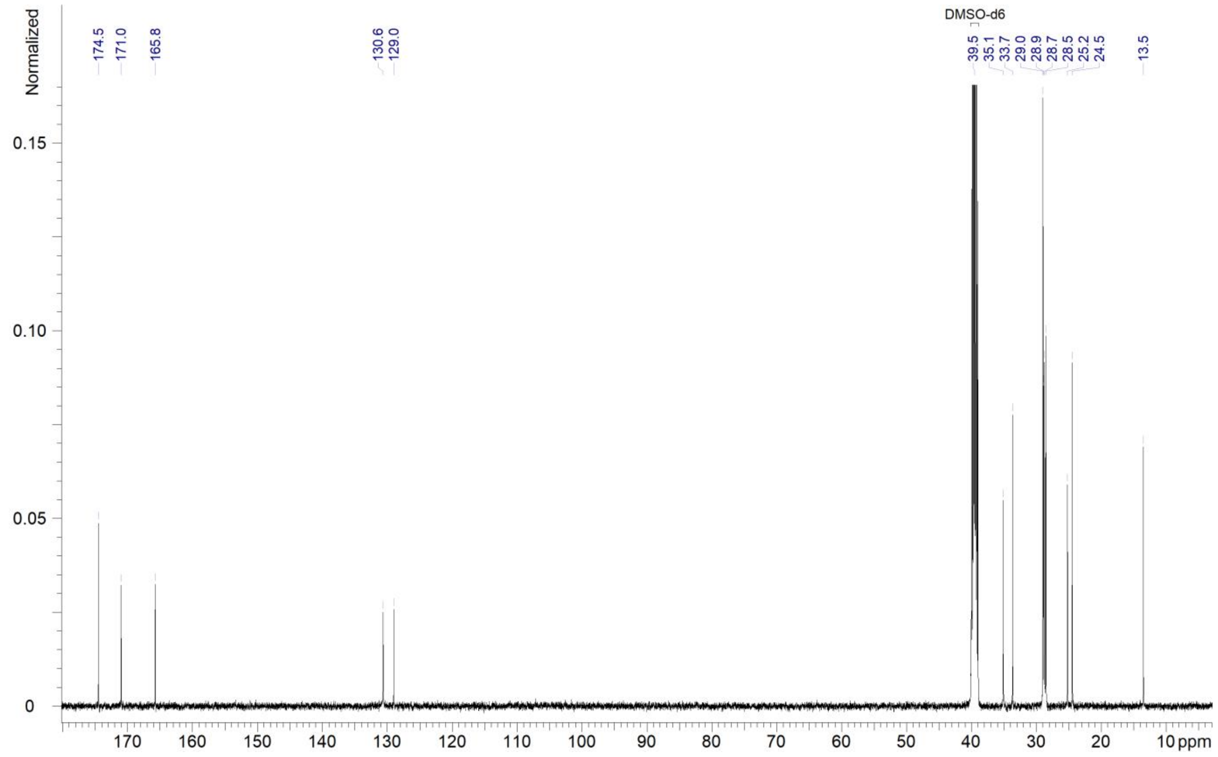

Figure S24: ^{13}C NMR spectrum (500 MHz, DMSO-d_6) of lipothrenin C (**4**).

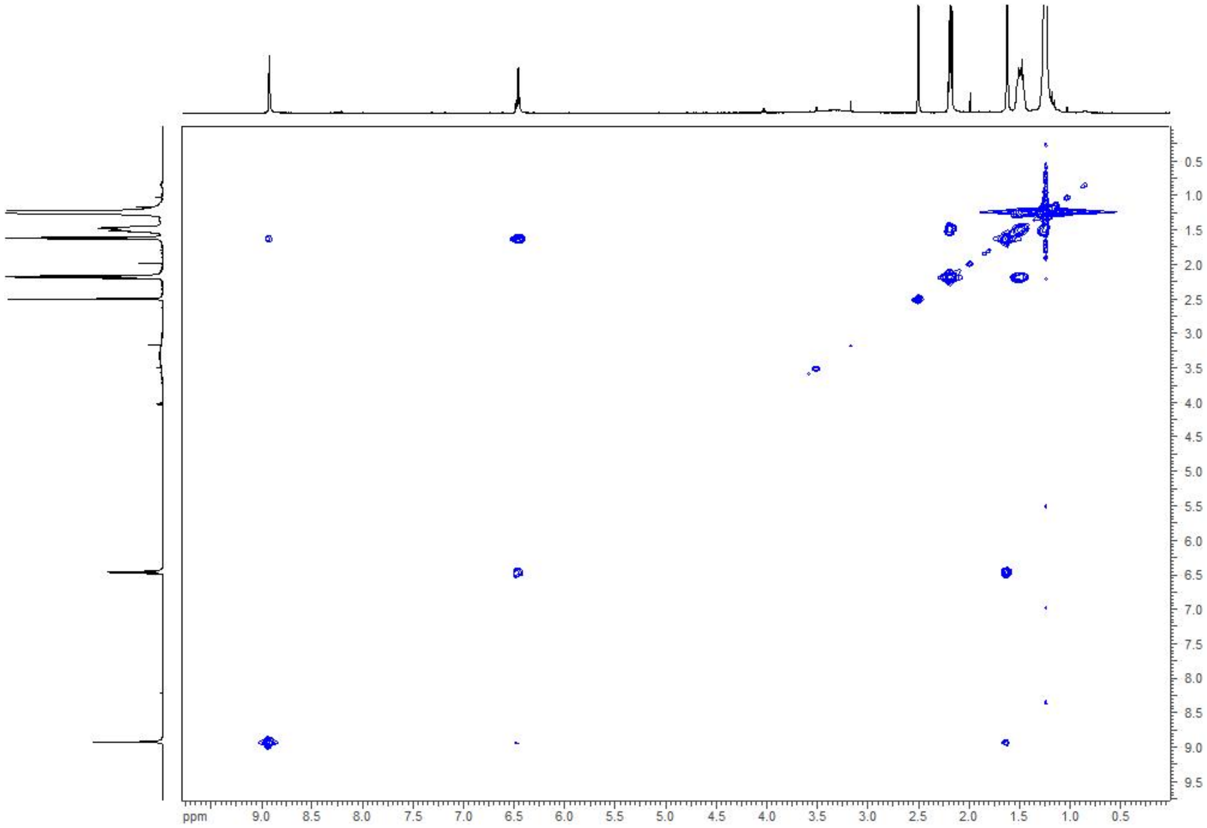

Figure S25: ^{1}H-^{1}H-COSY spectrum (500 MHz, DMSO-d_6) of lipothrenin C (**4**).

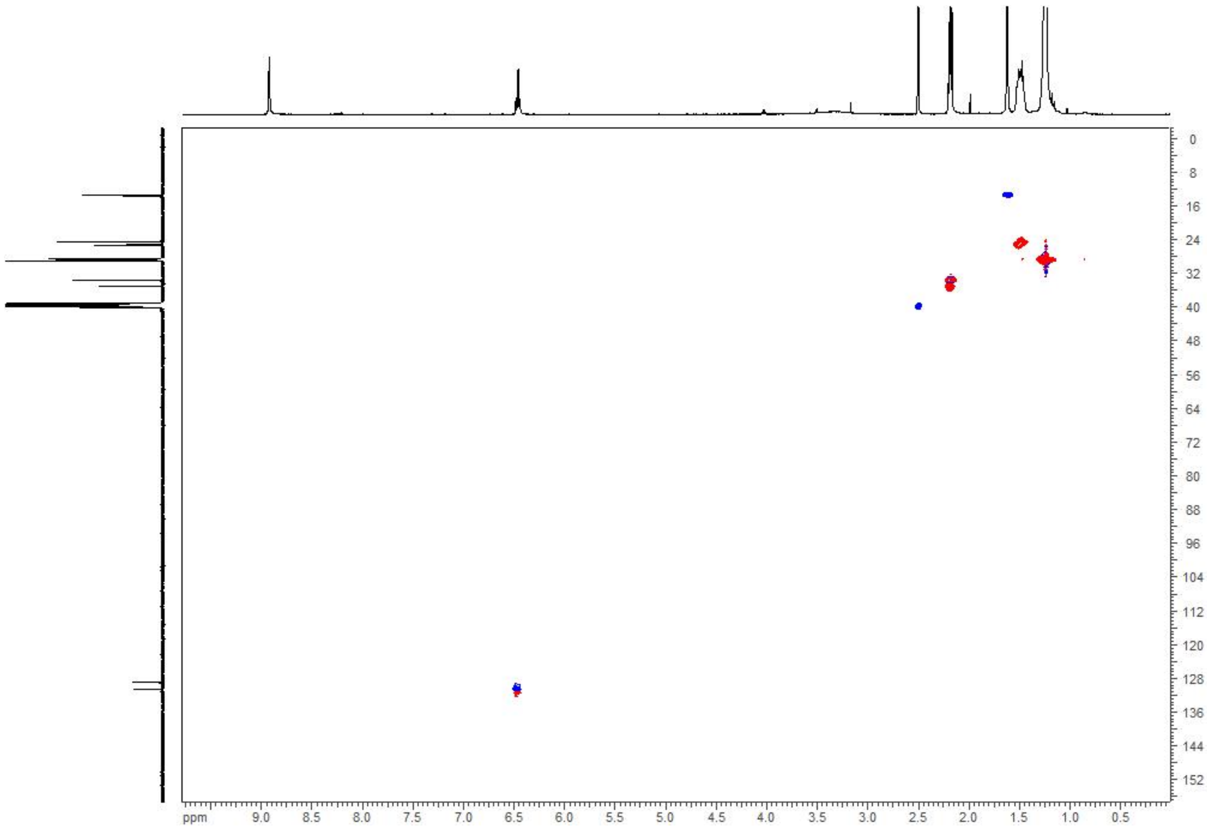

Figure S26: HSQC spectrum (500 MHz, DMSO-d_6) of lipothrenin C (**4**).

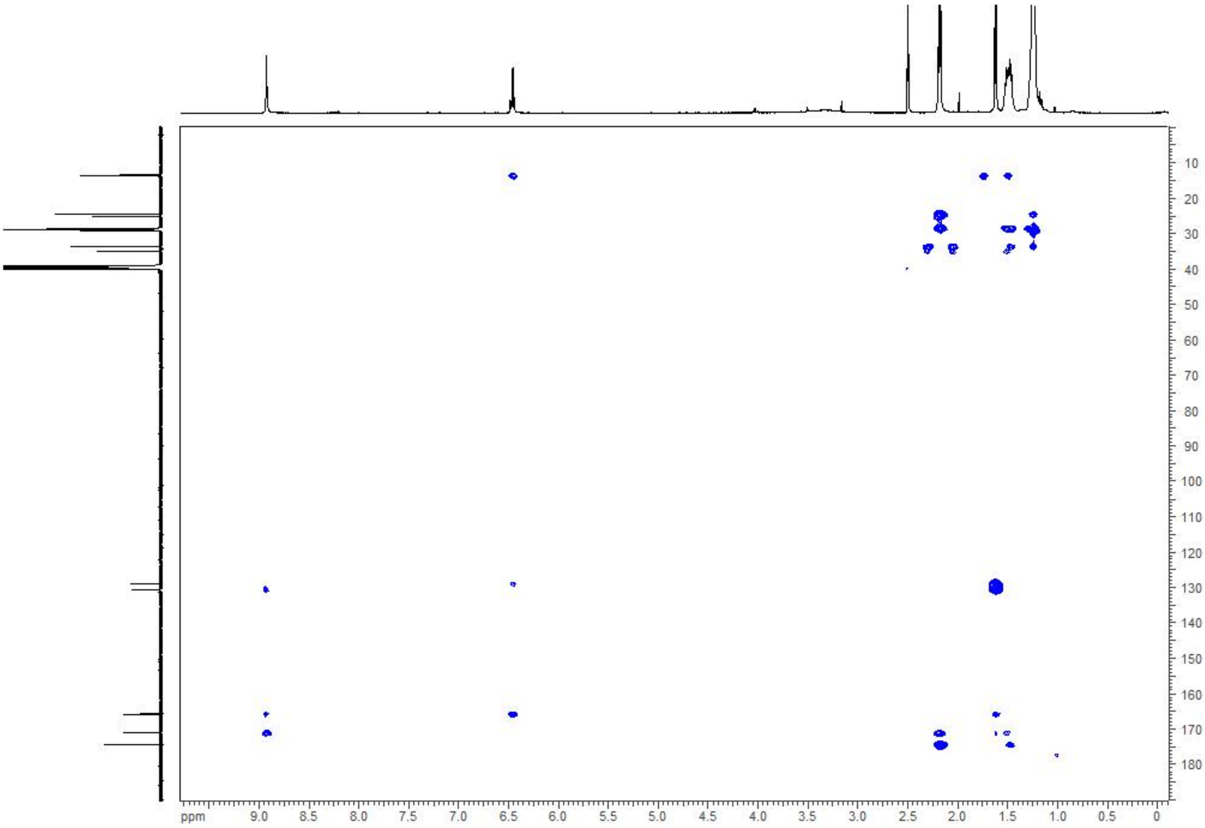

Figure S27: HMBC spectrum (500 MHz, DMSO-d_6) of lipothrenin C (**4**).

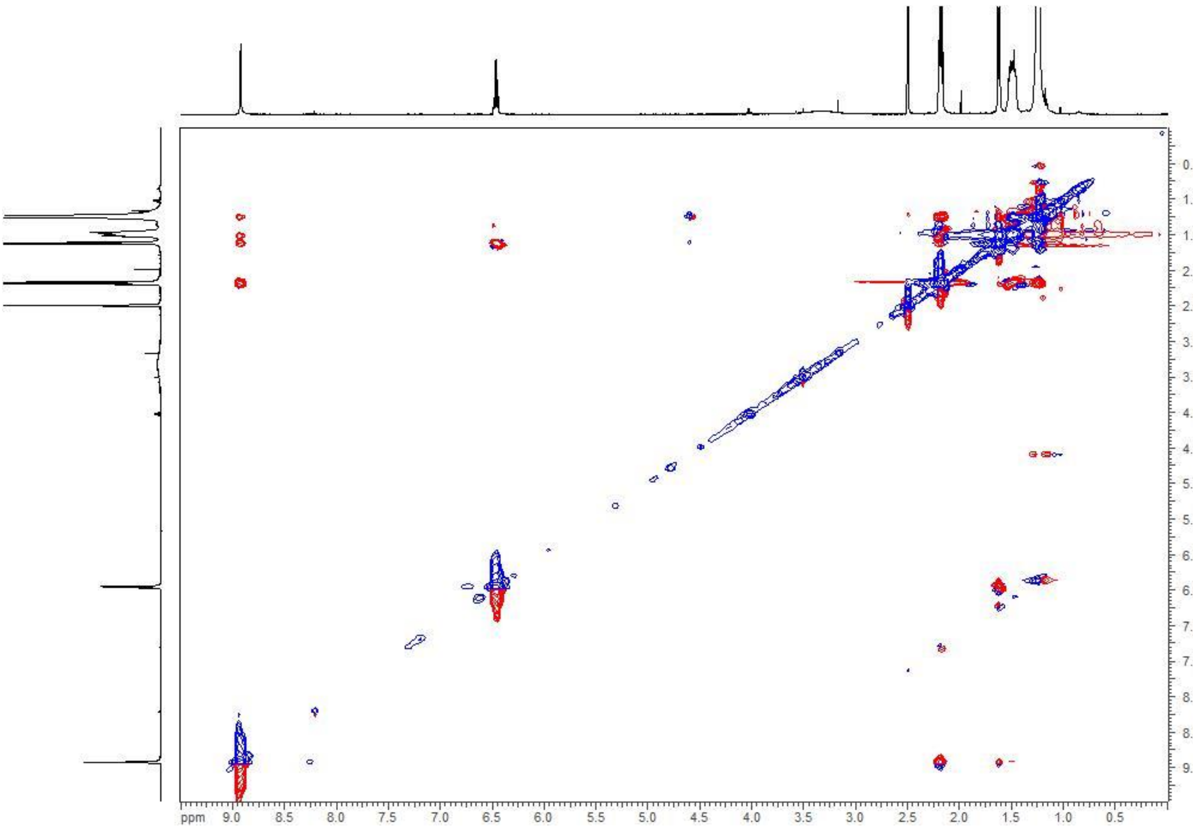

Figure S28: ROESY spectrum (500 MHz, DMSO-d_6) of lipothrenin C (**4**).

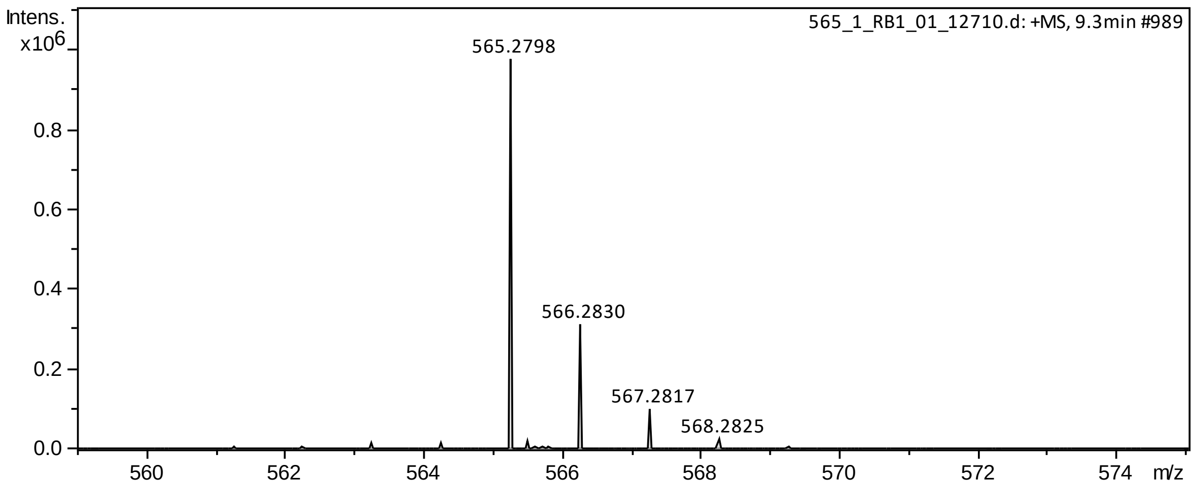

Figure S29. The HRESIMS of compound **5**.

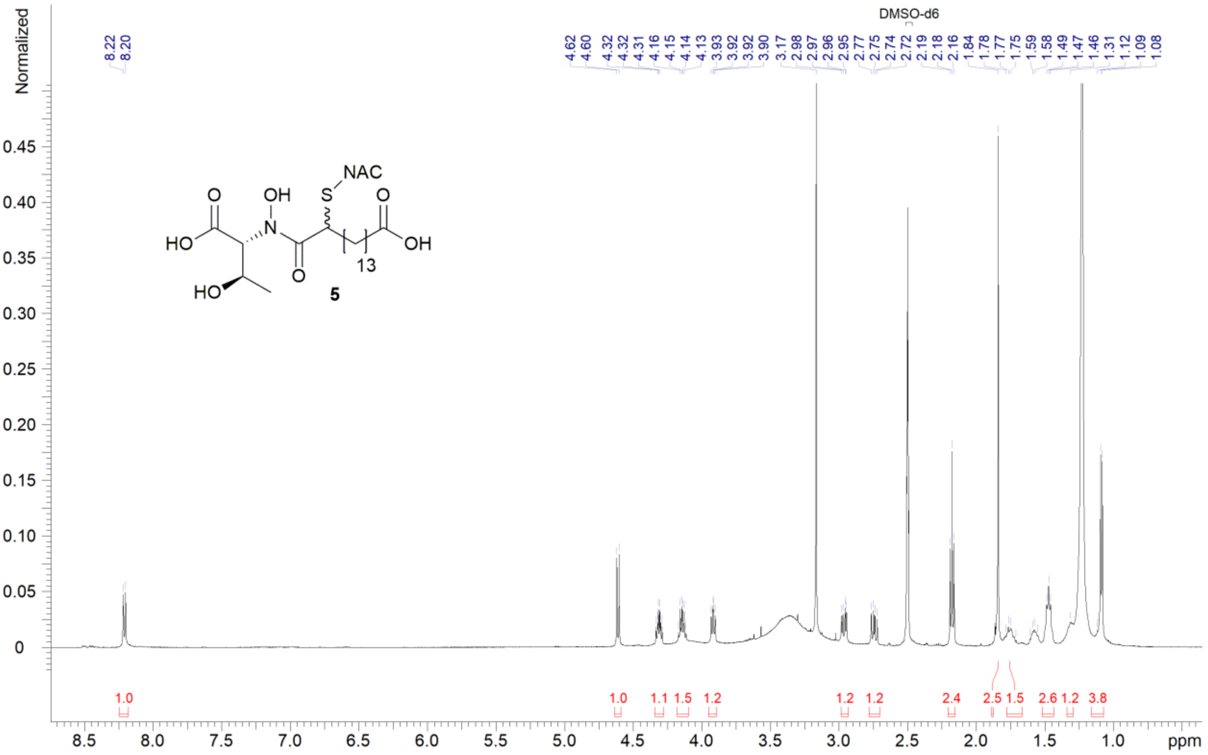

Figure S30: ^{1}H NMR spectrum (500 MHz, DMSO-d$_6$) of 2-NAC-lipothrenin A (**5**).

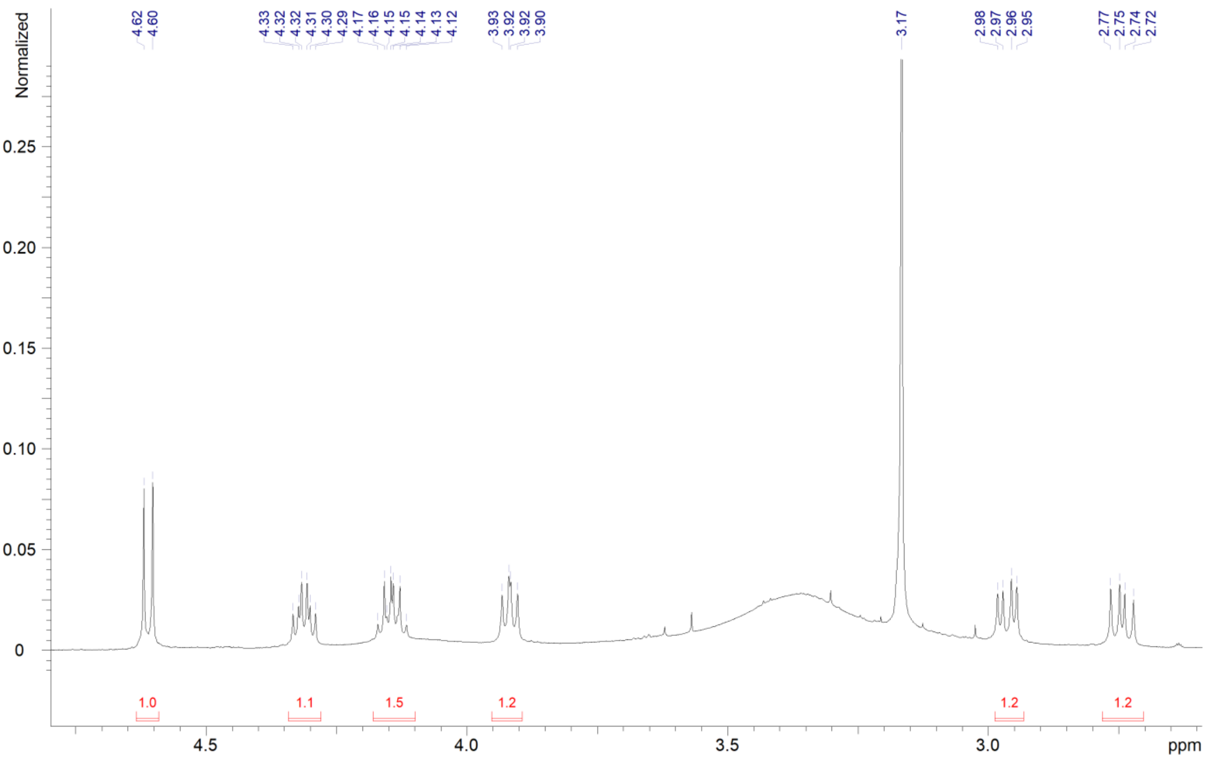

Figure S31. Zoomed in ^{1}H NMR spectrum (500 MHz, DMSO-d$_6$) of 2-NAC-lipothrenin A (**5**).

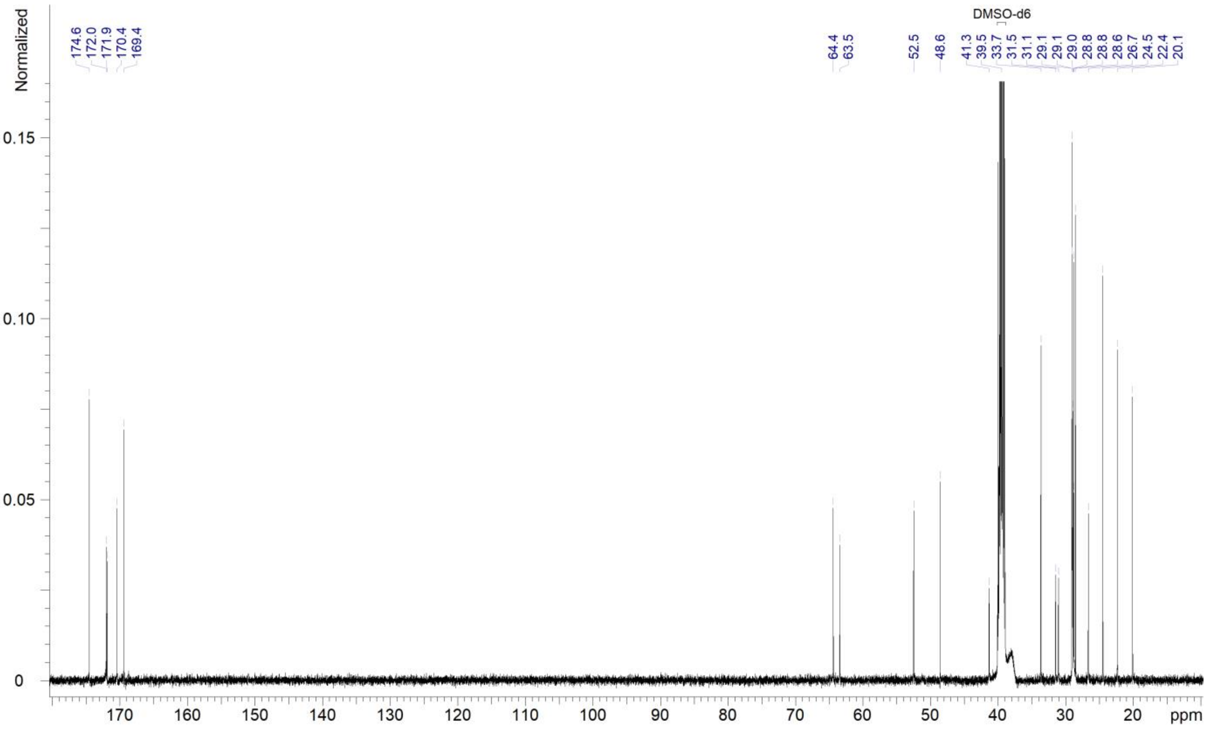

Figure S32: ^{13}C NMR spectrum (500 MHz, DMSO-d$_6$) of 2-NAC-lipothrenin A (**5**).

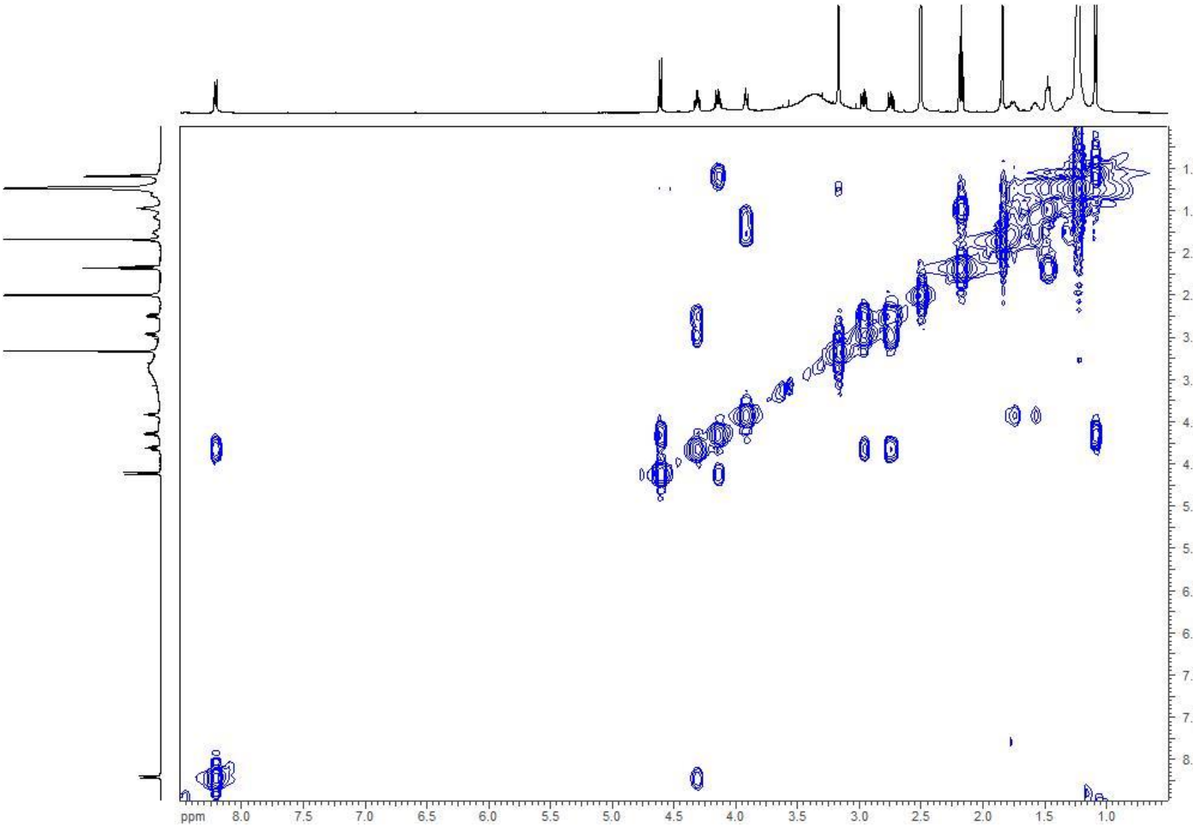

Figure S33: ^{1}H-^{1}H-COSY spectrum (500 MHz, DMSO-d$_6$) of 2-NAC-lipothrenin A (**5**).

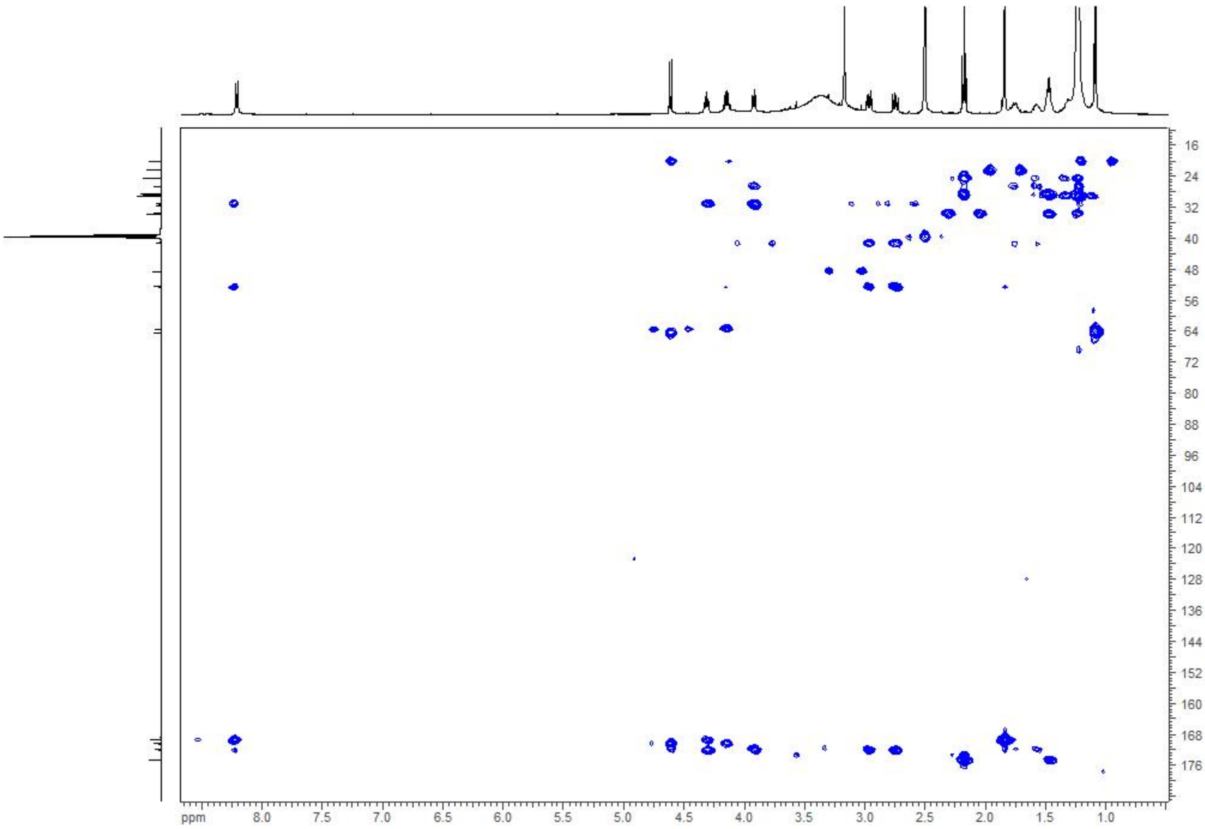

Figure S34: HMBC spectrum (500 MHz, DMSO-d$_6$) of 2-NAC-lipothrenin A (**5**).

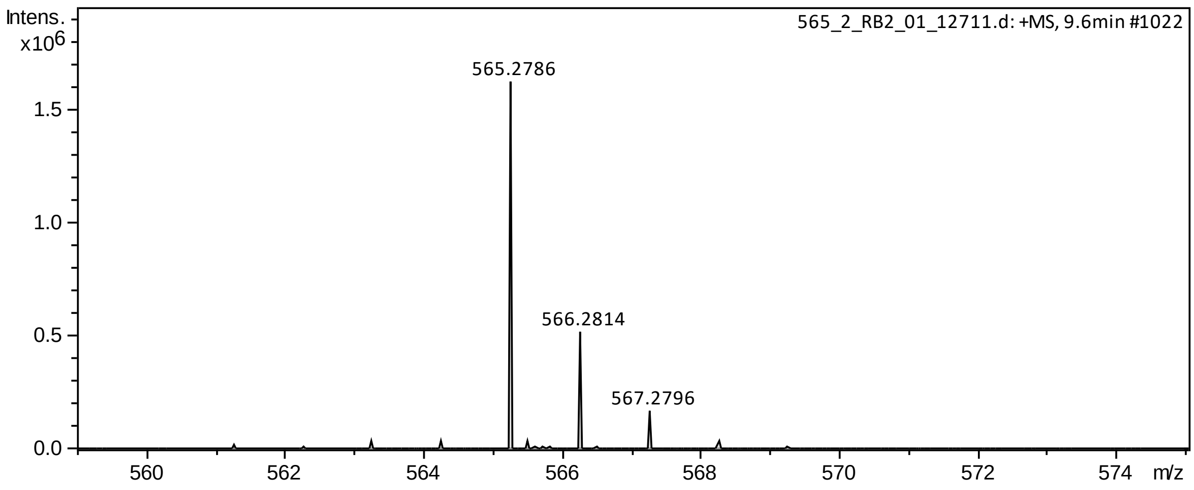

Figure S35. The HRESIMS of compound **6**.

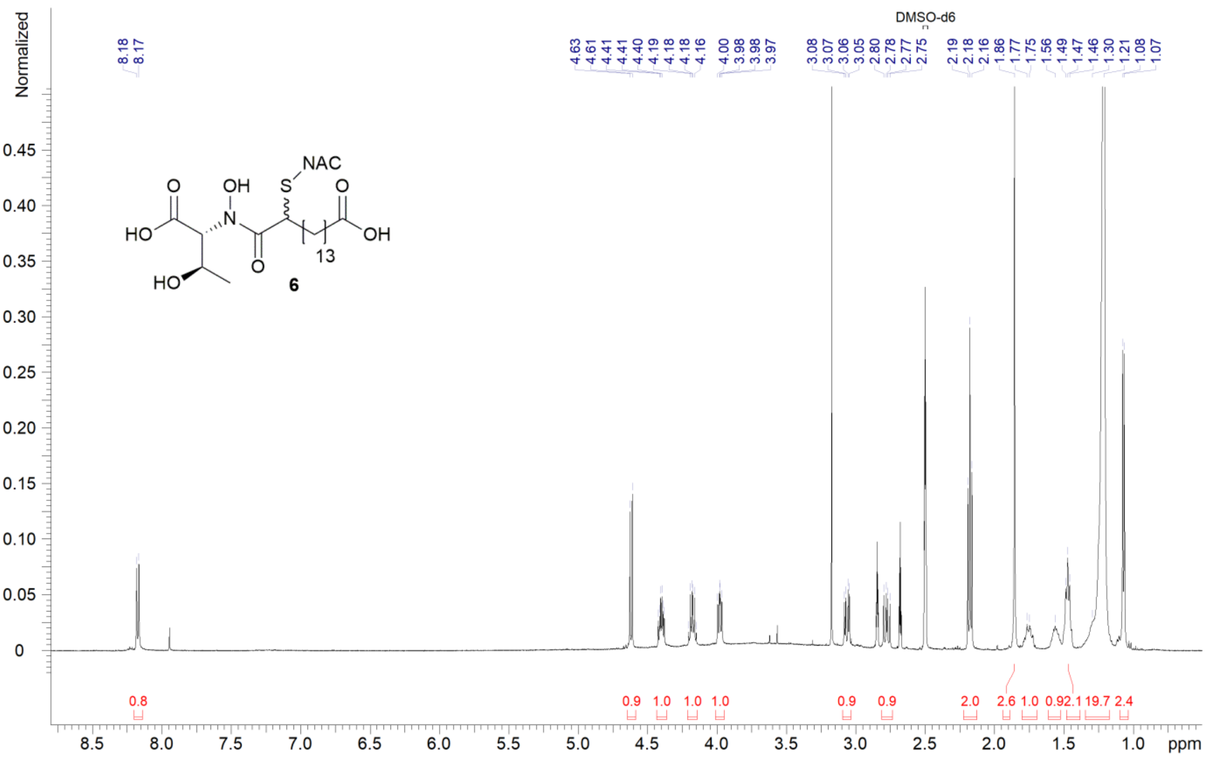

Figure S36: ^{1}H NMR spectrum (500 MHz, DMSO-d_6) of 2-NAC-lipothrenin A_1 (**6**).

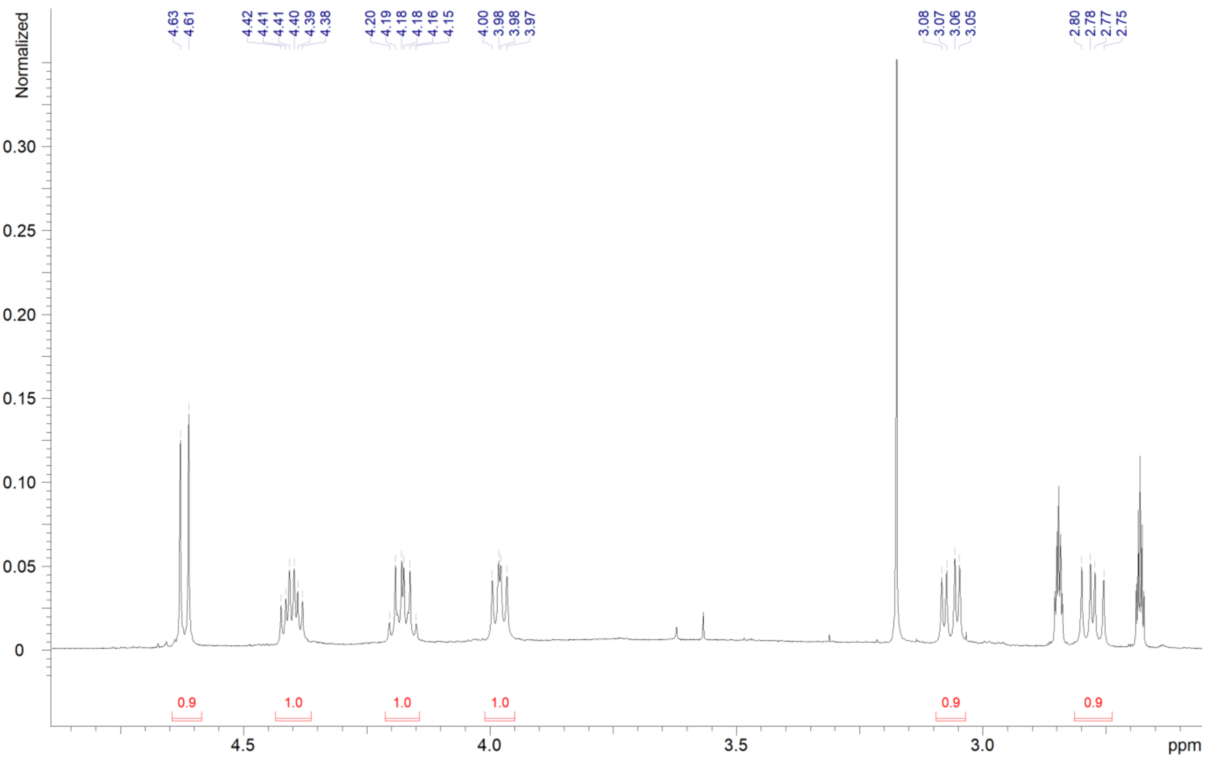

Figure S37. Zoomed in ^{1}H NMR spectrum (500 MHz, DMSO-d$_6$) of 2-NAC-lipothrenin A$_1$ (**6**).

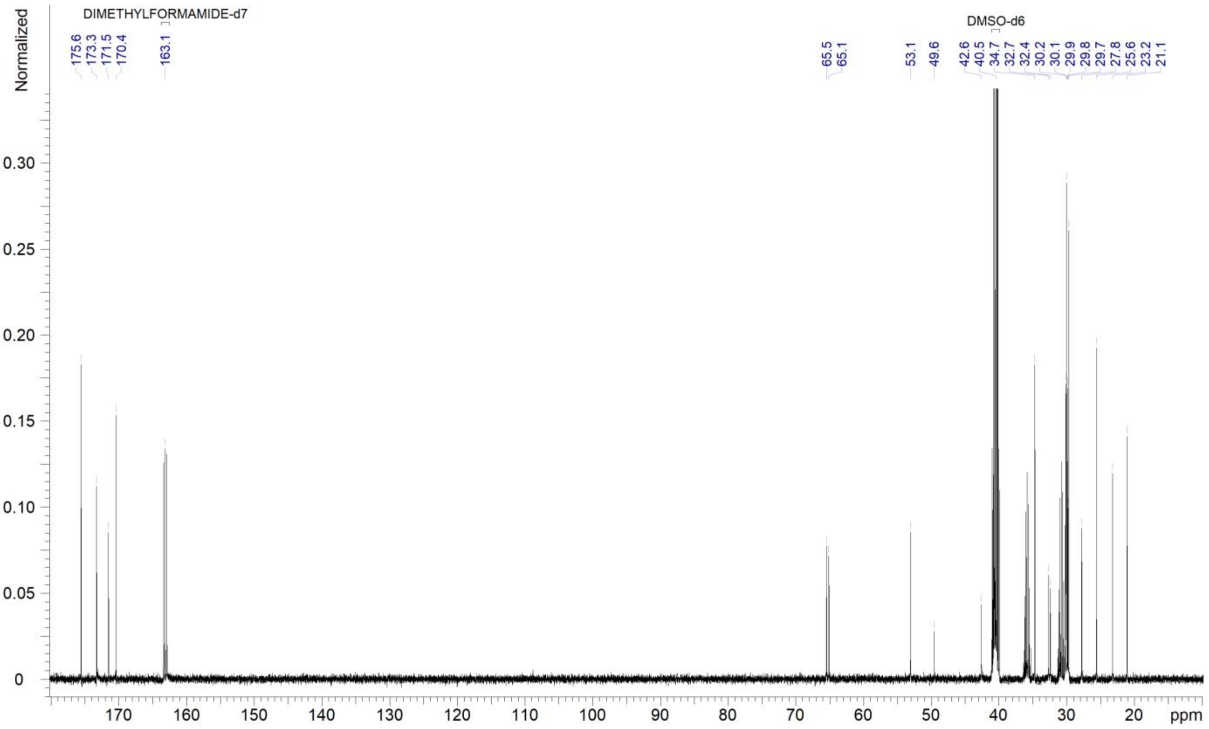

Figure S38: ^{13}C NMR spectrum (500 MHz, DMSO-d$_6$) of 2-NAC-lipothrenin A$_1$ (**6**).

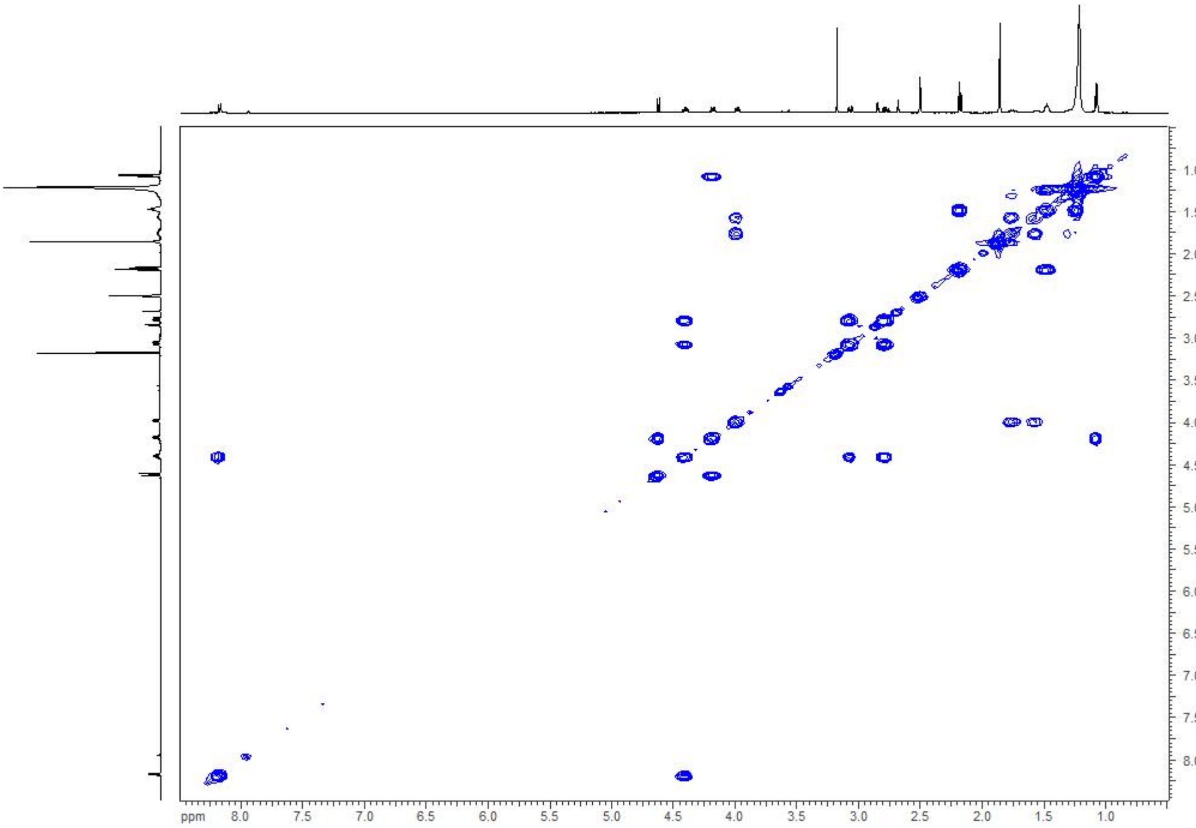

Figure S39: ^1H-^1H-COSY spectrum (500 MHz, DMSO-d_6) of 2-NAC-lipothrenin A_1 (**6**).

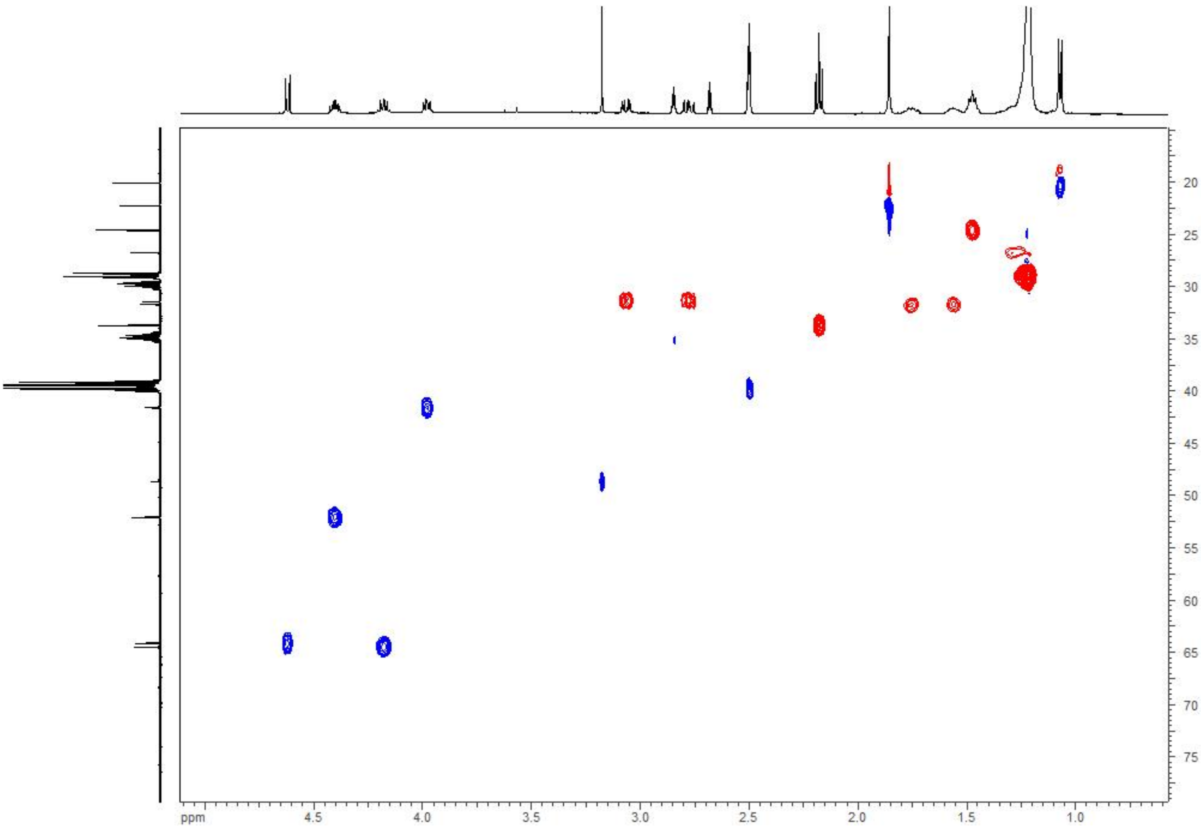

Figure S40: HSQC spectrum (500 MHz, DMSO-d_6) of 2-NAC-lipothrenin A_1 (**6**).

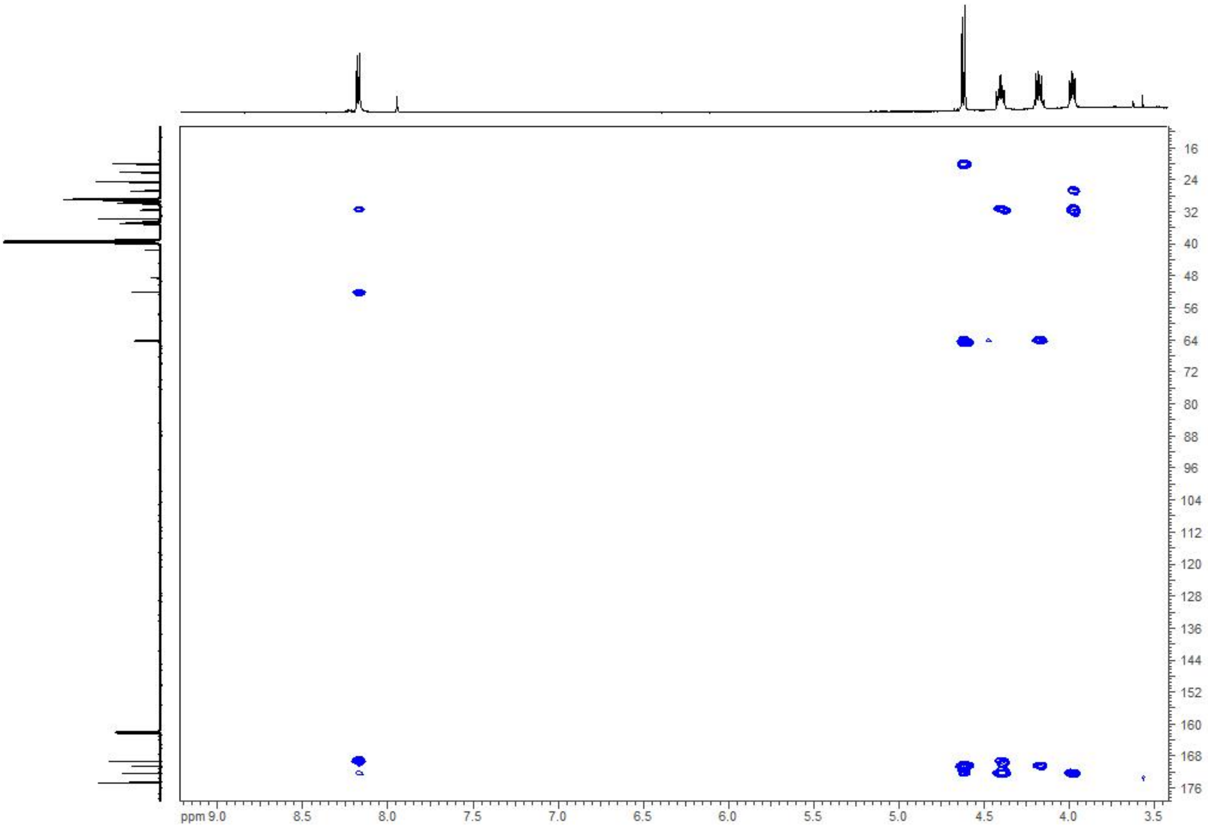

Figure S41: HMBC spectrum (500 MHz, DMSO-d_6) of 2-NAC-lipothrenin A_1 (**6**).

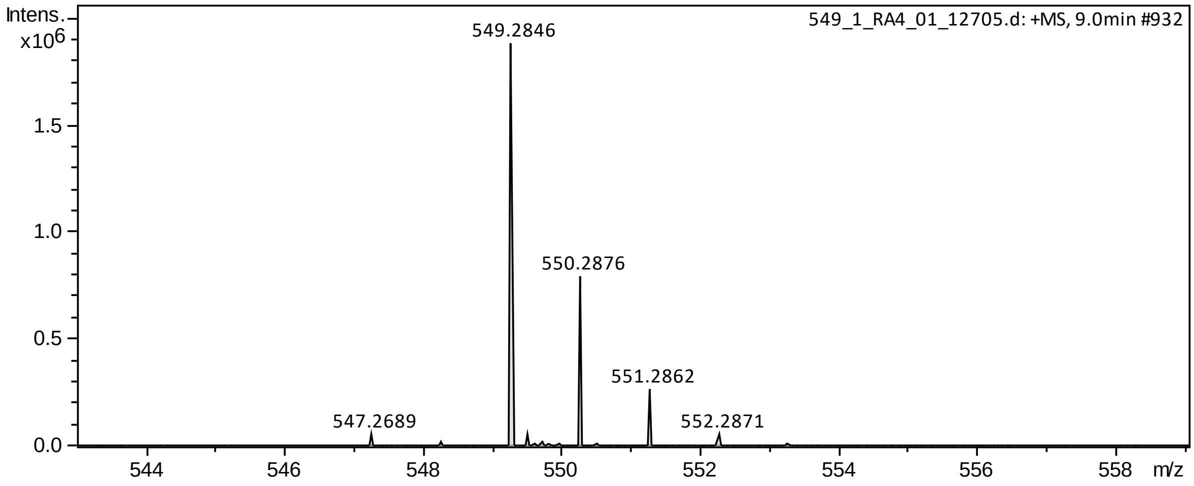

Figure S42. The HRESIMS of compound **7**.

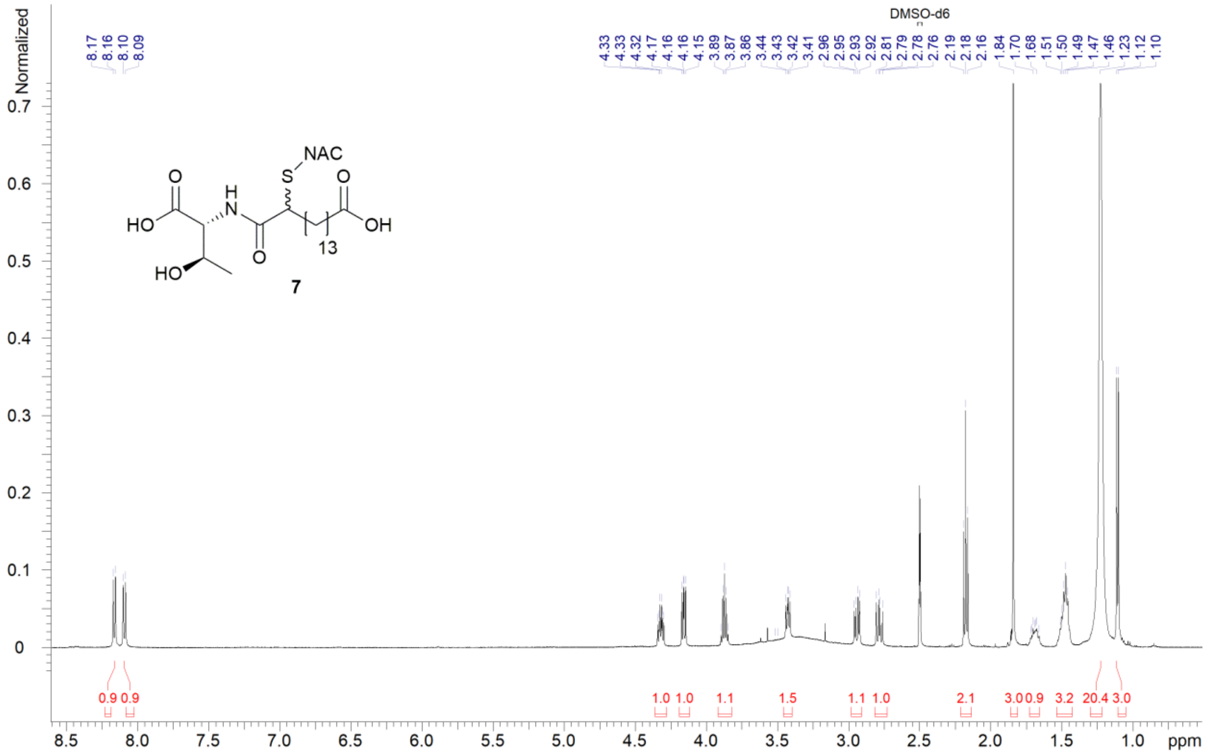

Figure S43: ^{1}H NMR spectrum (500 MHz, DMSO-d_6) of 2-NAC-D-lipothrenin B (**7**).

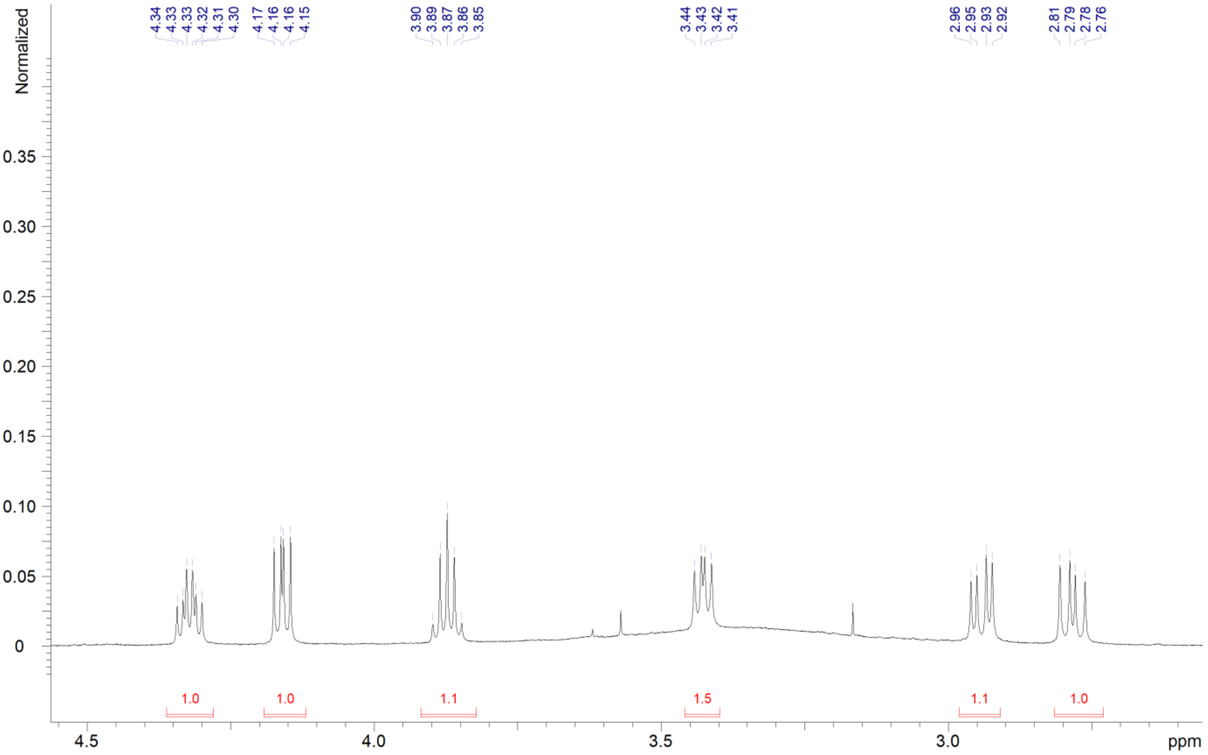

Figure S44. Zoomed in ^{1}H NMR spectrum (500 MHz, DMSO-d_6) of 2-NAC-D-lipothrenin B (**7**).

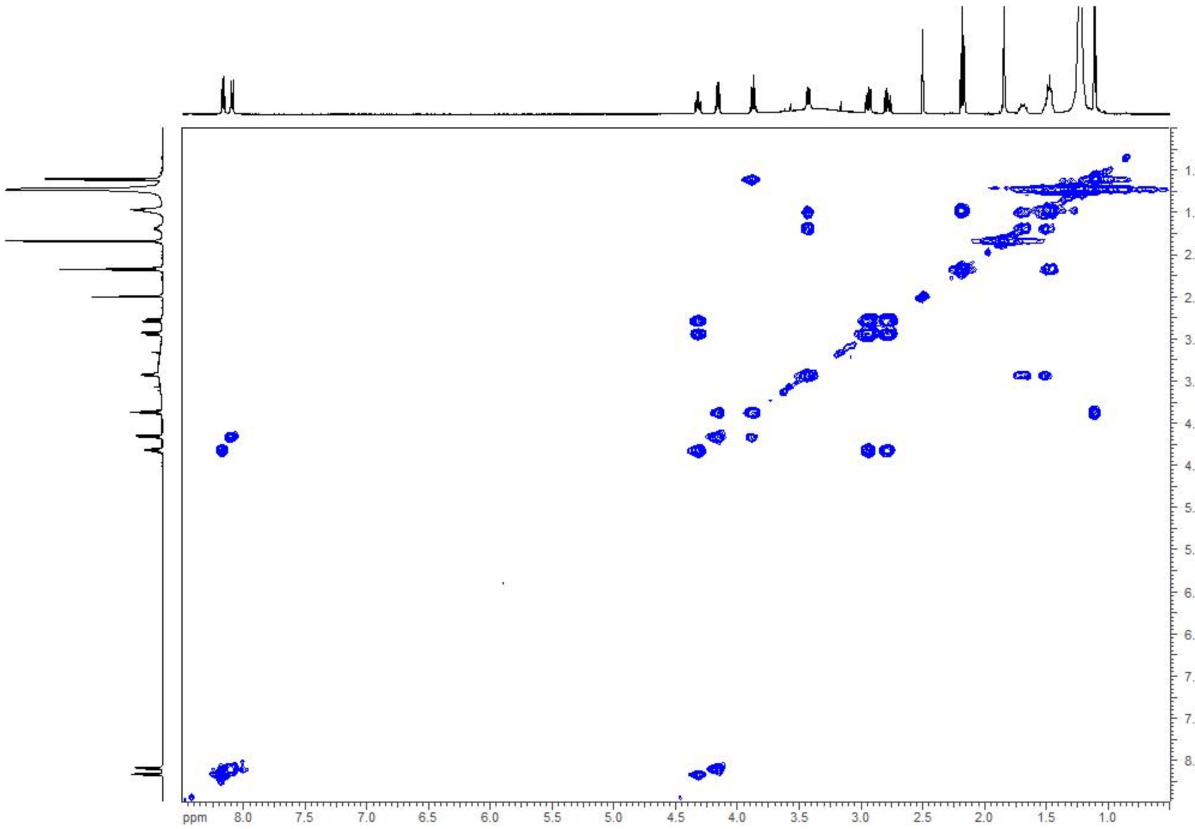

Figure S45: ^{1}H-^{1}H-COSY spectrum (500 MHz, DMSO-d$_6$) of 2-NAC-D-lipothrenin B (**7**).

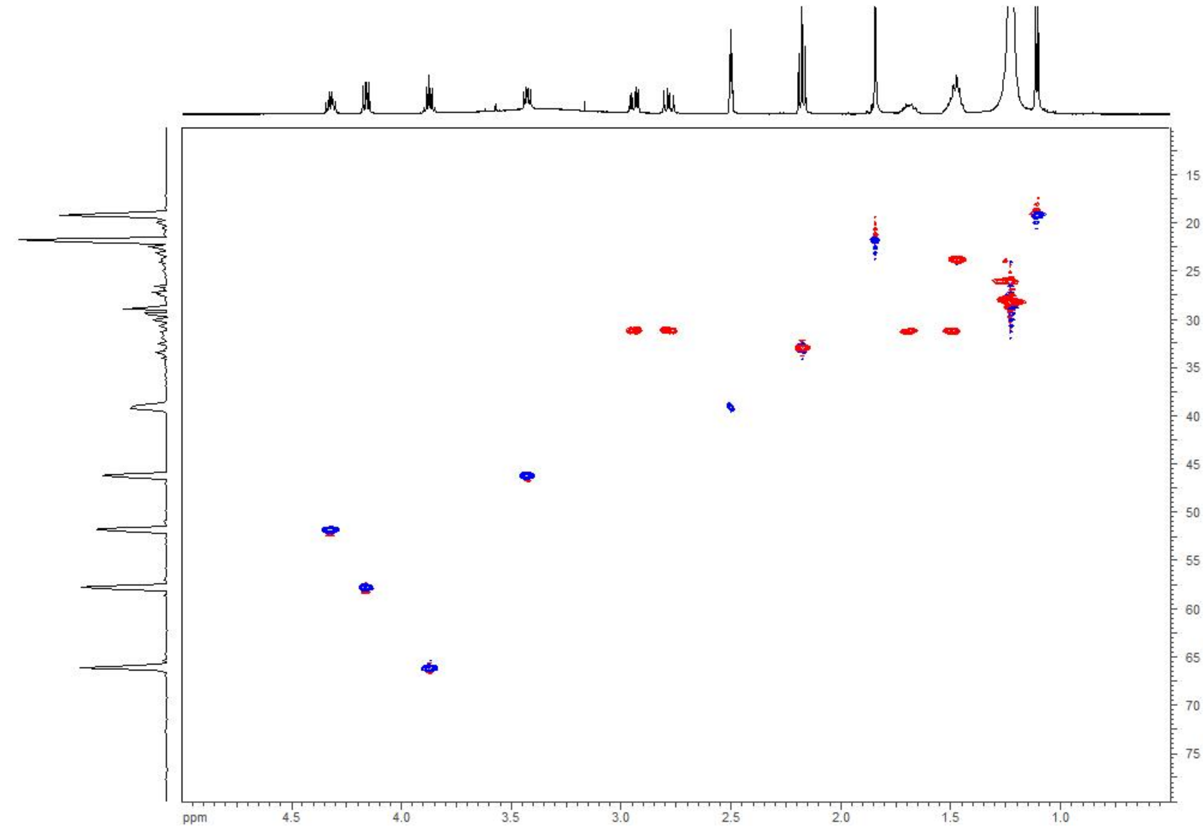

Figure S46: HSQC spectrum (500 MHz, DMSO-d$_6$) of 2-NAC-D-lipothrenin B (**7**).

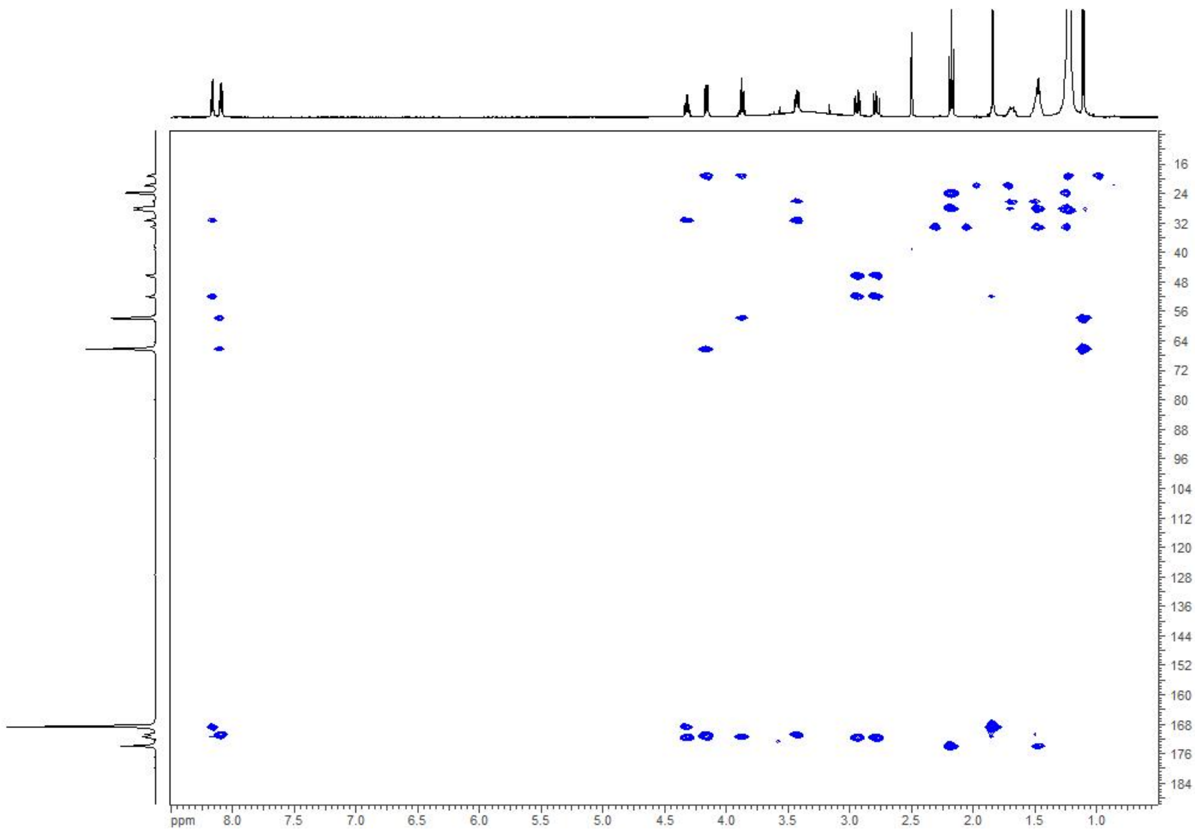

Figure S47: HMBC spectrum (500 MHz, DMSO-d$_6$) of 2-NAC-D-lipothrenin B (**7**).

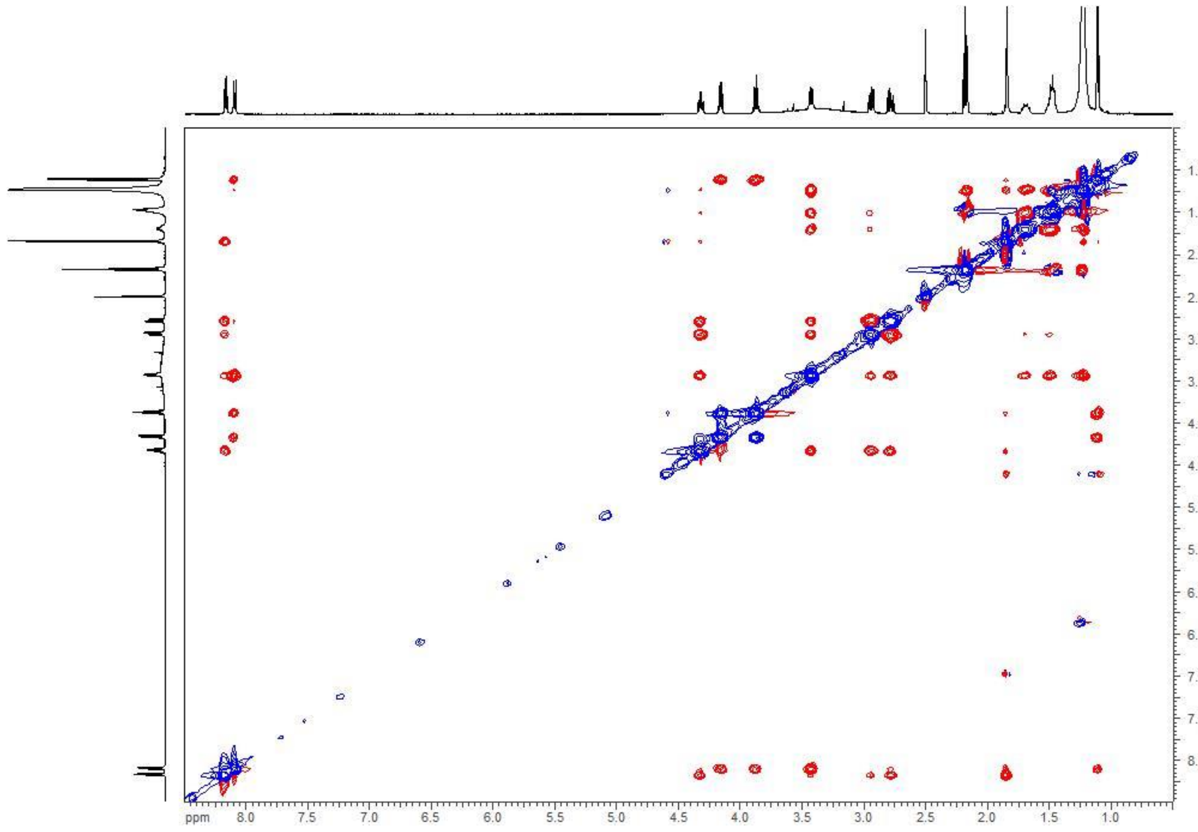

Figure S48: ROESY spectrum (500 MHz, DMSO-d$_6$) of 2-NAC-D-lipothrenin B (**7**).

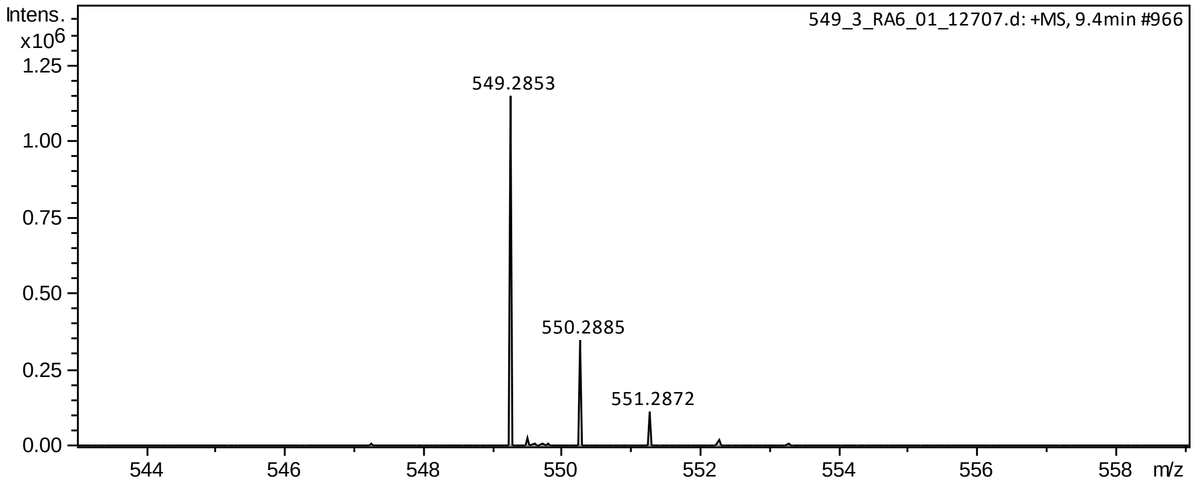

Figure S49. The HRESIMS of compound **8**.

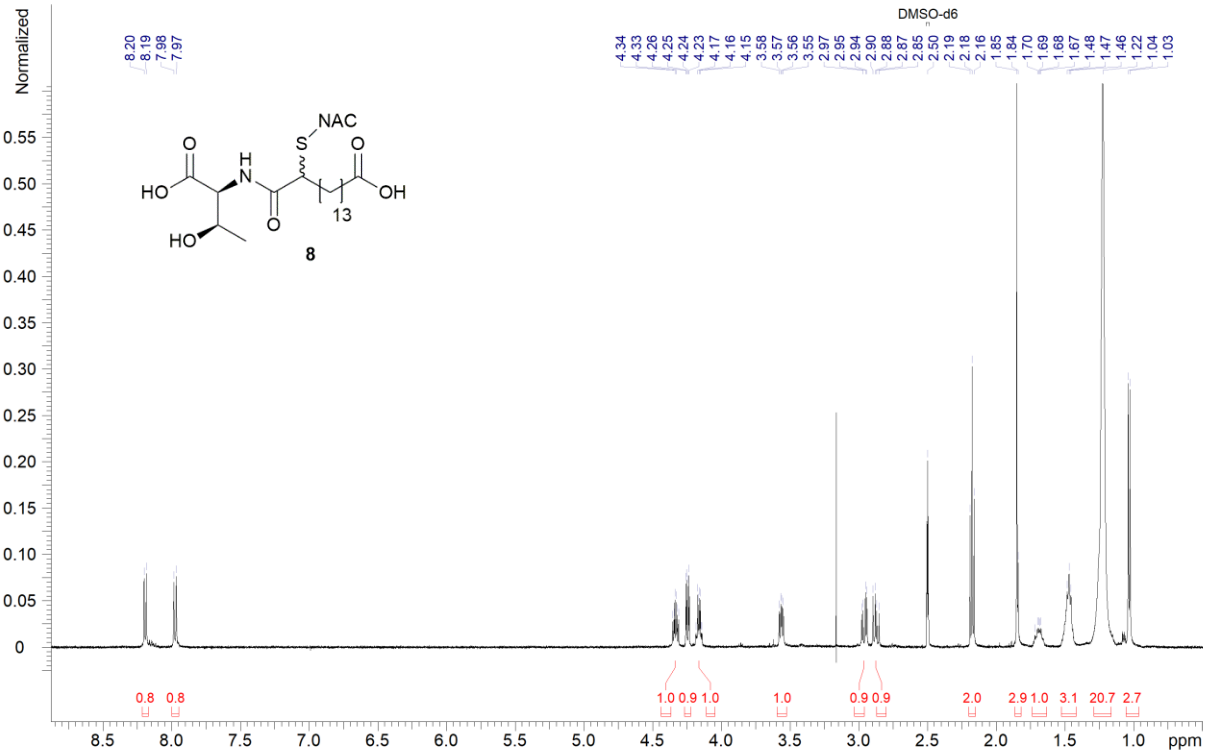

Figure S50: ^{1}H NMR spectrum (500 MHz, DMSO-d_6) of 2-NAC-L-lipothrenin B (**8**).

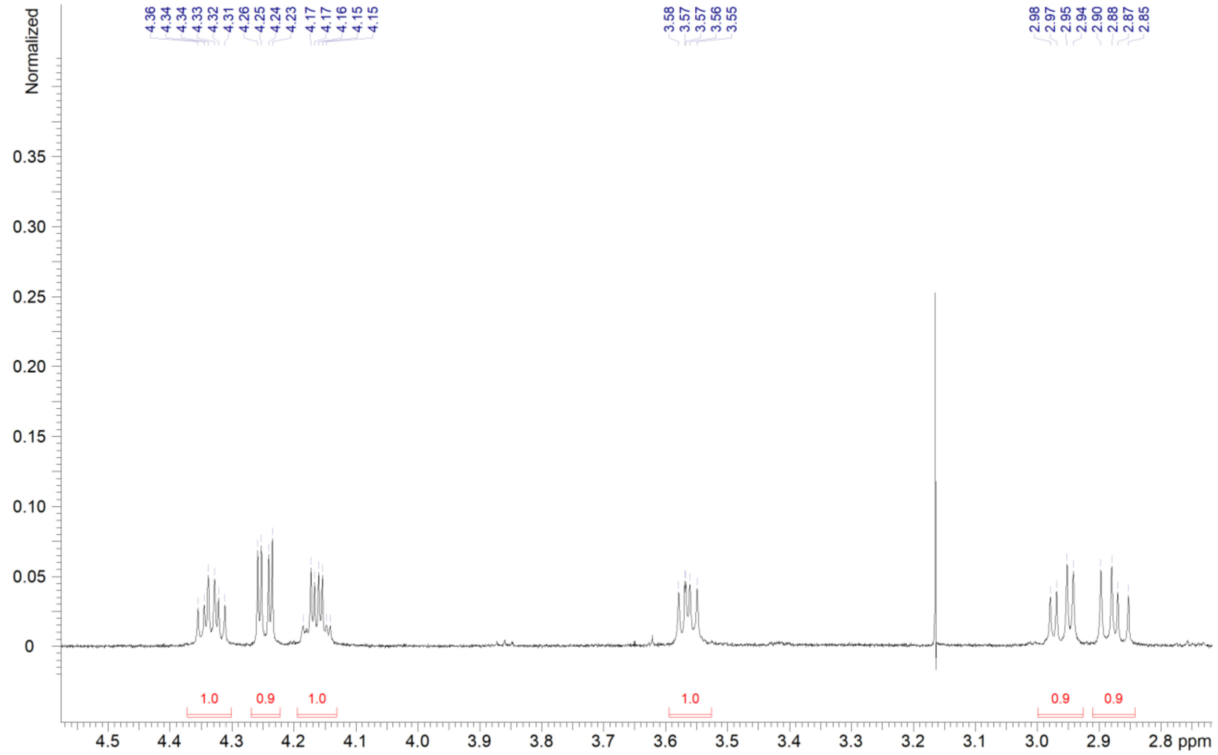

Figure S51. Zoomed in ^{1}H NMR spectrum (500 MHz, DMSO-d_6) of 2-NAC-L-lipothrenin B (**8**).

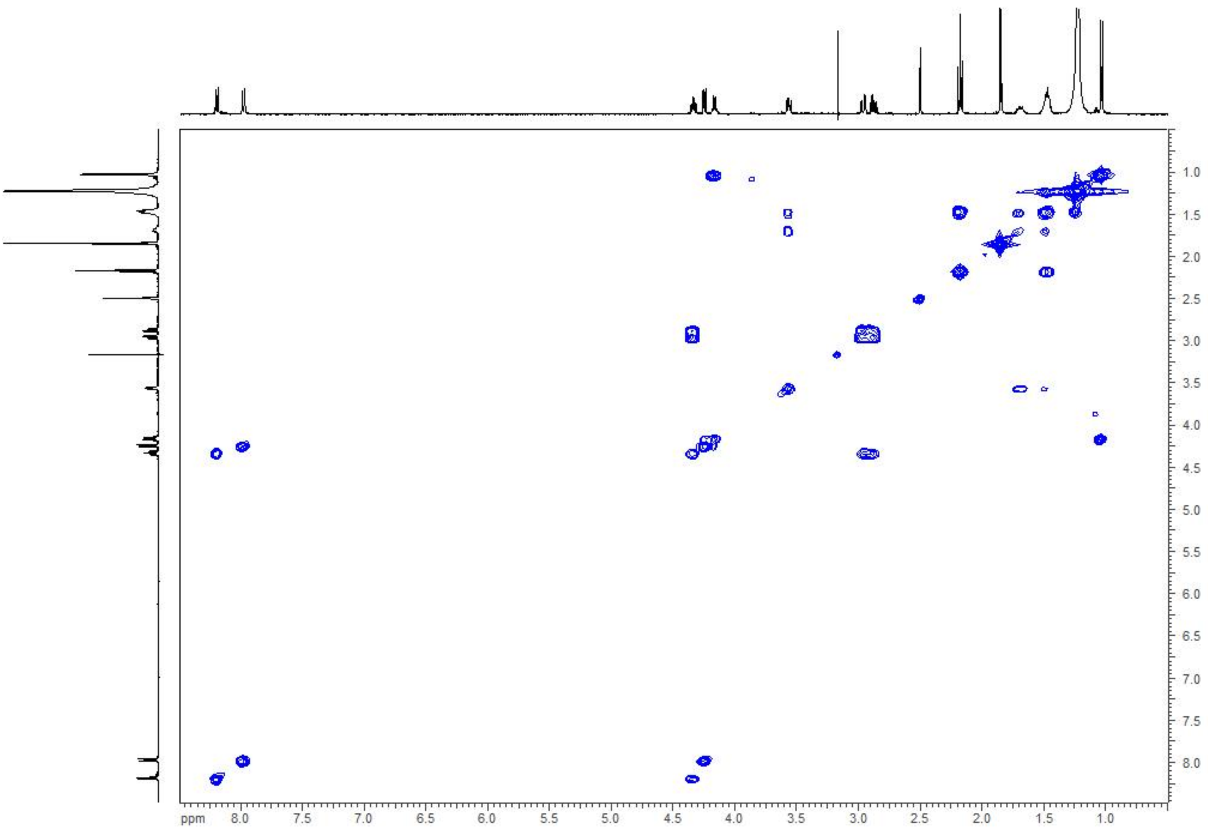

Figure S52: ^{1}H-^{1}H-COSY spectrum (500 MHz, DMSO-d_6) of 2-NAC-L-lipothrenin B (**8**).

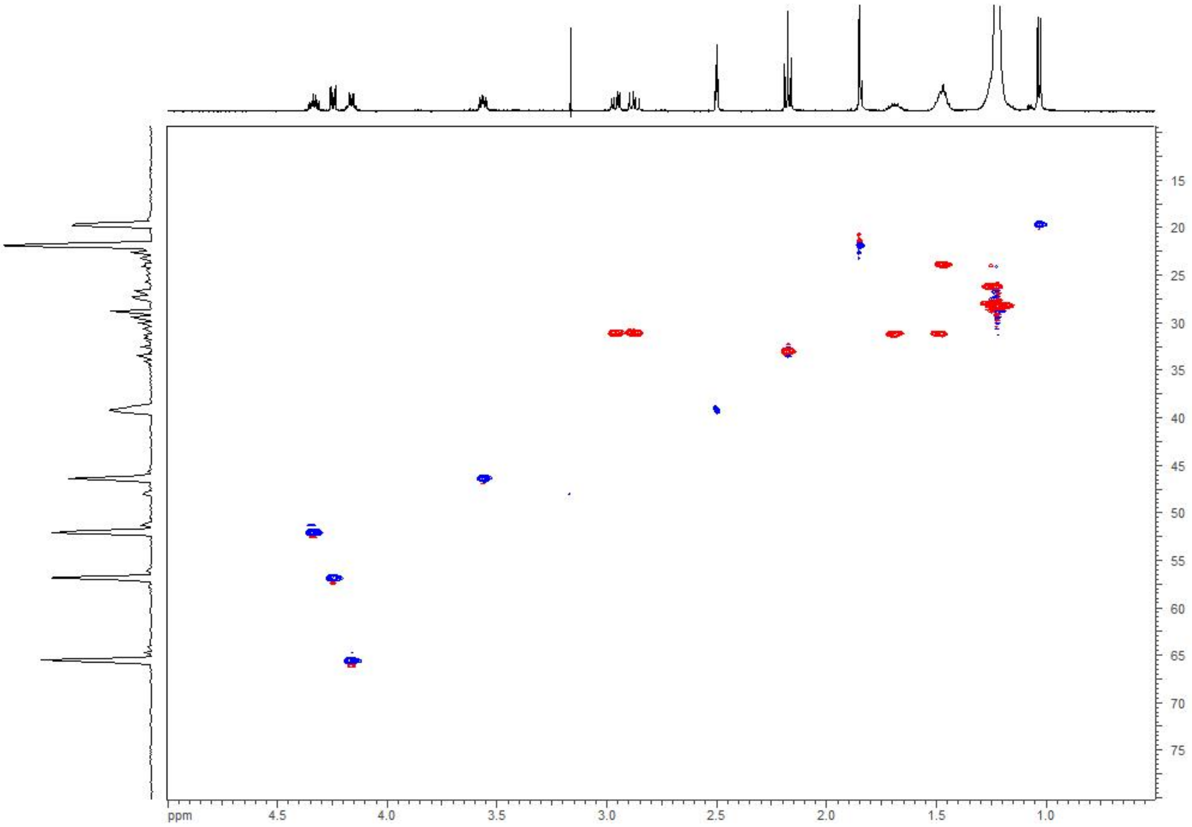

Figure S53: HSQC spectrum (500 MHz, DMSO-d_6) of 2-NAC-L-lipothrenin B (**8**).

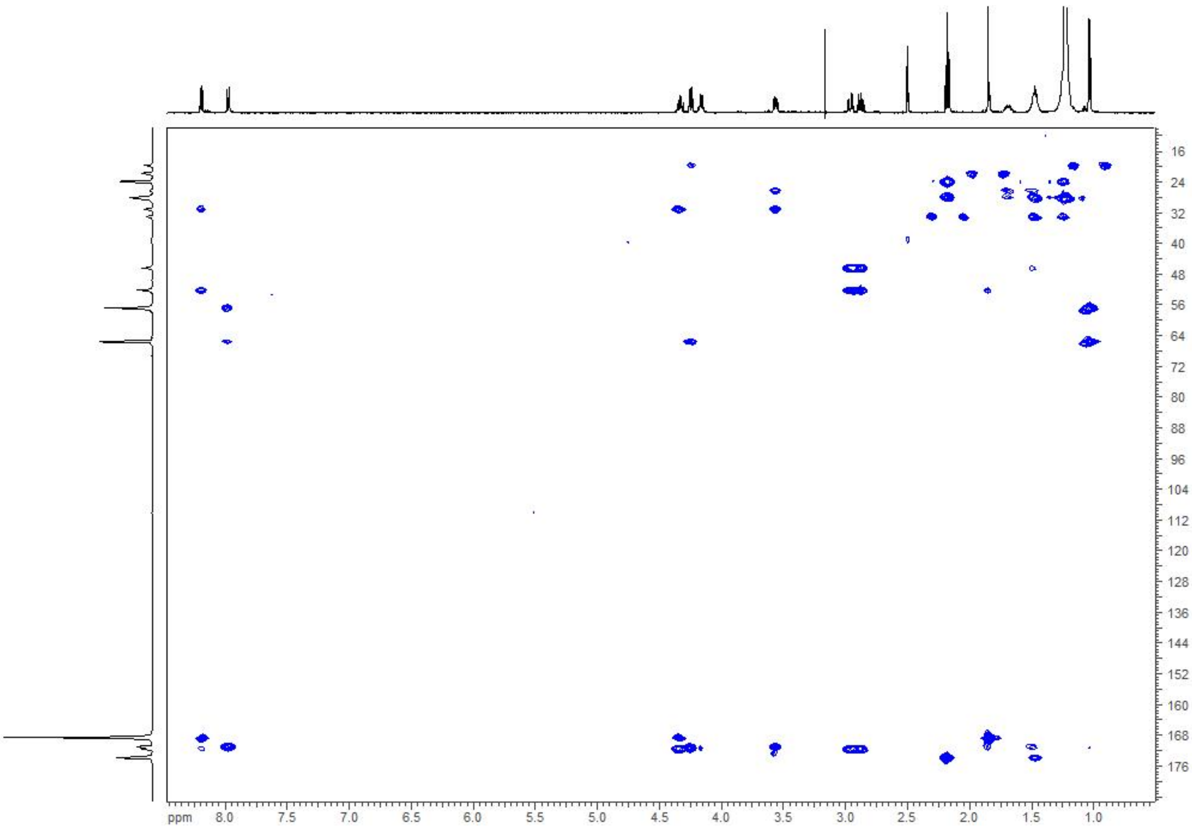

Figure S54: HMBC spectrum (500 MHz, DMSO-d_6) of 2-NAC-L-lipothrenin B (**8**).

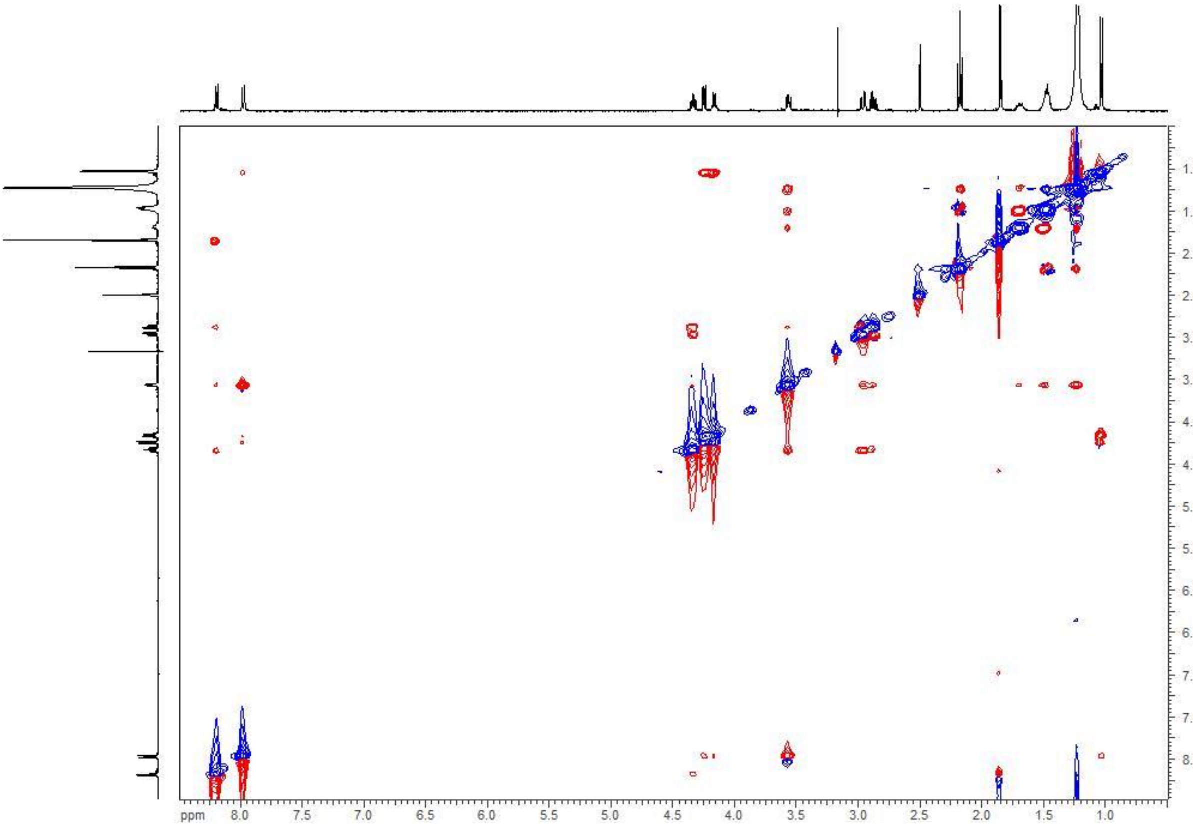

Figure S55: ROESY spectrum (500 MHz, DMSO-d$_6$) of 2-NAC-L-lipothrenin B (**8**).

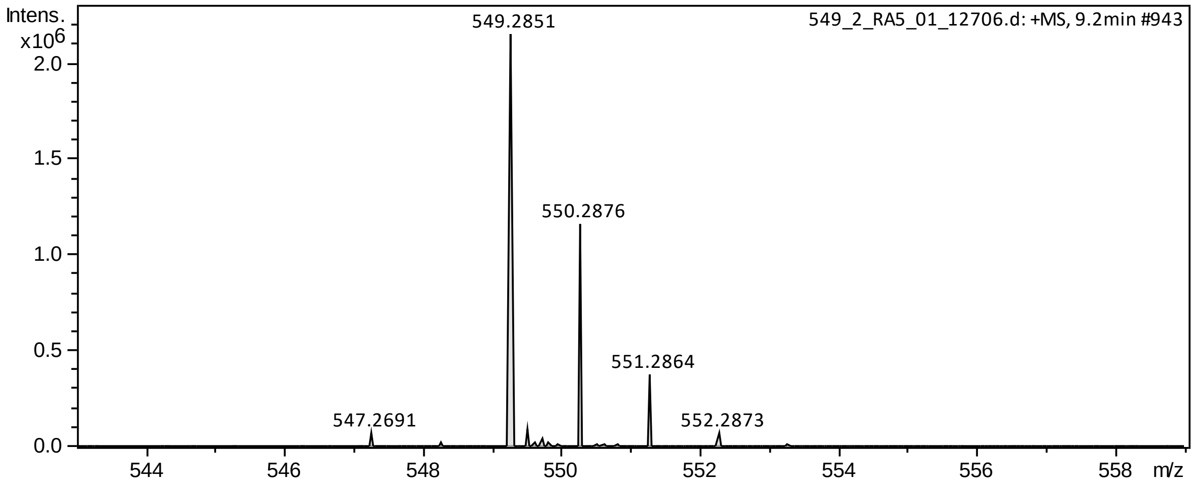

Figure S56. The HRESIMS of compound **9**, **10**.

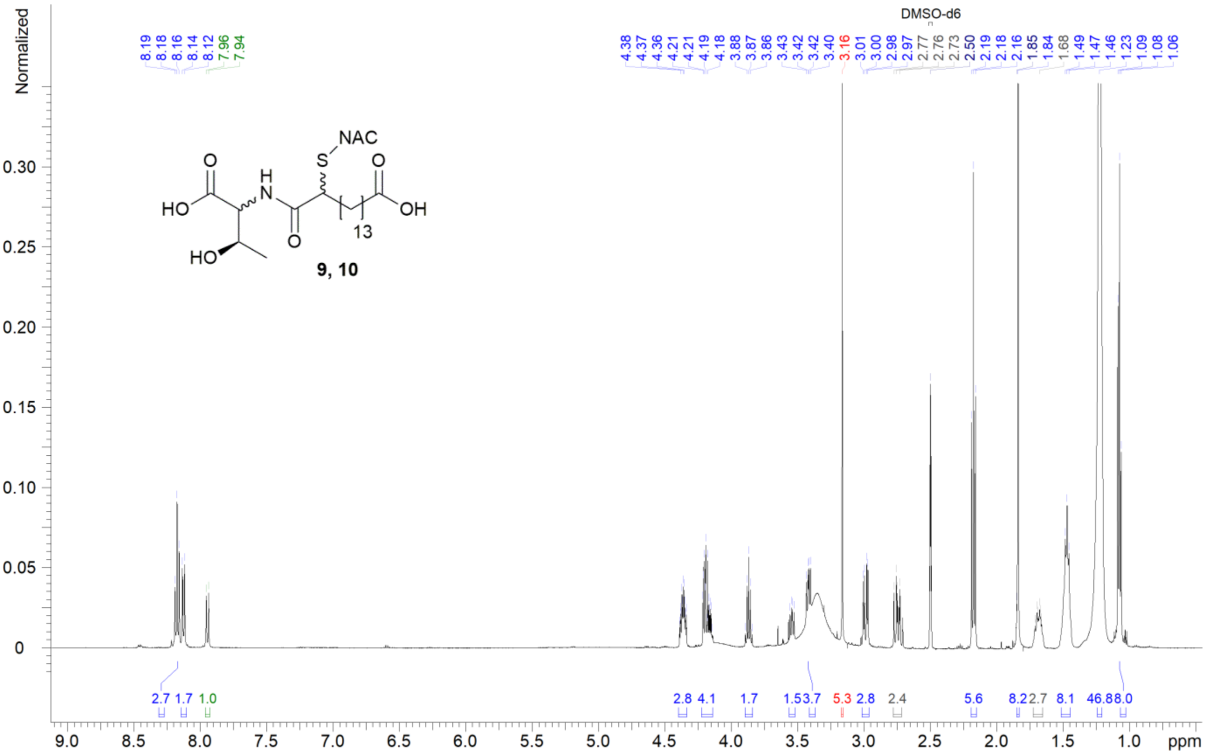

Figure S57: ^{1}H NMR spectrum (500 MHz, DMSO-d_6) of 2-NAC-D-/L-lipothrenin B_1 mixture (**9, 10**).

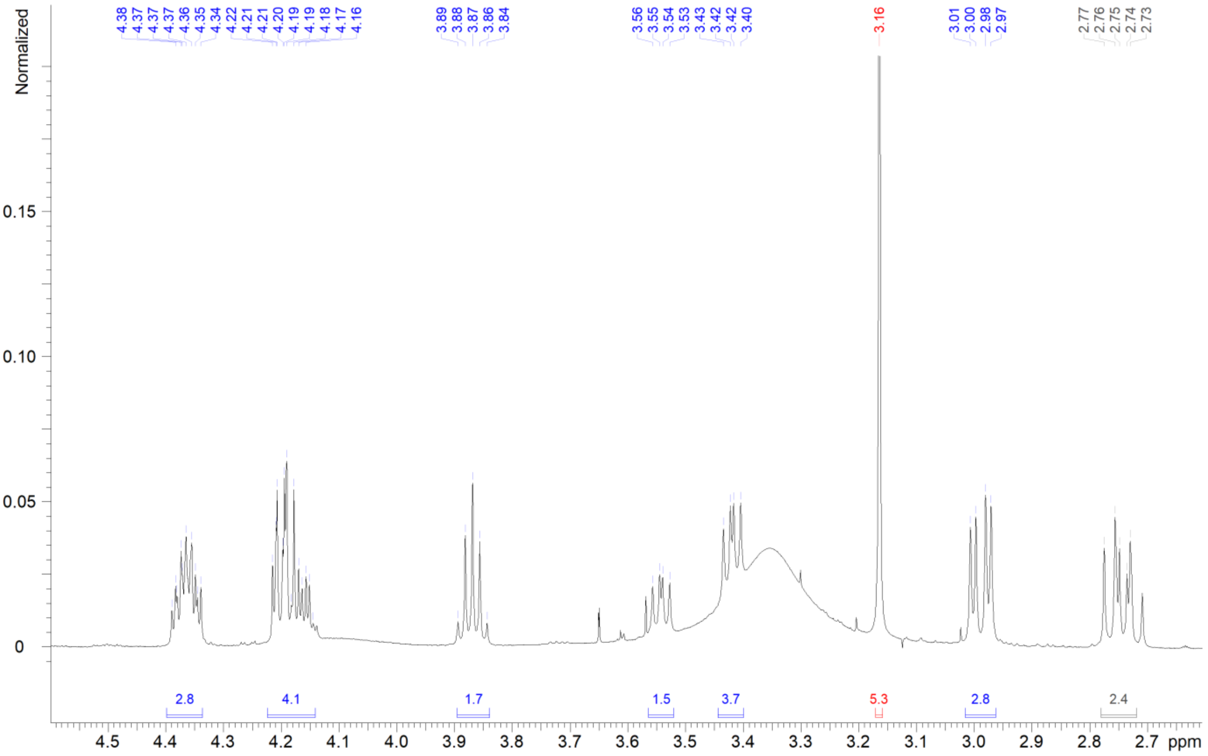

Figure S58. Zoomed in ^{1}H NMR spectrum (500 MHz, DMSO-d_6) of 2-NAC-D-/L-lipothrenin B_1 mixture (**9, 10**).

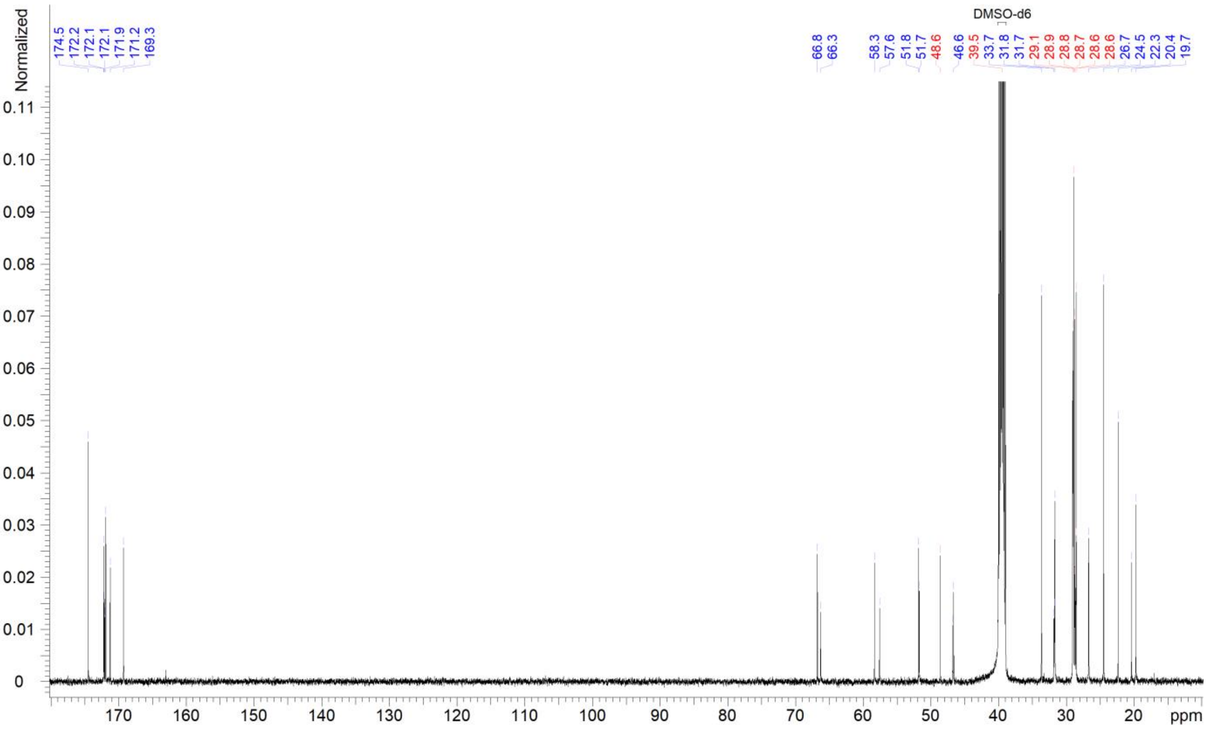

Figure S59: ^{13}C NMR spectrum (500 MHz, DMSO-d_6) of 2-NAC-D-/L-lipothrenin B_1 mixture (**9, 10**).

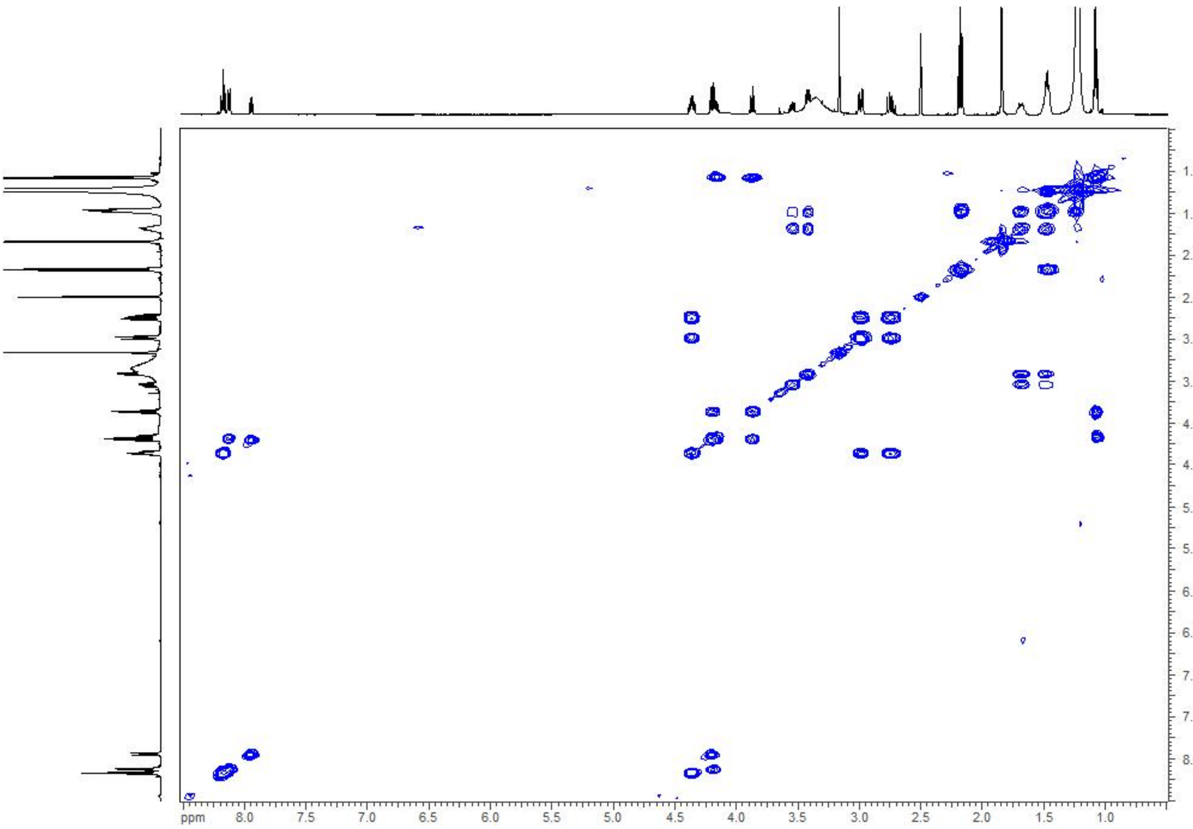

Figure S60: ^1H-^1H-COSY spectrum (500 MHz, DMSO-d_6) of 2-NAC-D-/L-lipothrenin B_1 mixture (**9, 10**).

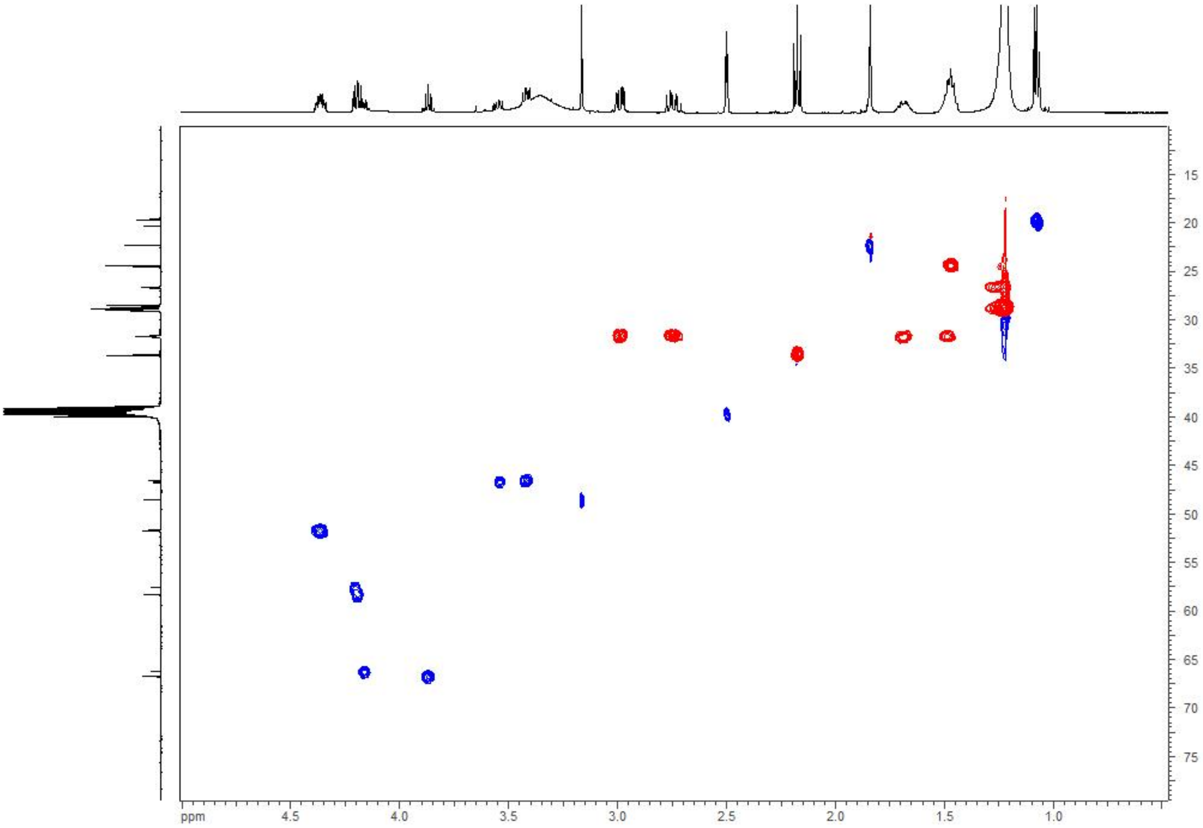

Figure S61: HSQC spectrum (500 MHz, DMSO-d$_6$) of 2-NAC-D-/L-lipothrenin B$_1$ mixture (**9, 10**).

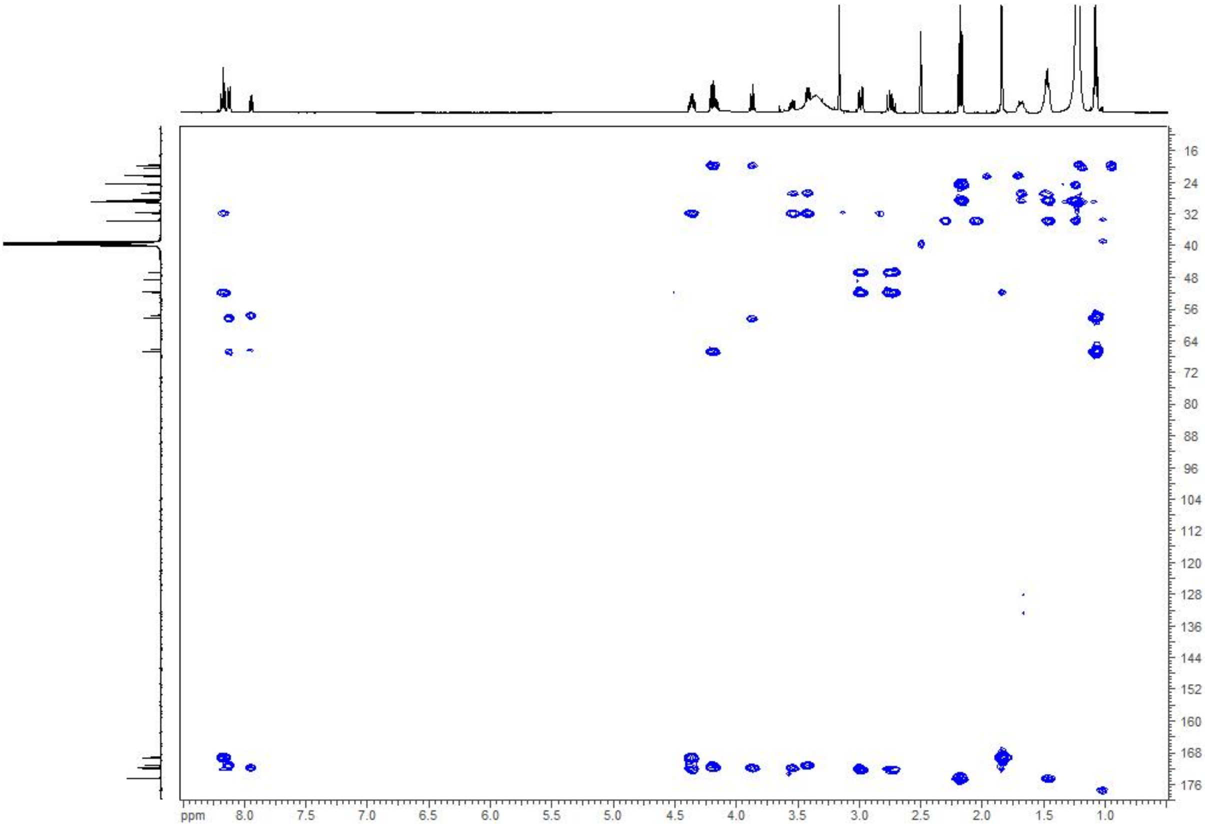

Figure S62: HMBC spectrum (500 MHz, DMSO-d$_6$) of 2-NAC-D-/L-lipothrenin B$_1$ mixture (**9, 10**).

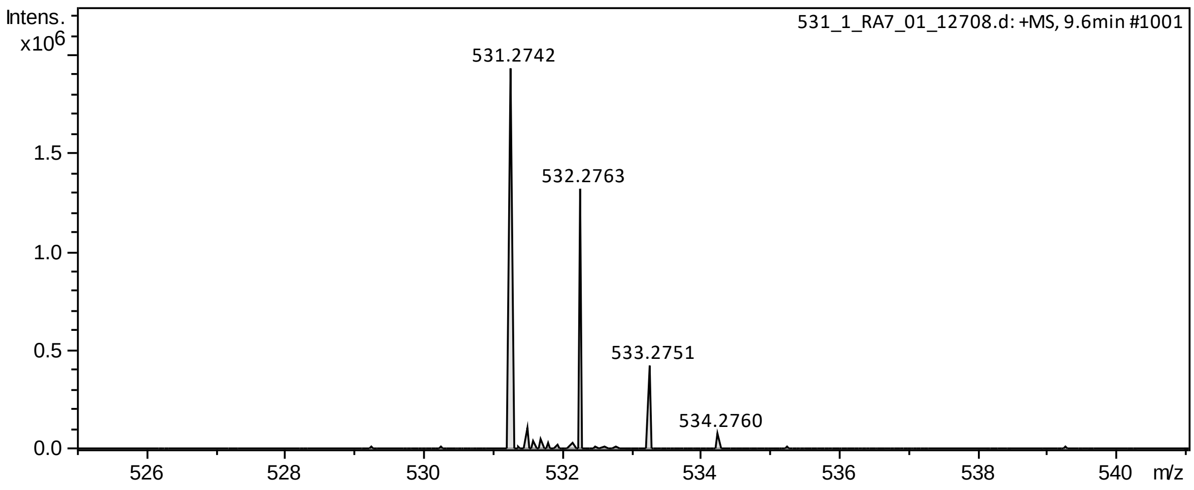

Figure S63. The HRESIMS of compound **11/12**.

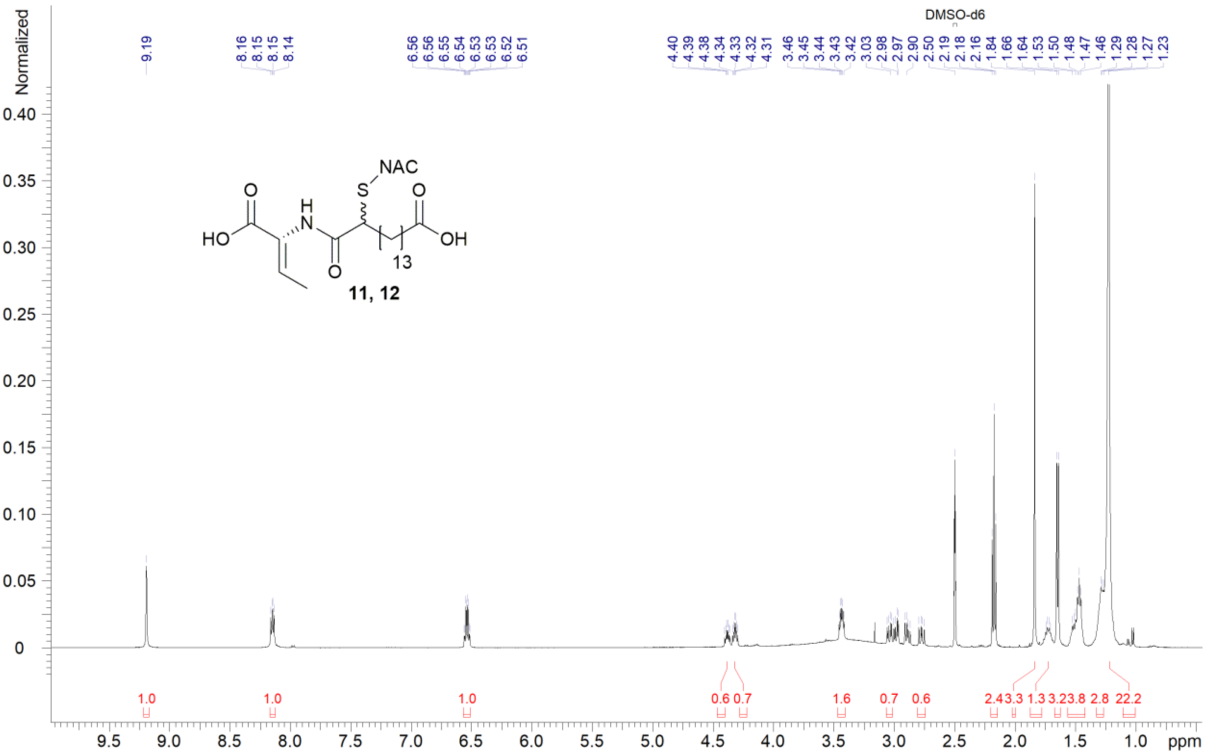

Figure S64: ^{1}H NMR spectrum (500 MHz, DMSO-d_6) of 2-NAC-*Z*-lipothrenin C and C_1 mixture (**11, 12**).

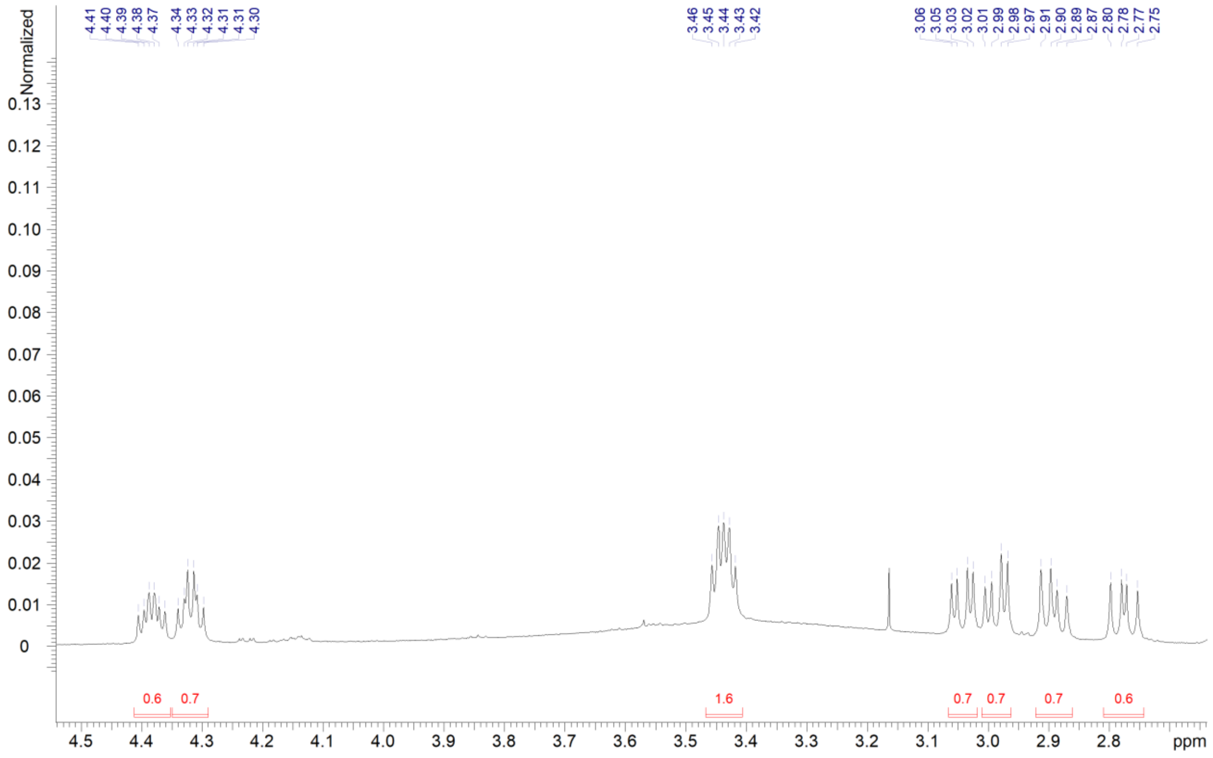

Figure S65. Zoomed in ^{1}H NMR spectrum (500 MHz, DMSO-d$_6$) of 2-NAC-*Z*-lipothrenin C and C$_1$ mixture (**11, 12**).

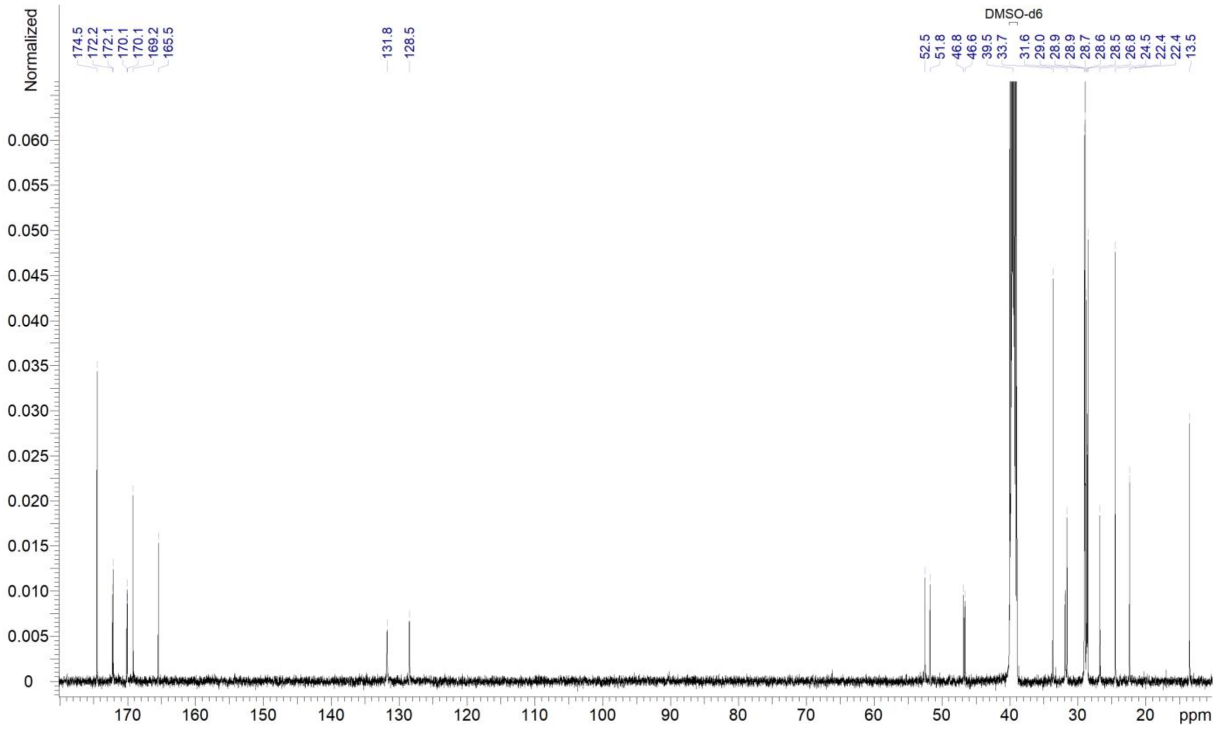

Figure S66: ^{13}C NMR spectrum (500 MHz, DMSO-d$_6$) of 2-NAC-*Z*-lipothrenin C and C$_1$ mixture (**11, 12**).

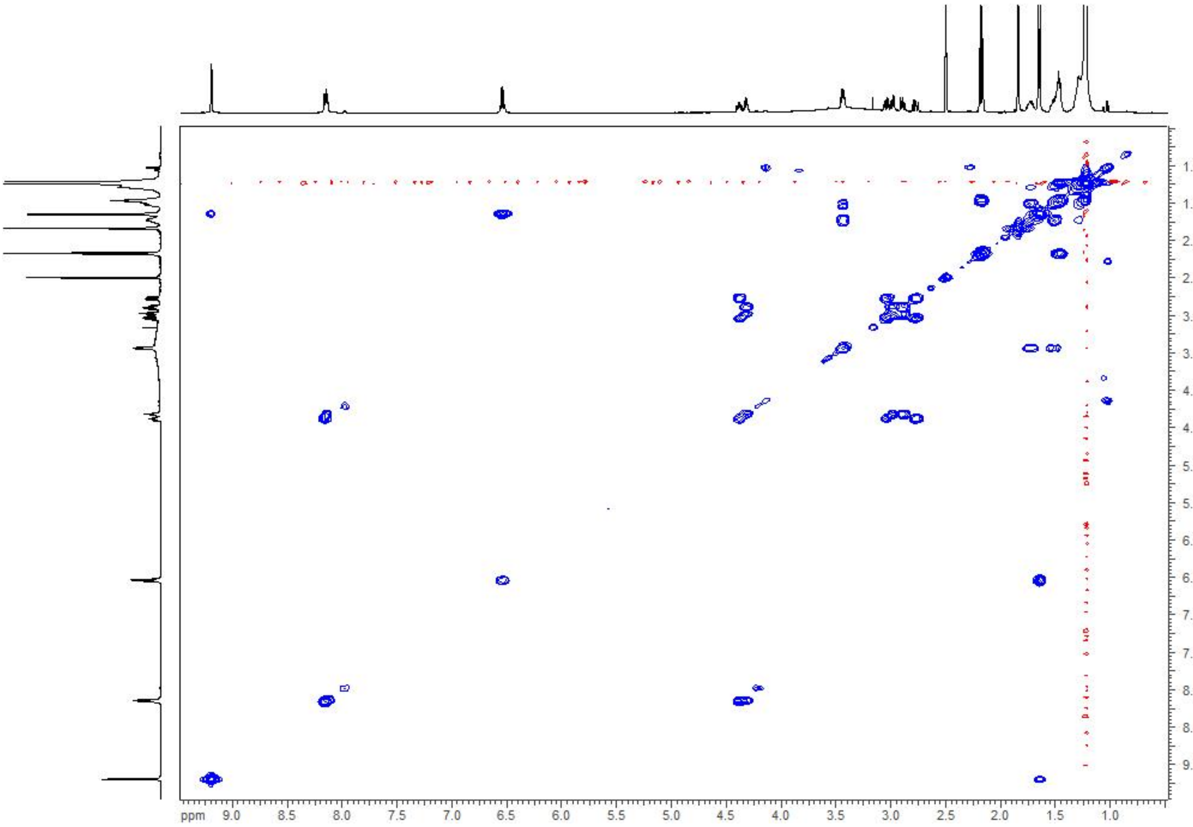

Figure S67: ^{1}H-^{1}H-COSY spectrum (500 MHz, DMSO-d$_6$) of 2-NAC-*Z*-lipothrenin C and C$_1$ mixture (**11, 12**).

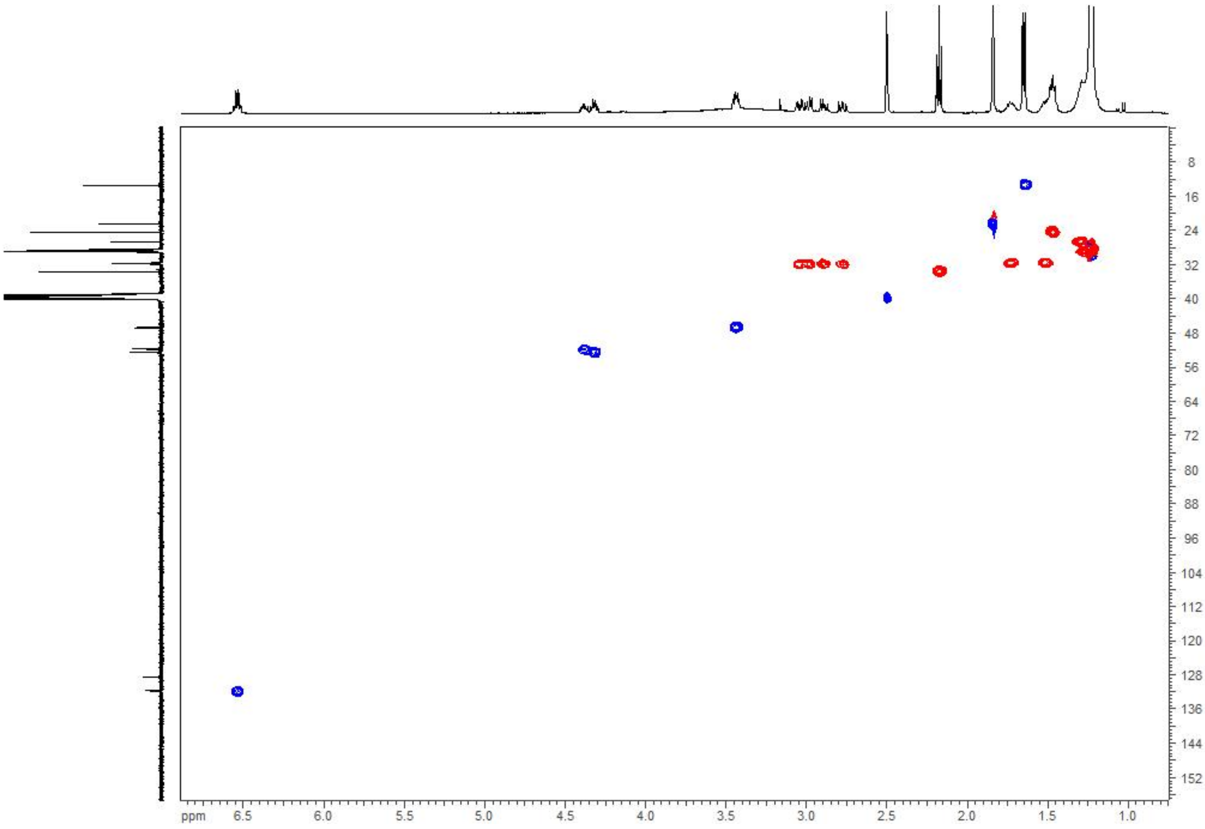

Figure S68: HSQC spectrum (500 MHz, DMSO-d$_6$) of 2-NAC-*Z*-lipothrenin C and C$_1$ mixture (**11, 12**).

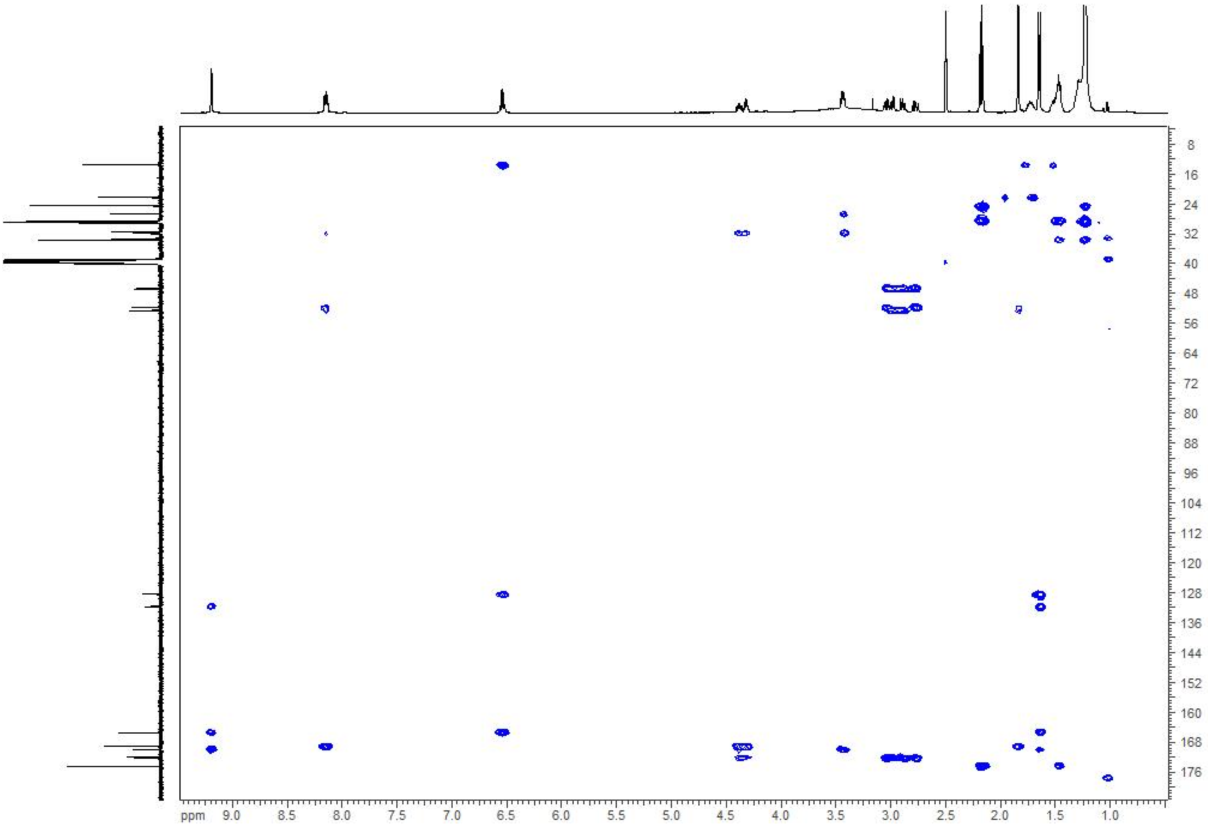

Figure S69: HMBC spectrum (500 MHz, DMSO-d$_6$) of 2-NAC-*Z*-lipothrenin C and C$_1$ mixture (**11, 12**).

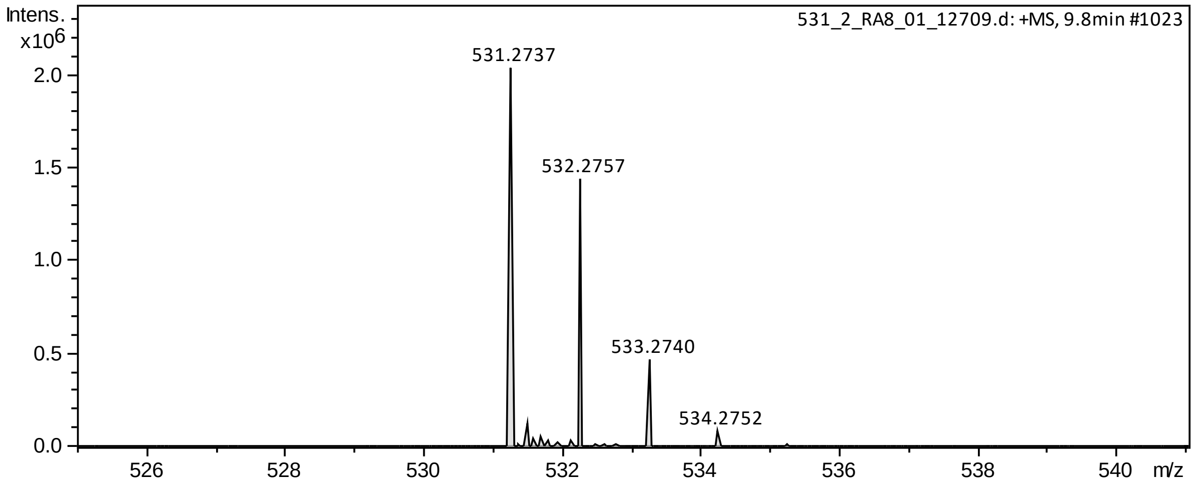

Figure S70. The HRESIMS of compound **13**/**14**.

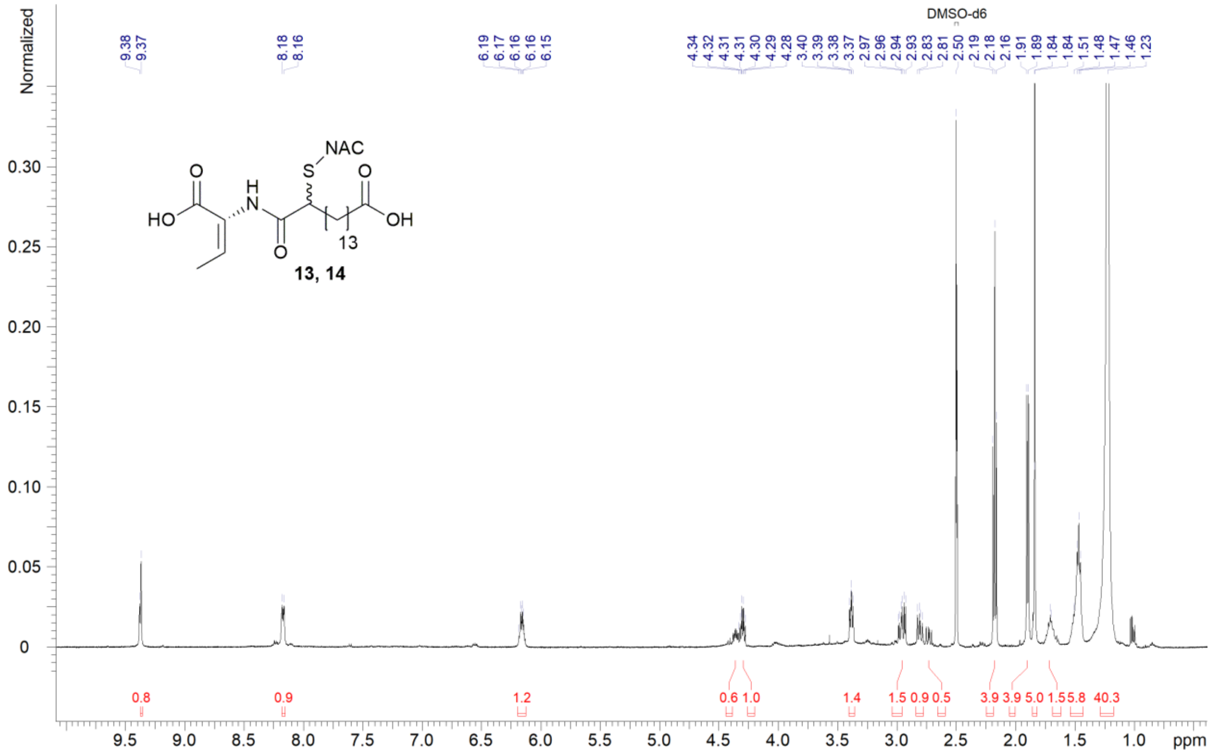

Figure S71: ^{1}H NMR spectrum (500 MHz, DMSO-d_6) of 2-NAC-*E*-lipothrenin C and C_1 mixture (**13, 14**).

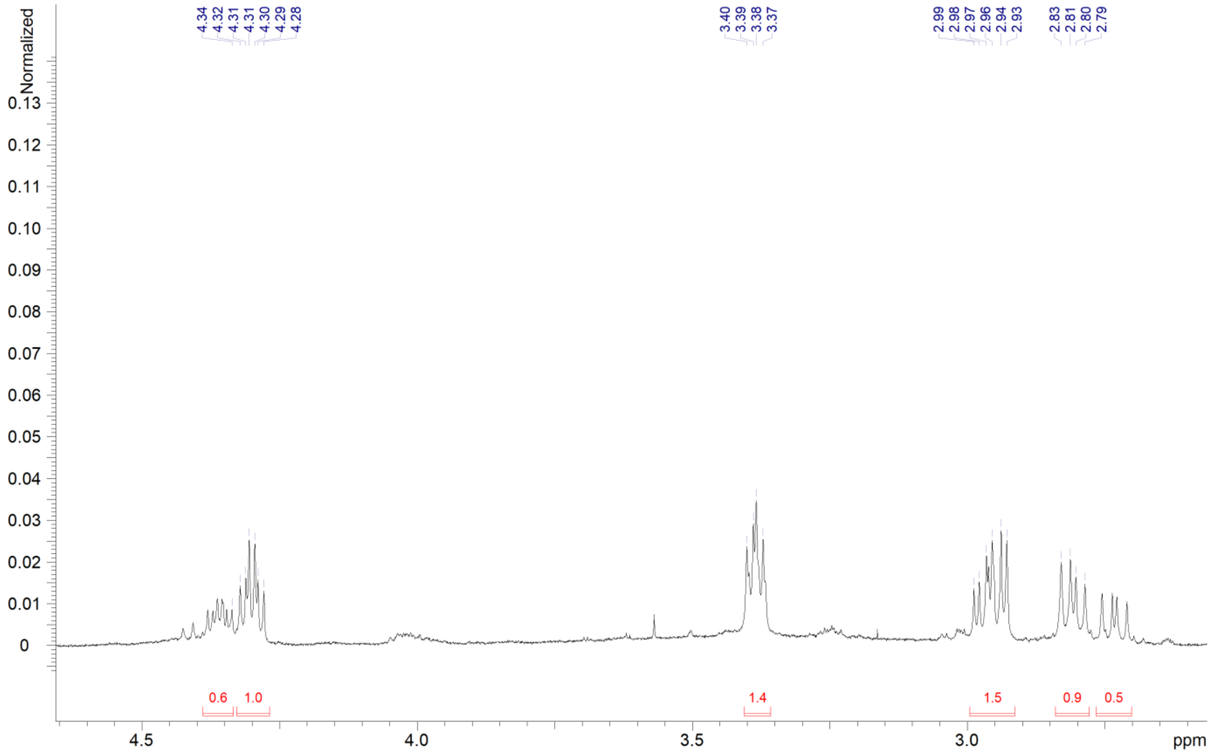

Figure S72. Zoomed in ^{1}H NMR spectrum (500 MHz, DMSO-d_6) of 2-NAC-*E*-lipothrenin C and C_1 mixture (**13, 14**).

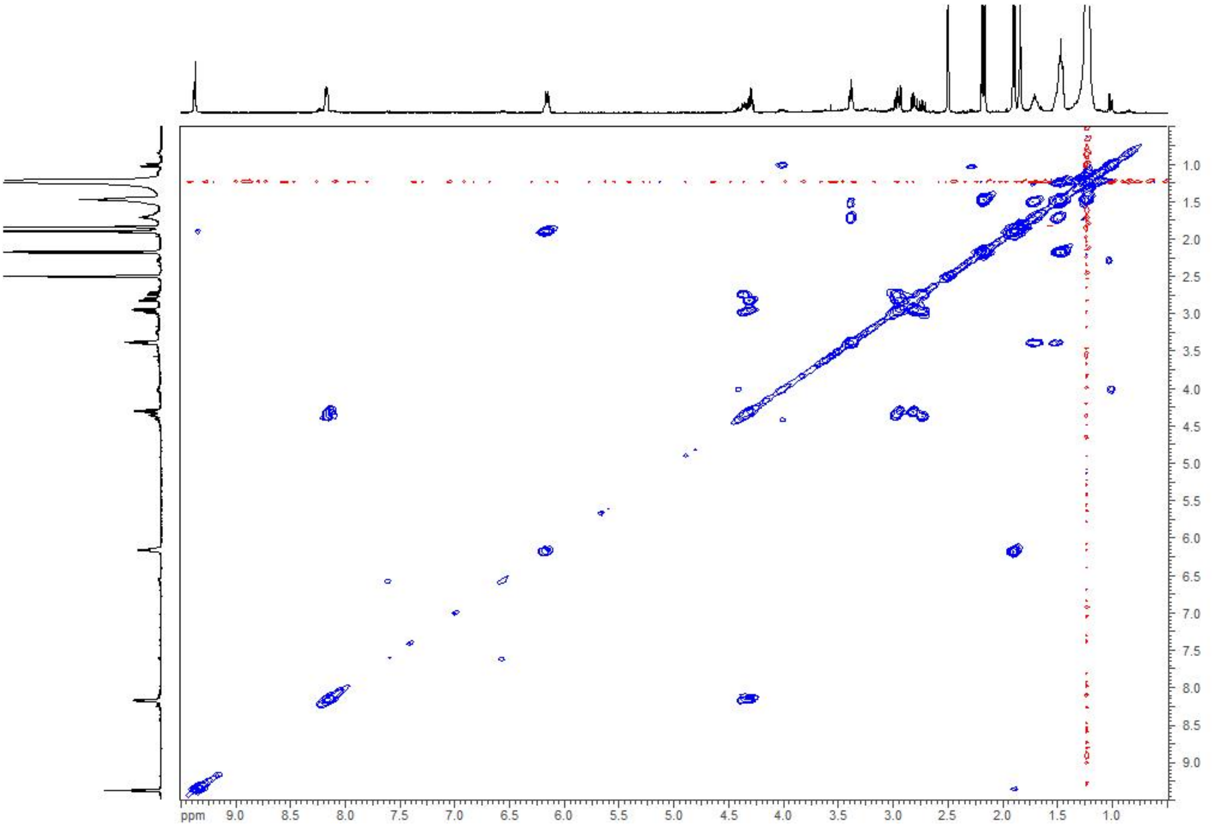

Figure S73: ^{1}H-^{1}H-COSY spectrum (500 MHz, DMSO-d_6) of 2-NAC-*E*-lipothrenin C and C_1 mixture (**13, 14**).

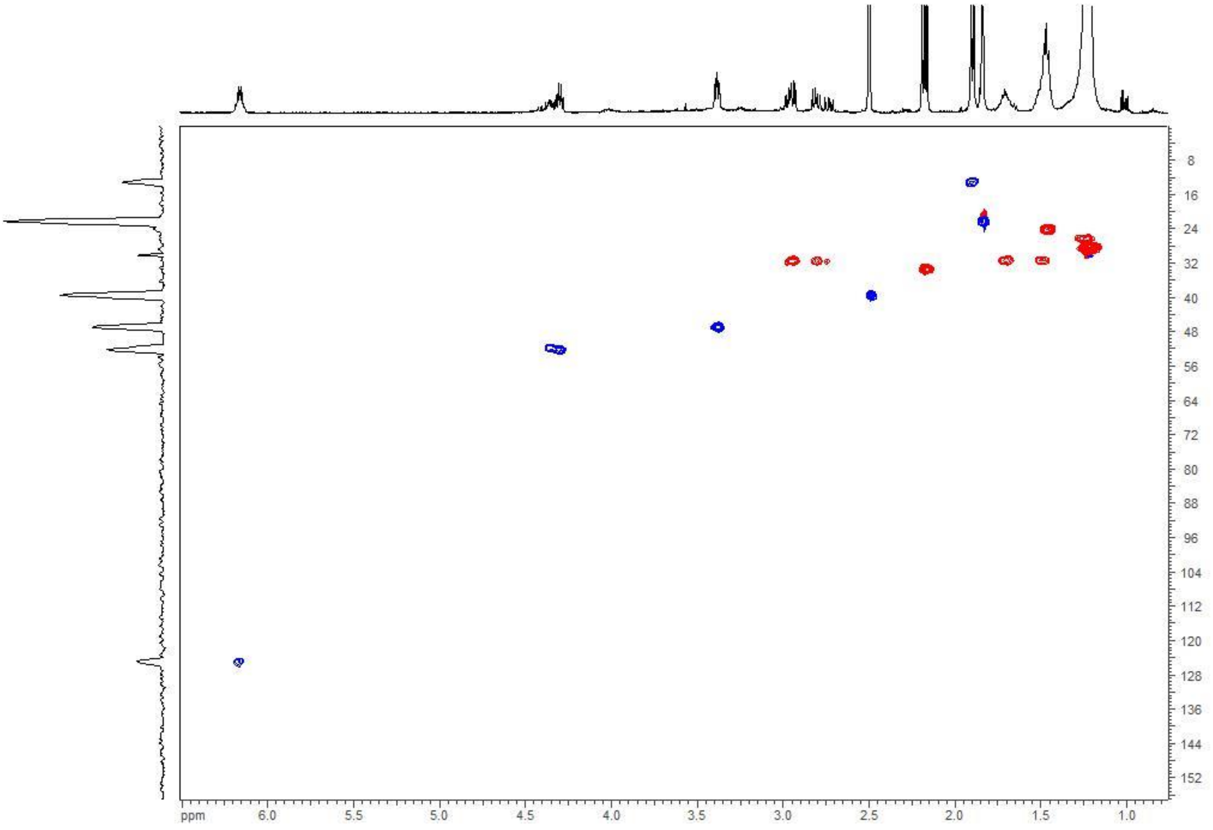

Figure S74: HSQC spectrum (500 MHz, DMSO-d_6) of 2-NAC-*E*-lipothrenin C and C_1 mixture (**13, 14**).

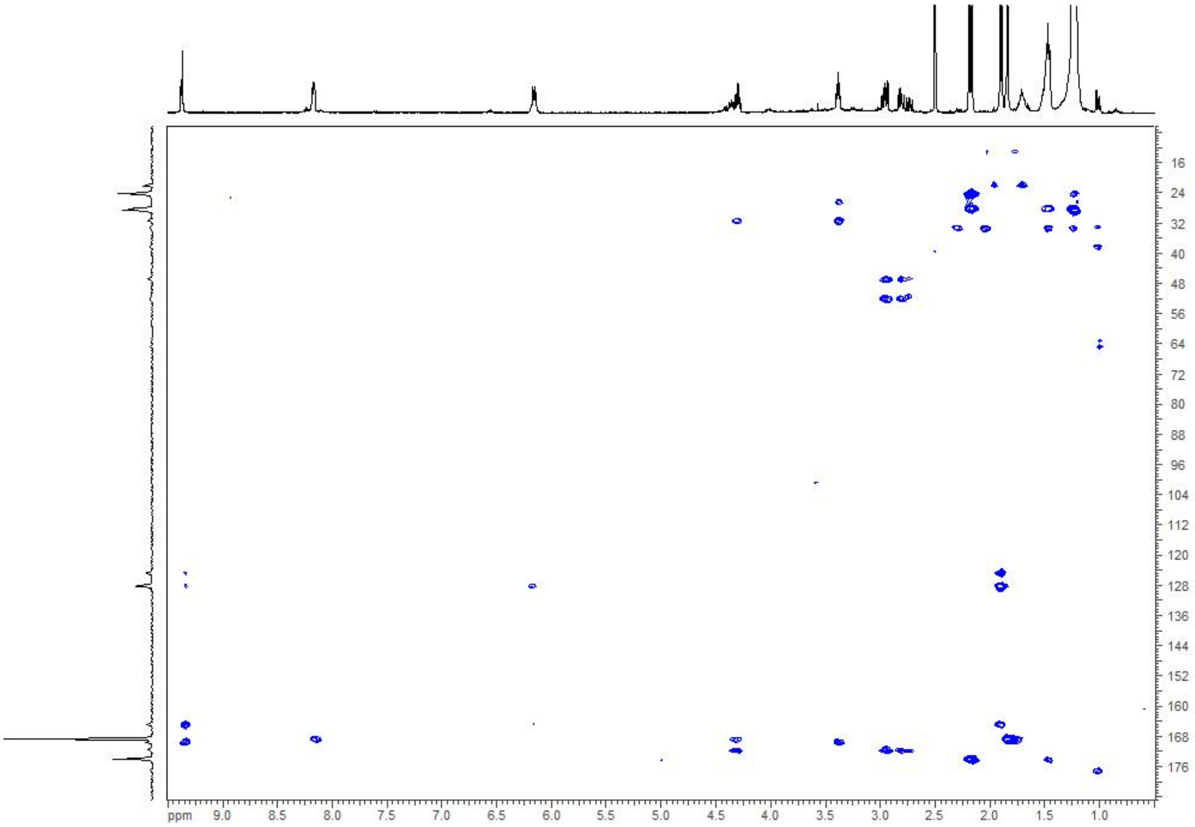

Figure S75: HMBC spectrum (500 MHz, DMSO-d_6) of 2-NAC-*E*-lipothrenin C and C_1 mixture (**13, 14**).

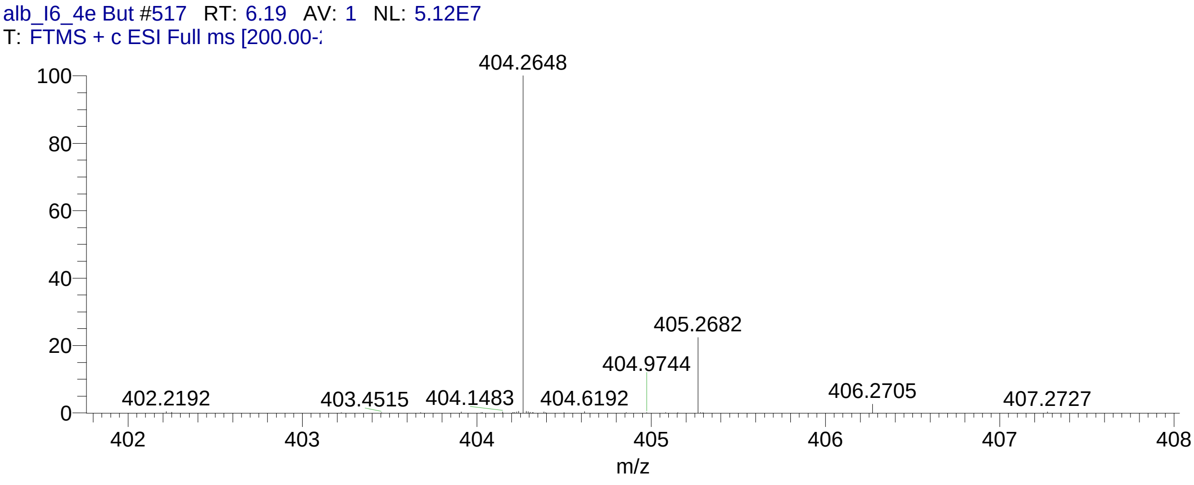

Figure S76. The HRESIMS of compound **15**.

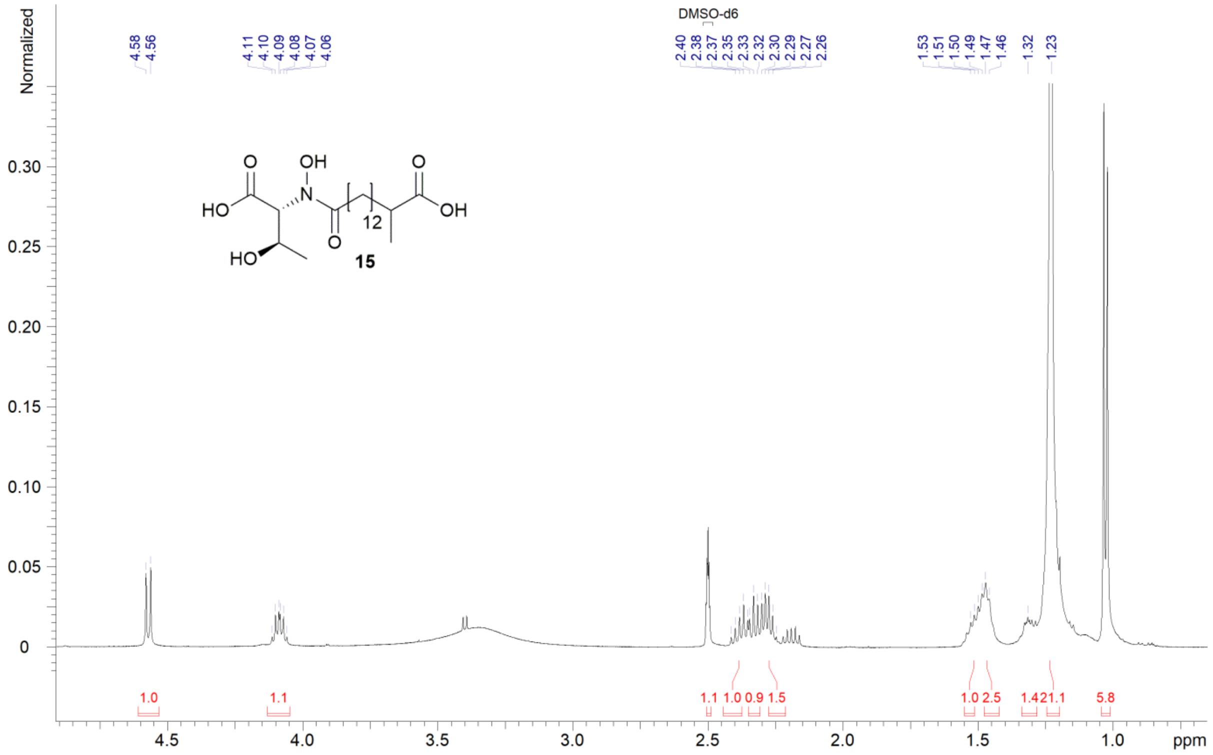

Figure S77: ^{1}H NMR spectrum (500 MHz, DMSO-d_6) of *iso*-lipothrenin A (**15**).

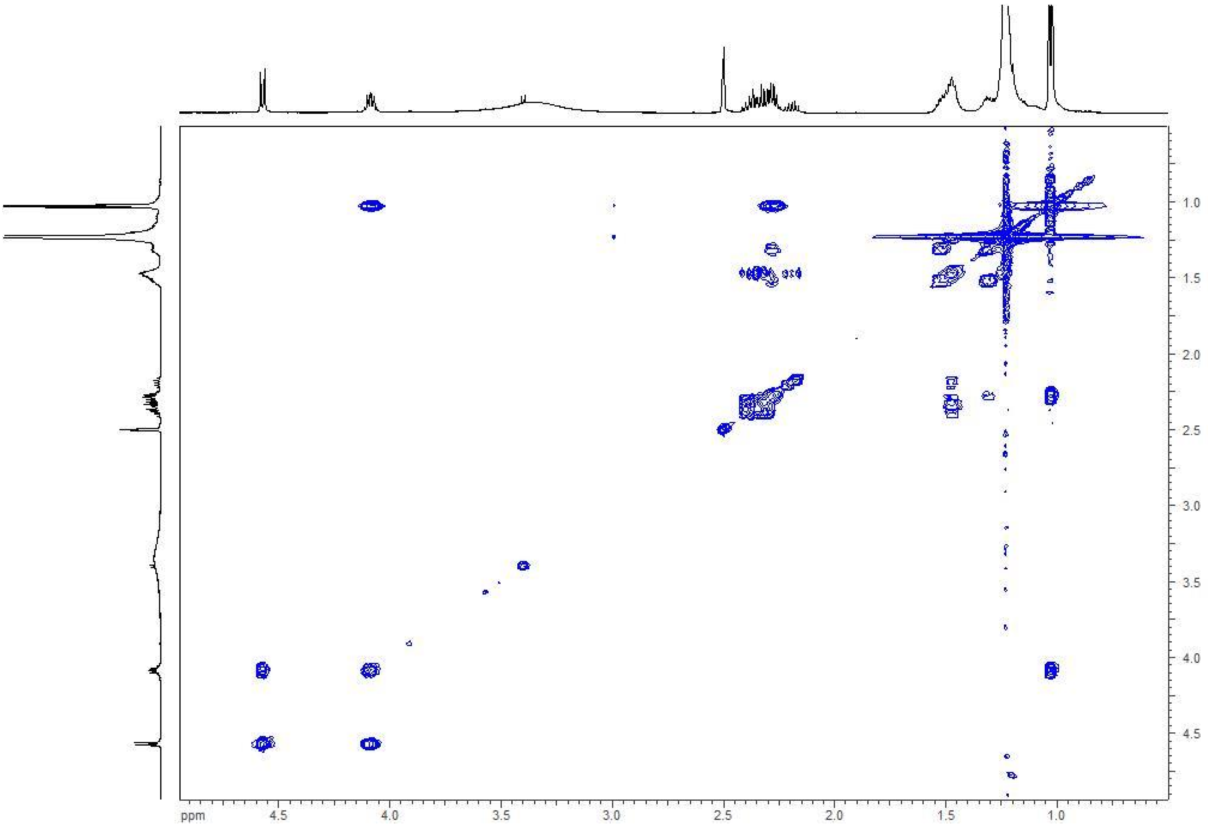

Figure S78: ^{1}H-^{1}H-COSY spectrum (500 MHz, DMSO-d_6) of *iso*-lipothrenin A (**15**).

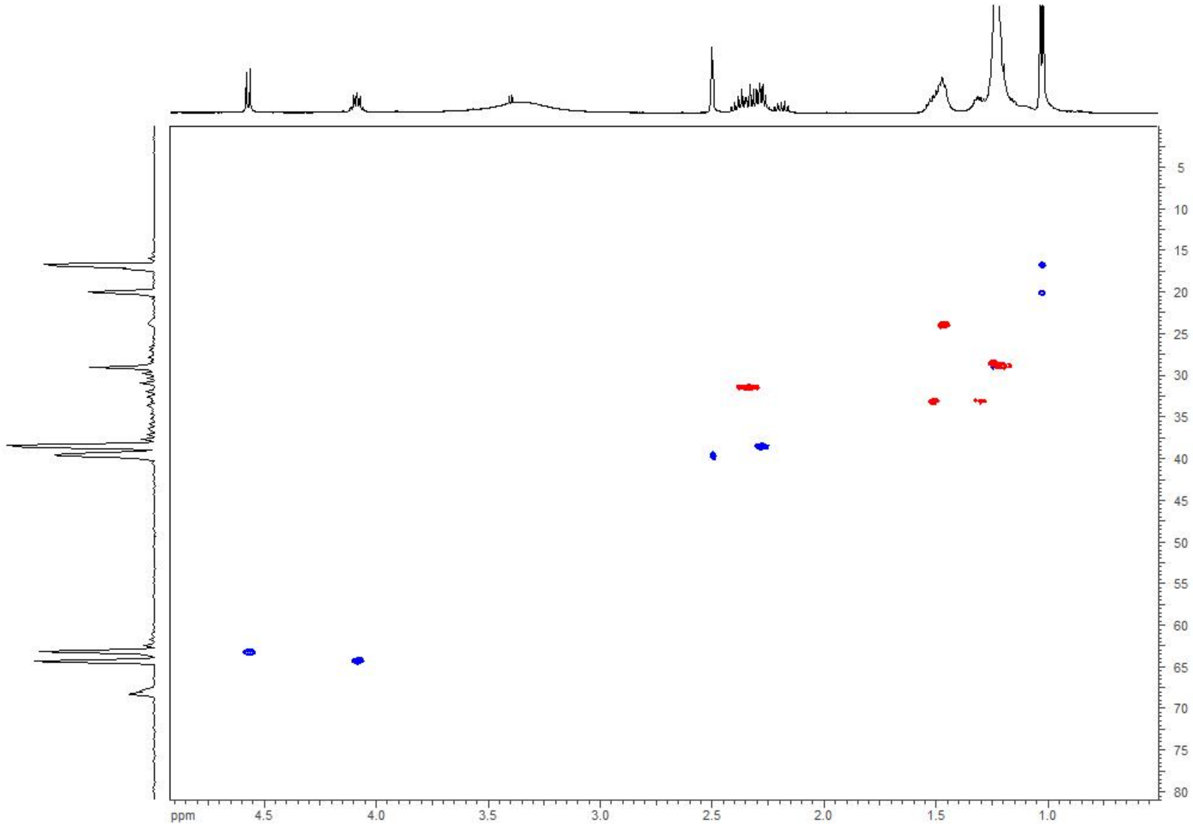

Figure S79: HSQC spectrum (500 MHz, DMSO-d$_6$) of *iso*-lipothrenin A (**15**).

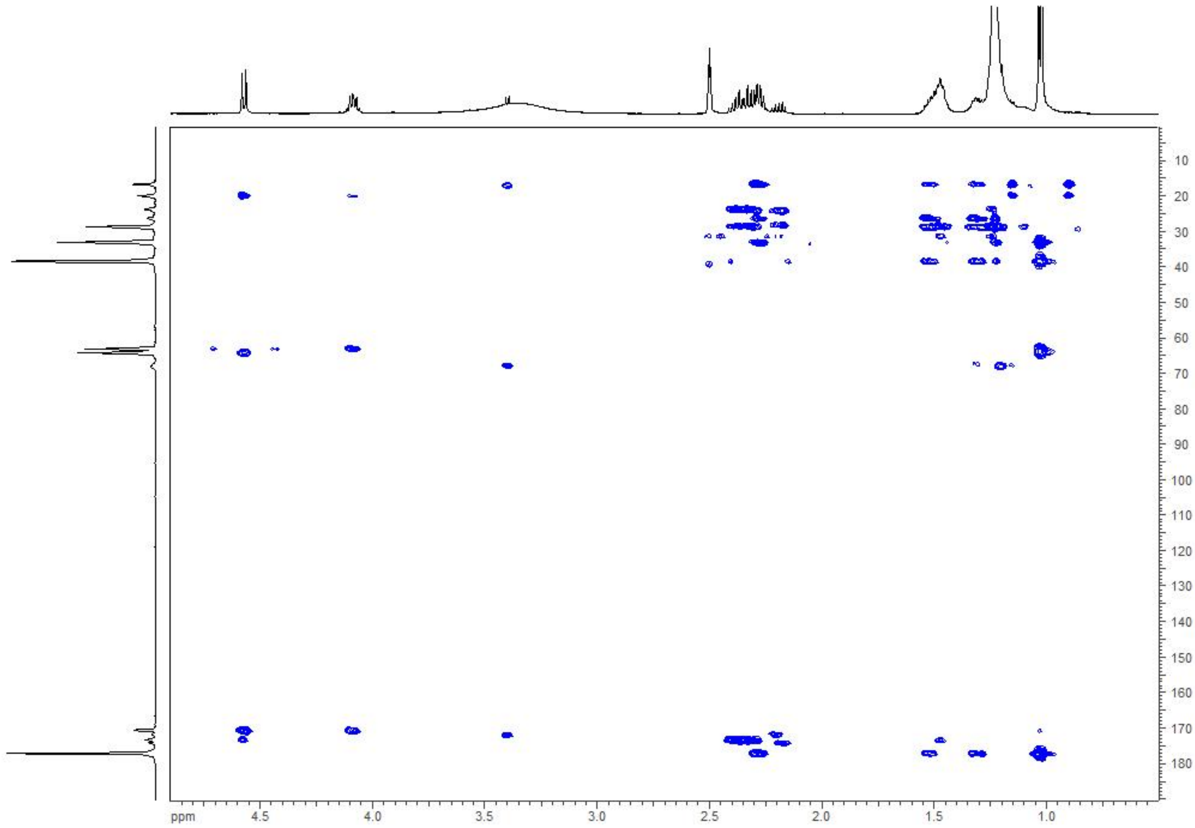

Figure S80: HMBC spectrum (500 MHz, DMSO-d$_6$) of *iso*-lipothrenin A (**15**).

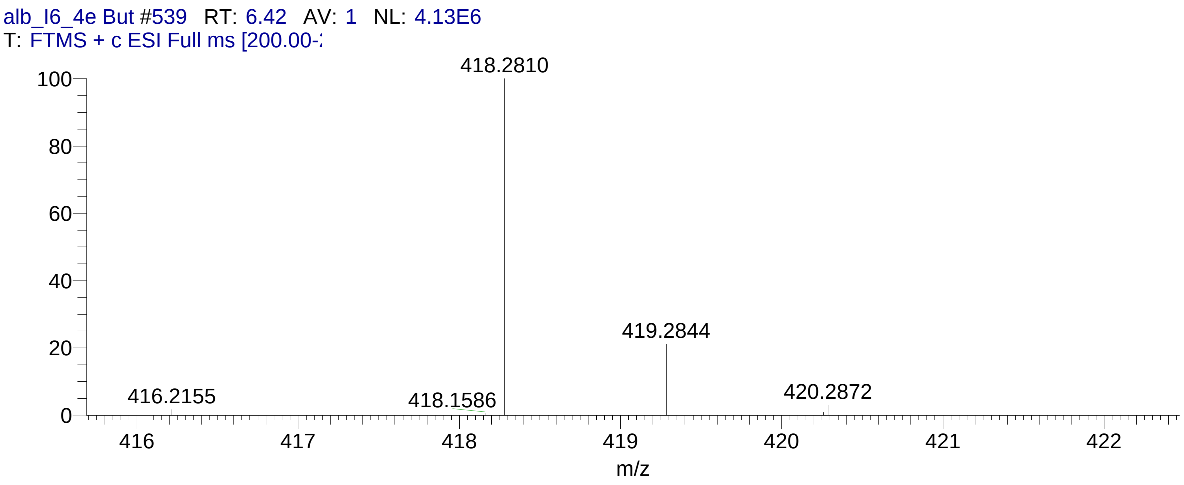

Figure S81. The HRESIMS of compound **16**.

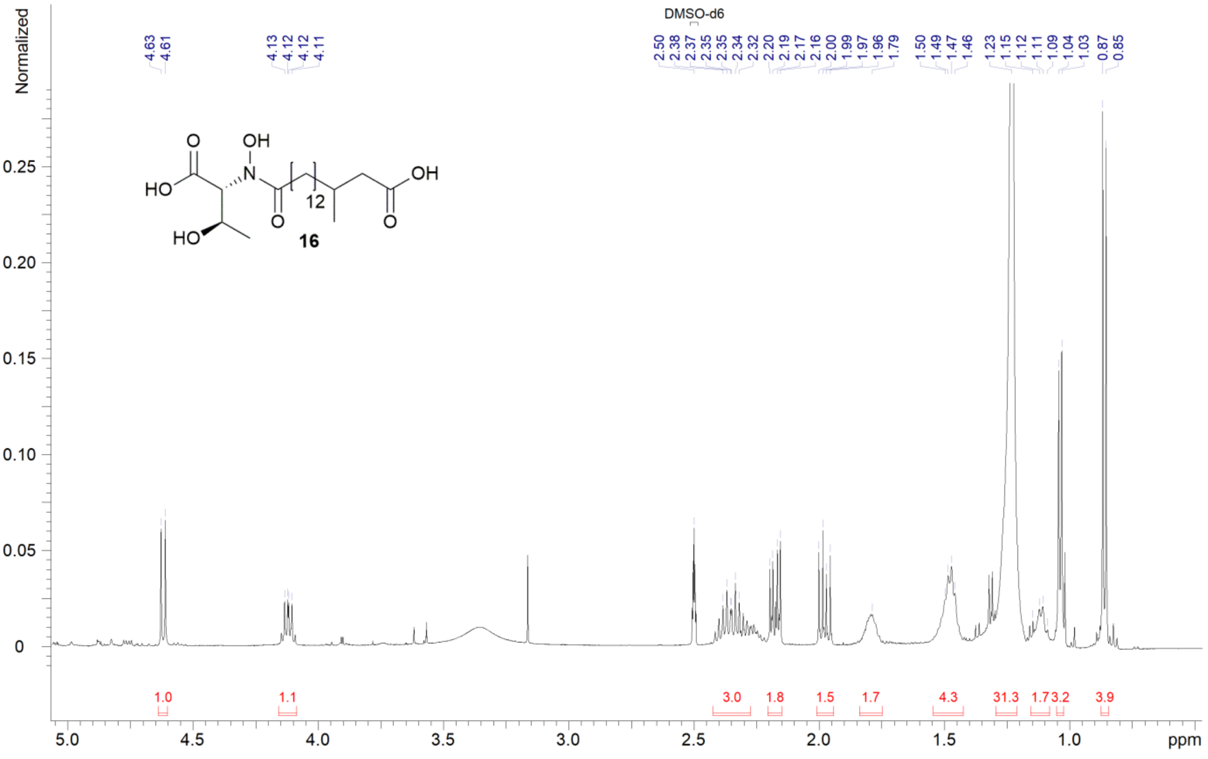

Figure S82: ^{1}H NMR spectrum (500 MHz, DMSO-d$_6$) of 14-methyl-lipothrenin A (**16**).

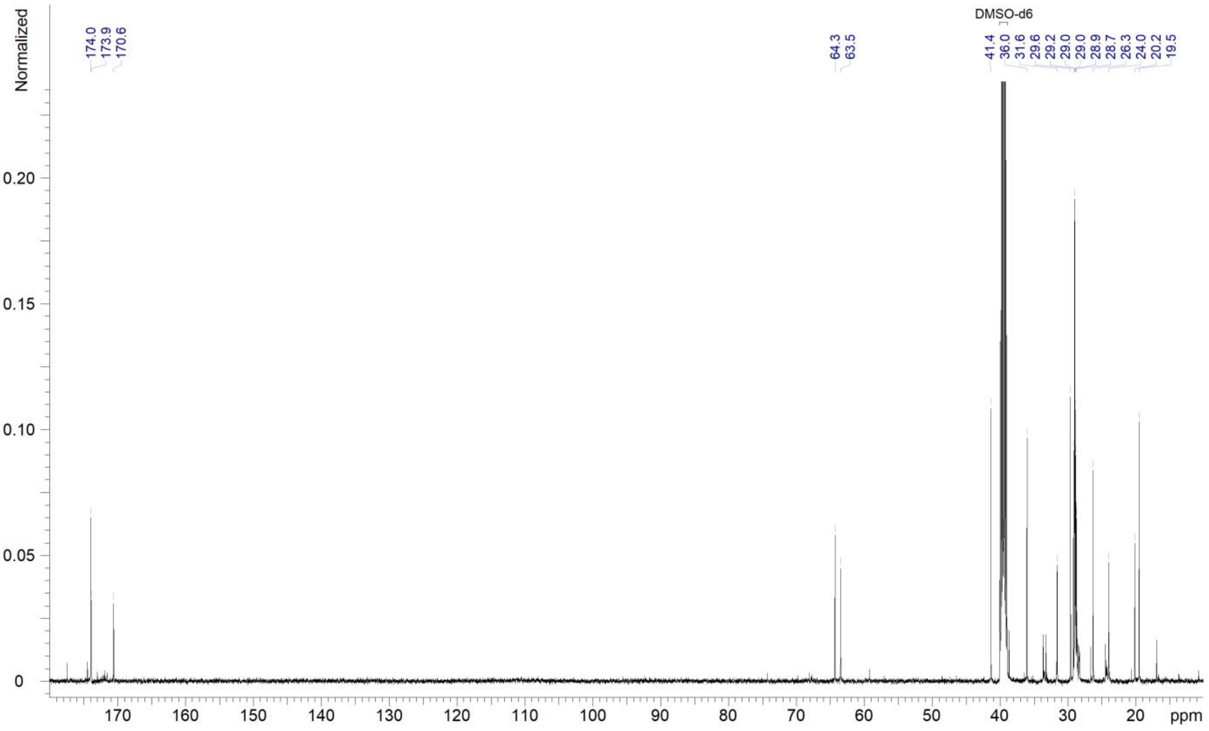

Figure S83: ^{13}C NMR spectrum (500 MHz, DMSO-d$_6$) of 14-methyl-lipothrenin A (**16**).

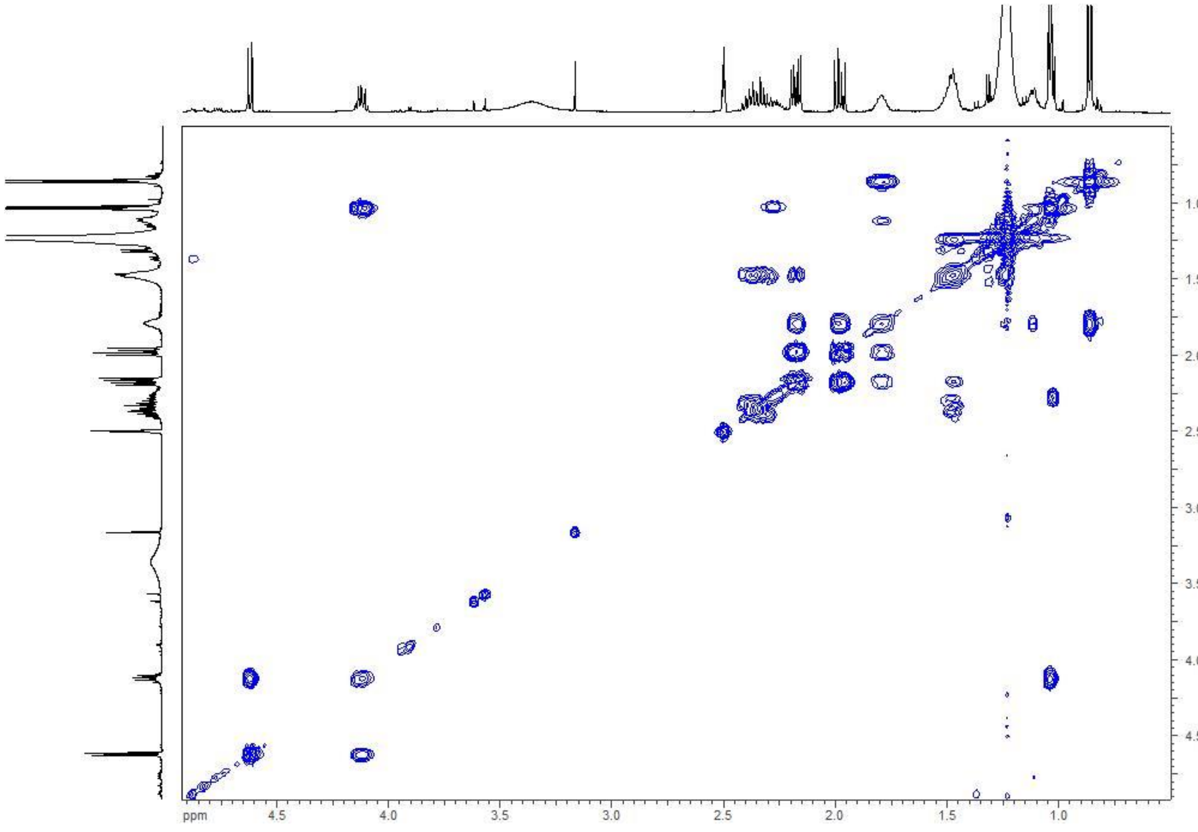

Figure S84: ^{1}H-^{1}H-COSY spectrum (500 MHz, DMSO-d$_6$) of 14-methyl-lipothrenin A (**16**).

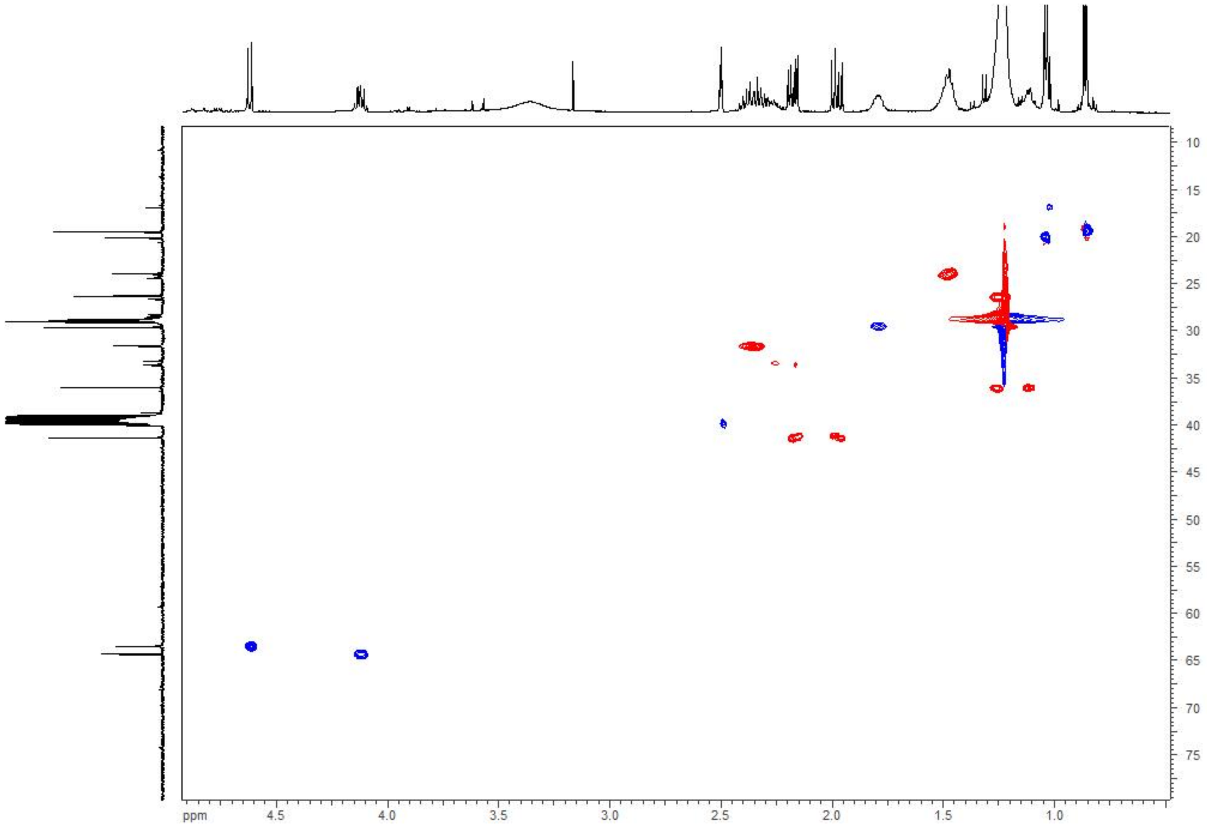

Figure S85: HSQC spectrum (500 MHz, DMSO-d_6) of 14-methyl-lipothrenin A (**16**).

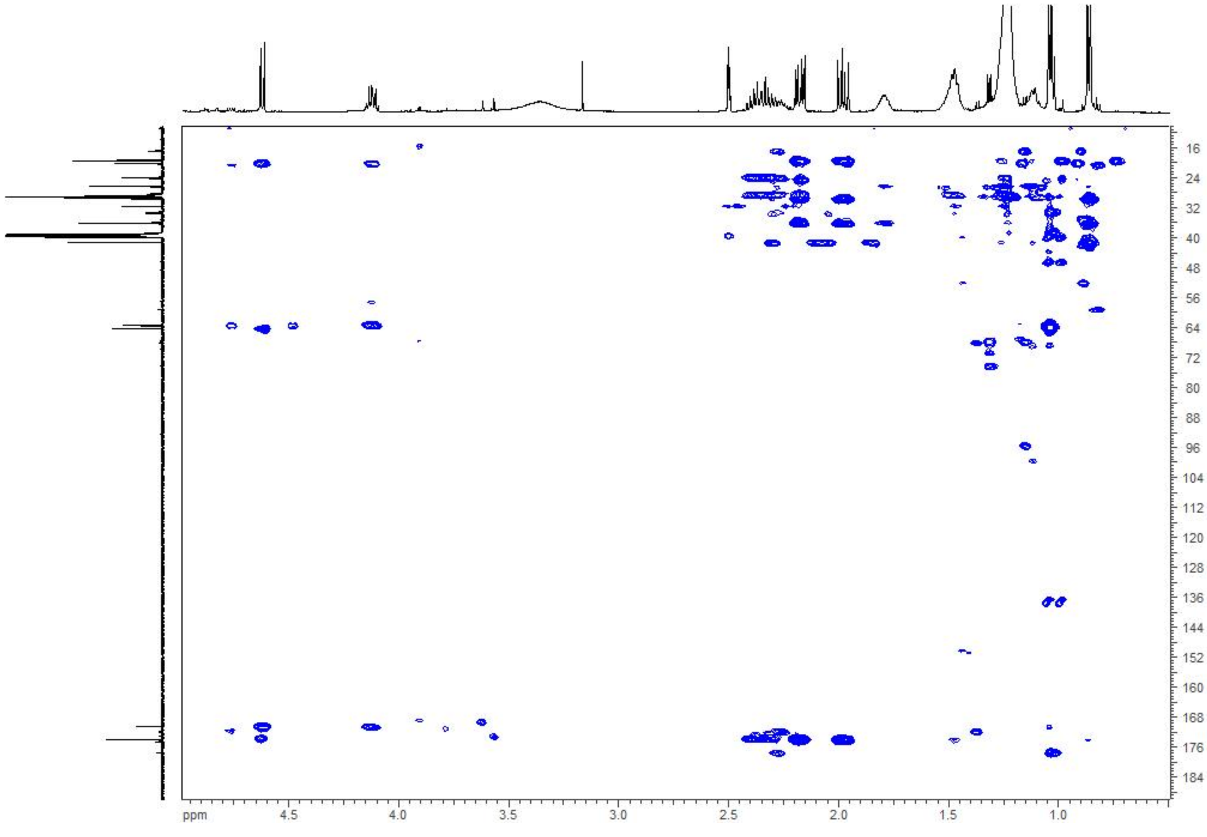

Figure S86: HMBC spectrum (500 MHz, DMSO-d_6) of 14-methyl-lipothrenin A (**16**).

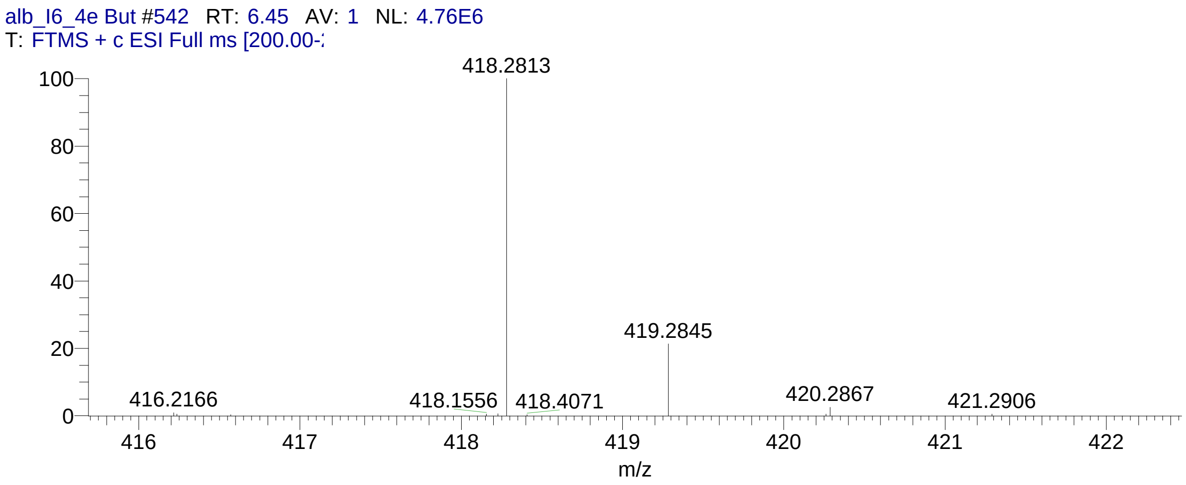

Figure S87. The HRESIMS of compound **17**.

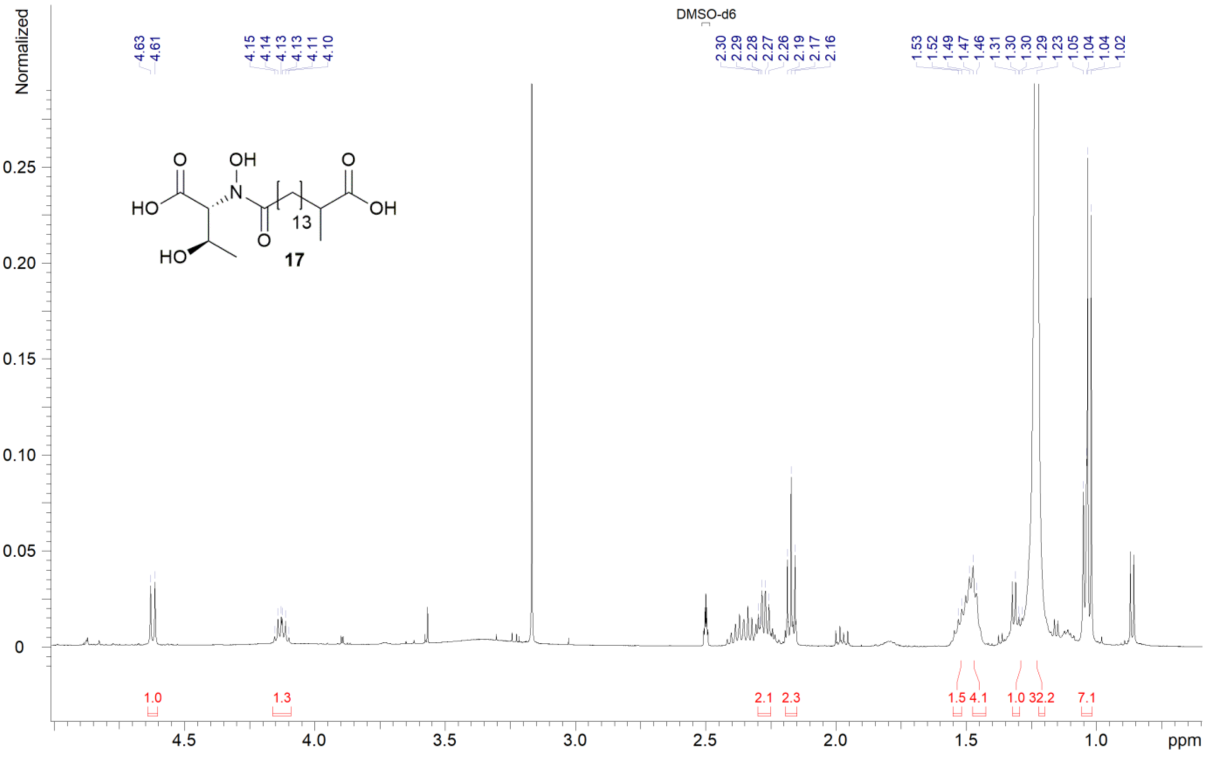

Figure S88: ^{1}H NMR spectrum (500 MHz, DMSO-d_6) of 15-methyl-lipothrenin A (**17**).

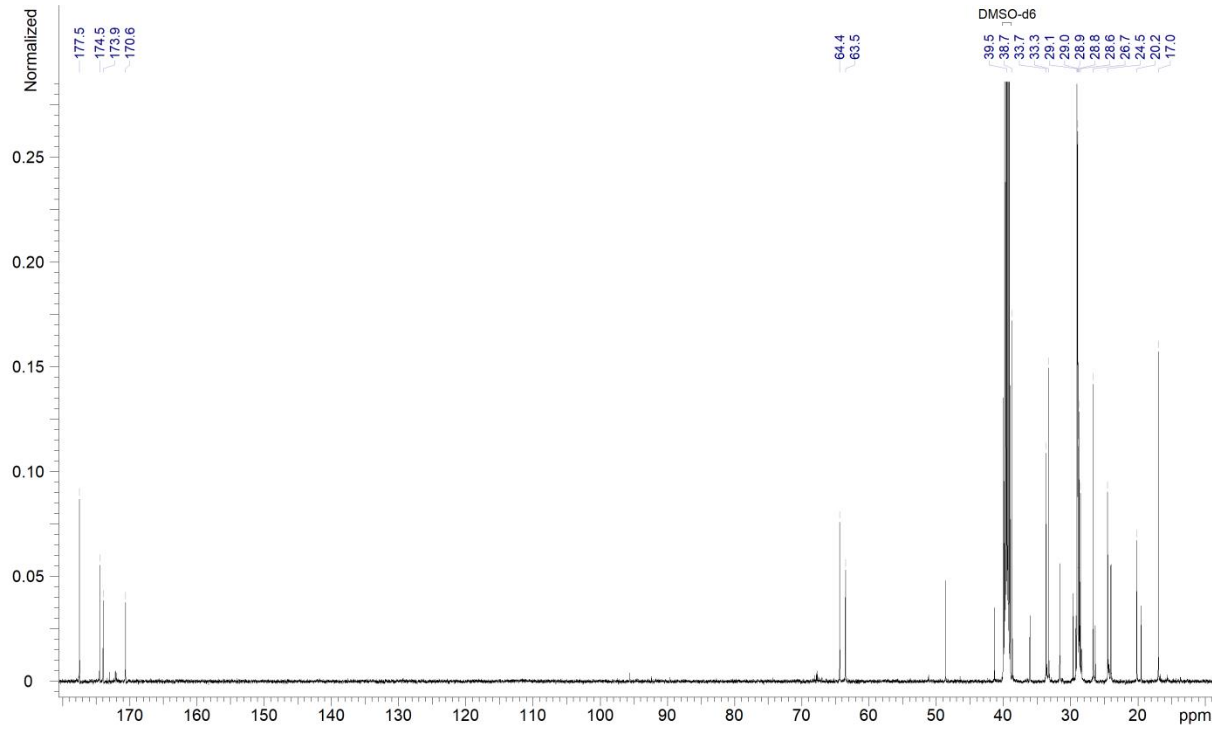

Figure S89: ^{13}C NMR spectrum (500 MHz, DMSO-d_6) of 15-methyl-lipothrenin A (**17**).

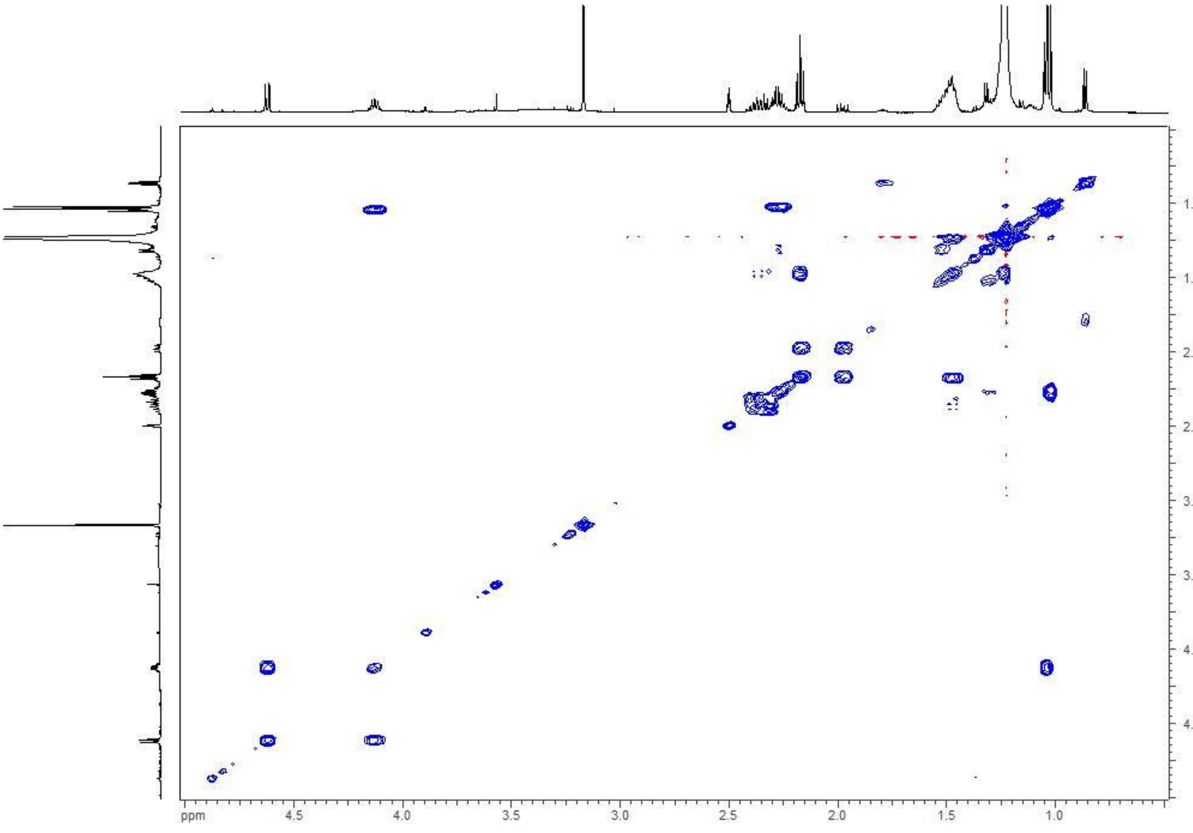

Figure S90: ^1H-^1H-COSY spectrum (500 MHz, DMSO-d_6) of 15-methyl-lipothrenin A (**17**).

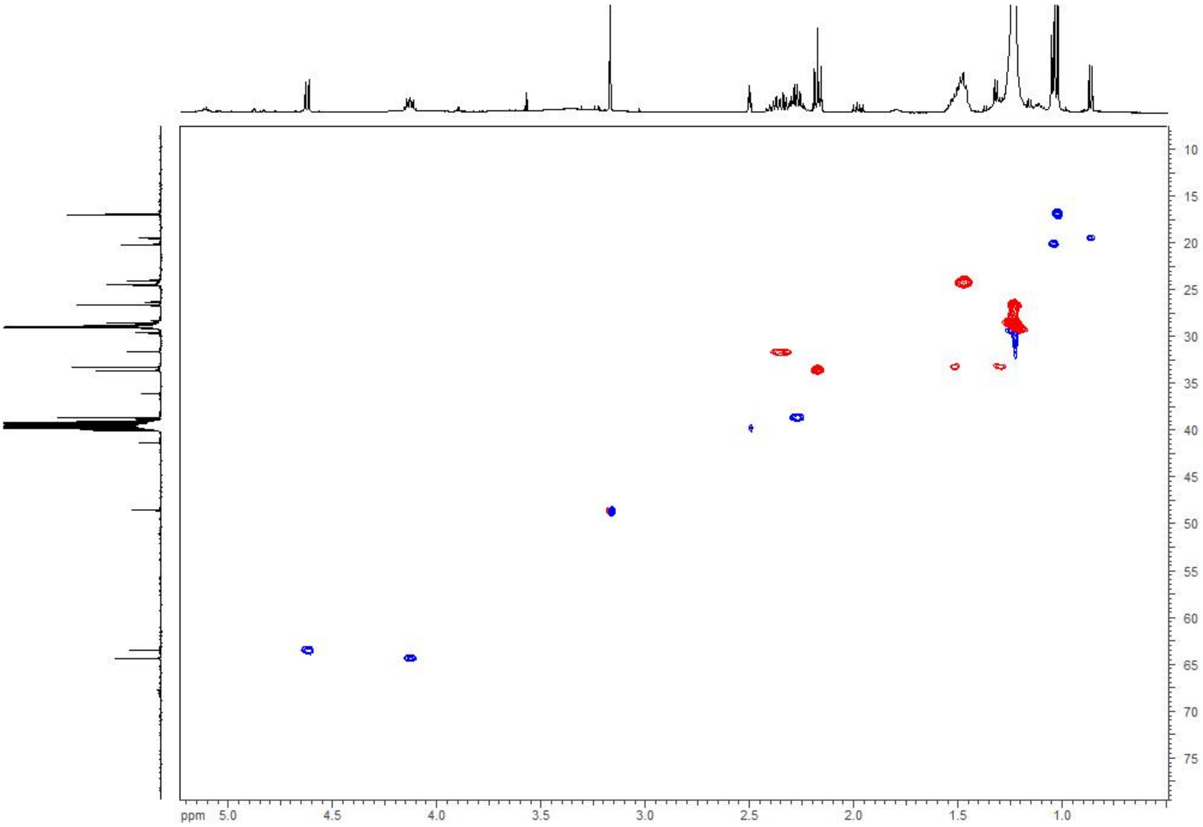

Figure S91: HSQC spectrum (500 MHz, DMSO-d_6) of 15-methyl-lipothrenin A (**17**).

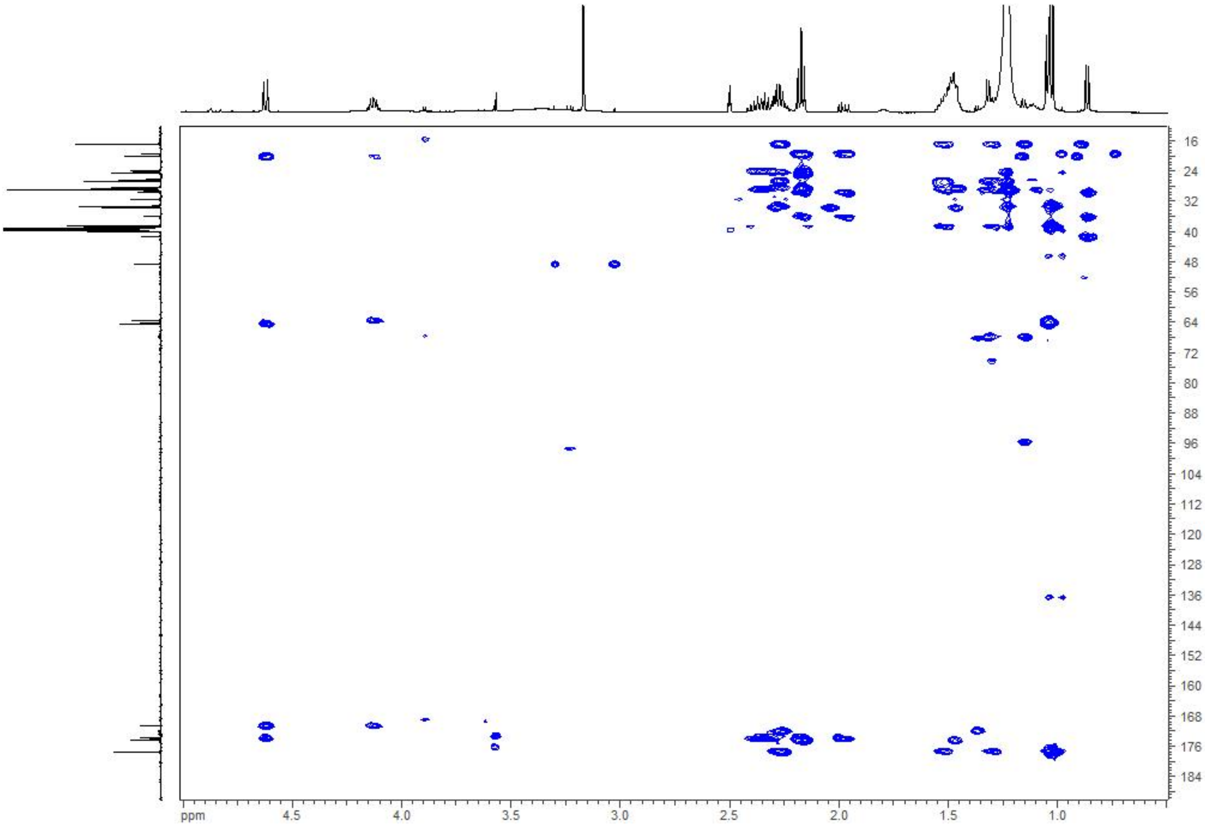

Figure S92: HMBC spectrum (500 MHz, DMSO-d_6) of 15-methyl-lipothrenin A (**17**).

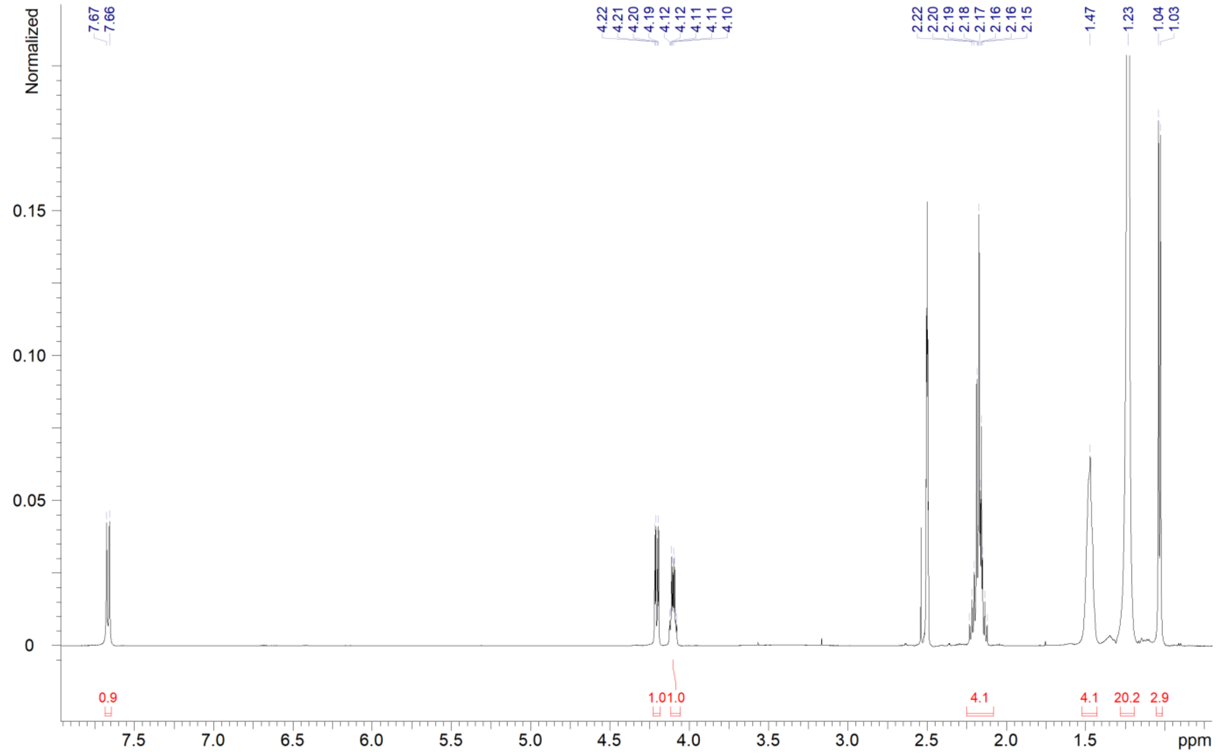

Figure S93. ^{1}H NMR spectrum (500 MHz, DMSO-d$_6$) of L-Lipothrenin B (**3**) isolated from deletion mutant I6_ΔlitM.

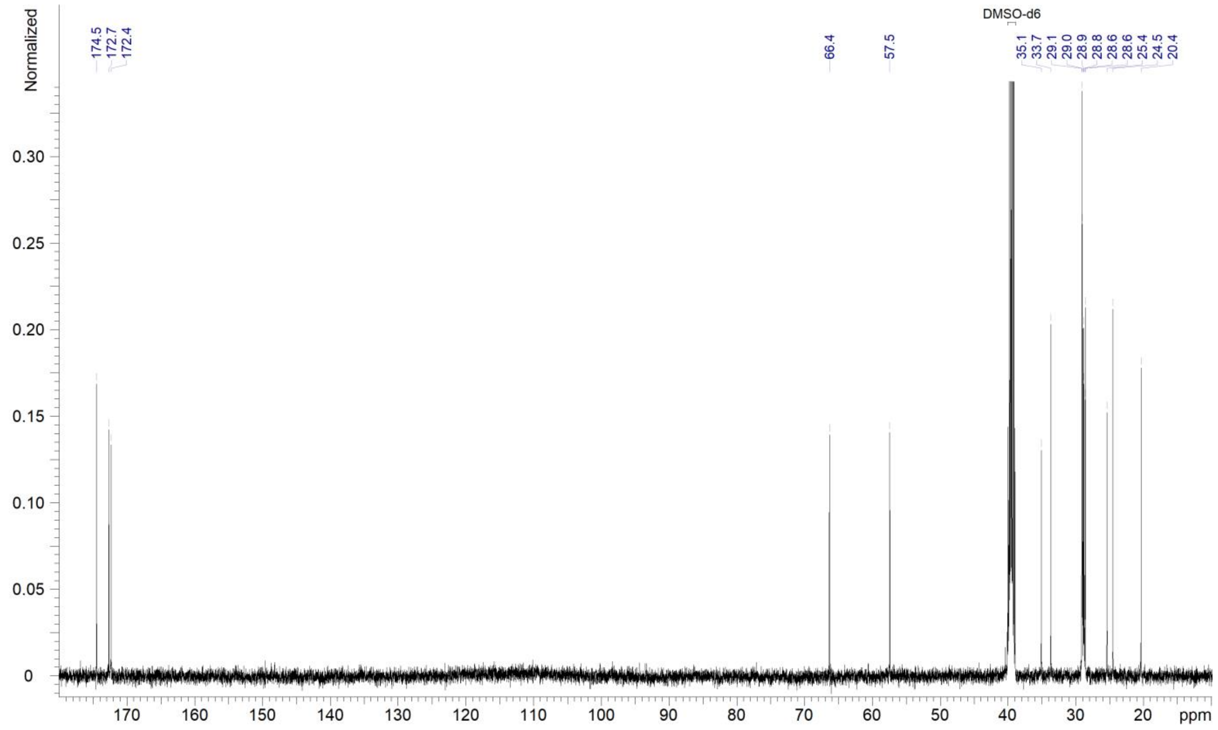

Figure S94. ^{13}C NMR spectrum (500 MHz, DMSO-d$_6$) of L-Lipothrenin B (**3**) isolated from deletion mutant I6_ΔlitM.

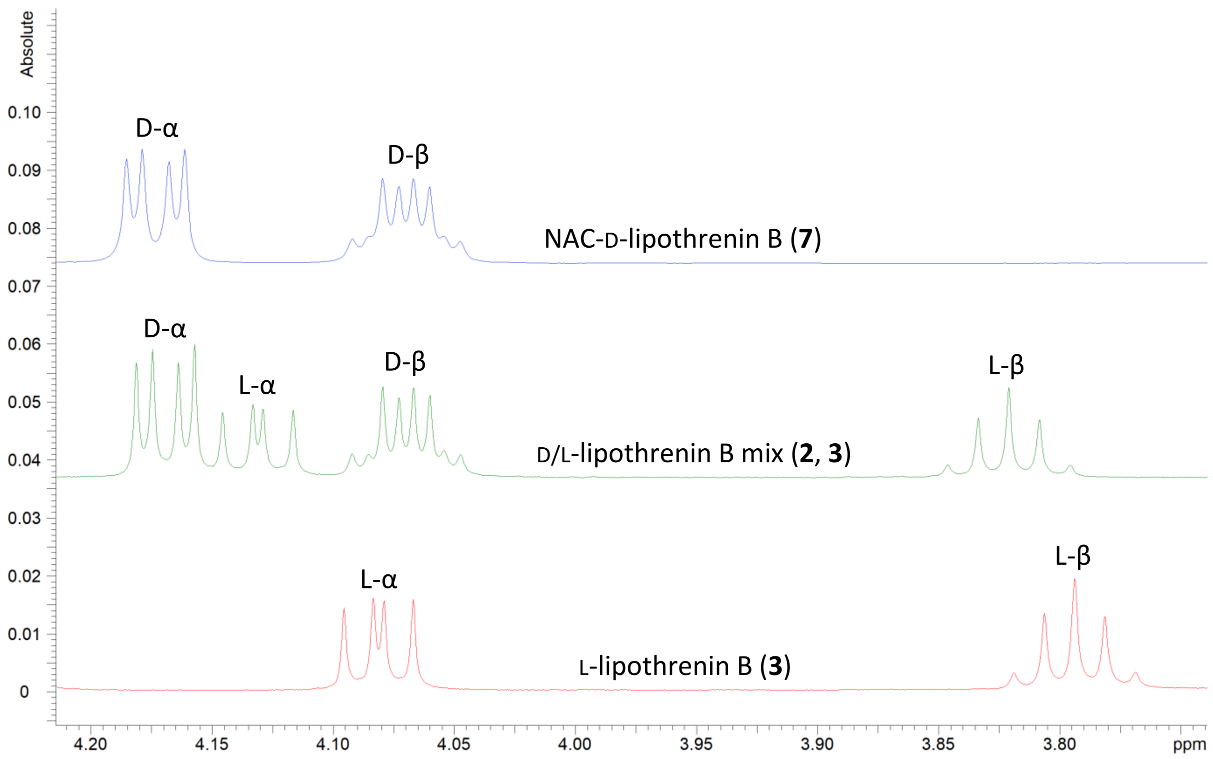

Figure S95. ^{1}H NMR spectra of compound **7**, compound mixture **2**, **3** and compound **3** isolated from deletion mutant I6_ΔlitM. (500 MHz, DMSO-d_6); compound **7** identifies shift and peak splitting of protons belonging to D-threonine previously determined by Marfey's method; L-threonine proton shifts and peak splitting are determined *vis versa* and identifying the lipothrenin B derivative from I6_ΔlitM as L-lipothrenin B (**3**).

References

1. M. Myronovskyi, B. Rosenkränzer, S. Nadmid, P. Pujic, P. Normand and A. Luzhetskyy, *Metab. Eng.*, 2018, **49**, 316-324.
2. Y. Ahmed, Y. Rebets, M. R. Estévez, J. Zapp, M. Myronovskyi and A. Luzhetskyy, *Microbial cell factories*, 2020, **19**, 1-16.
3. F. Flett, V. Mersinias and C. P. Smith, *FEMS Microbiol. Lett.*, 1997, **155**, 223-229.
